# Topological engineering of covalent organic frameworks enhances photoactivation of electron donor–acceptor complexes via conformational locking

**DOI:** 10.1093/nsr/nwag183

**Published:** 2026-03-24

**Authors:** Yifan Dong, Yimin Pan, Zhenze Yang, Ailin Pan, Wenjie Shi, Haifeng Zheng, Wenbin Lin

**Affiliations:** Frontier Institute of Science and Technology, Xi’an Jiaotong University, Xi’an 710054, China; Department of Chemistry, Westlake University, Hangzhou 310039, China; Frontier Institute of Science and Technology, Xi’an Jiaotong University, Xi’an 710054, China; Frontier Institute of Science and Technology, Xi’an Jiaotong University, Xi’an 710054, China; Frontier Institute of Science and Technology, Xi’an Jiaotong University, Xi’an 710054, China; MOE International Joint Laboratory of Materials Microstructure, Institute for New Energy Materials and Low Carbon Technologies, School of Materials Science and Engineering, Tianjin University of Technology, Tianjin 300384, China; Frontier Institute of Science and Technology, Xi’an Jiaotong University, Xi’an 710054, China; Department of Chemistry, Westlake University, Hangzhou 310039, China; Department of Chemistry, Westlake University, Hangzhou 310039, China

**Keywords:** electron donor–acceptor complex, photoactivation, covalent organic frameworks, C–H functionalization, [3+2] cyclization

## Abstract

Electron donor–acceptor (EDA) complex photoactivation offers a valuable route for organic synthesis, yet the development of efficient catalytic systems remains a significant challenge. We present a novel strategy based on topological conformational locking within covalent organic frameworks (COFs) to engineer high-performance donor platforms. Specifically, we constructed a three-dimensional (3D) COF and two two-dimensional (2D) analogues from the conformationally flexible building block 4,4′,4″,4″′-(1,4-phenylenebis(azanetriyl))tetrabenzaldehyde (PATB). Unlike the 2D structures, the 3D architecture intrinsically restricts molecular flexibility, stabilizing a locked conformation that significantly enhances acceptor binding affinity and facilitates efficient EDA complex formation and electron transfer. The 3D-PATB COF exhibits superior photocatalytic activity across a range of transformations, including C–H functionalization and [3+2] cyclization, and supports gram-scale synthesis with outstanding structural stability. Both experimental and computational analyses underscore the critical role of conformational locking in boosting catalytic performance, establishing topological engineering as a powerful approach for advancing EDA complex photochemistry.

## Teaser

Topological design of COFs can lock molecular conformation, enhancing donor–acceptor interactions and enabling efficient light-driven chemical transformations.

## INTRODUCTION

Electron donor–acceptor (EDA) complexes have recently emerged as a powerful strategy for photoredox chemical transformations [1–6]. Upon irradiation, these complexes undergo single-electron transfer (SET), generating radical cation–anion pairs that drive subsequent reactions. However, stoichiometric EDA systems require precisely matched donor–acceptor pairs, with both components necessarily incorporated into the final product, which inherently limits reaction diversity. In contrast, catalytic EDA approaches enable dynamic self-assembly between catalytic donors/acceptors and substrates, forming photoactive charge-transfer complexes [7]. Upon light exposure, these complexes undergo intra-complex SET, triggering substrate fragmentation and catalyst regeneration. Since the seminal work by Fu and coworkers in 2019 [8], a variety of catalytic donors and acceptors have been developed, significantly broadening synthetic applicability [9–18]. Despite these advances, the weak non-covalent interactions governing EDA complex formation and conformational flexibility of donors/acceptors often necessitate high catalyst loading (>10 mol%), severely limiting turnover efficiency.

**Figure 1.**
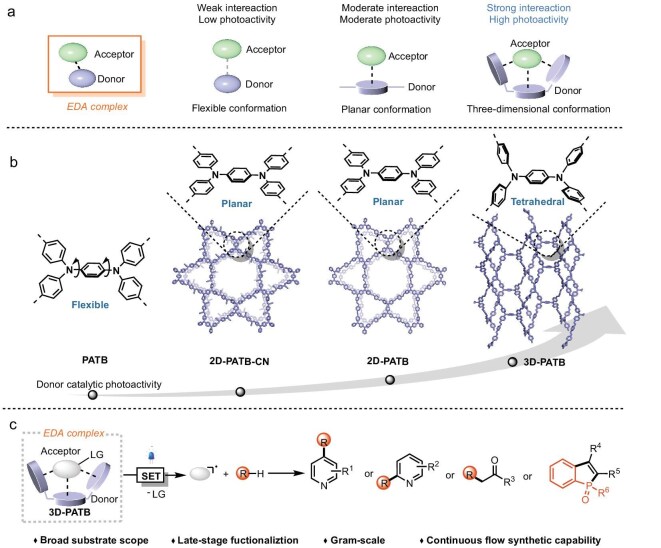
Conformational locking enhances EDA complex photoactivation. (a) Conformation effects in EDA complex photoactivity. (b) Topology optimization of PATB. (c) This work with 3D-PATB COF as donor catalyst for photocatalytic C–H functionalization and cascade [3+2] cyclization.

A critical determinant of EDA efficacy lies in the electronic coupling and topological alignment of donor–acceptor moieties (Fig. 1a) [3]. Current donor/acceptor catalysts suffer from undesired conformational flexibility, requiring extensive structure-activity optimization [14]. Covalent organic frameworks (COFs) are an emerging class of crystalline porous materials, in which organic building blocks are covalently linked to form two-dimensional (2D) or three-dimensional (3D) networks [19–24]. With their periodic architectures, low densities, large surface areas, and high stability, COFs represent a highly designable and functional materials platform with diverse applications, including gas storage and separation [25–29], heterogeneous catalysis [30–47], energy storage [48–52], proton conductivity [53–55], molecular sensing [56–59], and drug delivery [60–62]. In COF-based sustainable catalysis, structural alignment, non-covalent interactions, microstructural topology, surface area, and electron density distribution can vary significantly among isomeric COFs, leading to distinct adsorptive and catalytic performances [63,64]. Despite these advantages, the systematic exploration of COF topology for EDA photocatalysis remains largely uncharted.

In this work, we strategically selected 4,4′,4″,4^‴^-(1,4-phenylenebis(azanetriyl))tetrabenzaldehyde (PATB) as the functional node and synthesized a pair of 2D COFs (2D-PATB-CN and 2D-PATB) and a 3D COF (3D-PATB) [65]. All three COFs function as donor catalysts for EDA complex photocatalysis. Notably, the 3D-PATB COF, featuring a diamond topology, outperforms 2D analogs and homogeneous counterparts (Fig. 1b). It efficiently catalyzes diverse C–H functionalization and radical cascade [3+2] cyclization reactions and facilitates late-stage modification of bioactive molecules, including Nikethamide, Admiral, Bisacodyl, Boscalid, and Naproxen methyl ester (Fig. 1c). Additionally, the process is scalable under both batch and continuous-flow conditions, with the catalyst demonstrating excellent reusability and minimal loss of activity. Control experiments, solid-state ultraviolet–visible (UV–vis) absorption spectroscopy, photophysical analysis, and density functional theory (DFT) calculations provide mechanistic insights into the reaction and the role of topology. The unique tetrahedral conformation of PATB moieties in the 3D COF enhances acceptor affinity and facilitates efficient electron transfer. This work underscores the significance of topology engineering in designing COFs as sustainable heterogeneous photocatalyst.

## RESULTS AND DISCUSSION

### Synthesis and structural characterization

**Figure 2.**
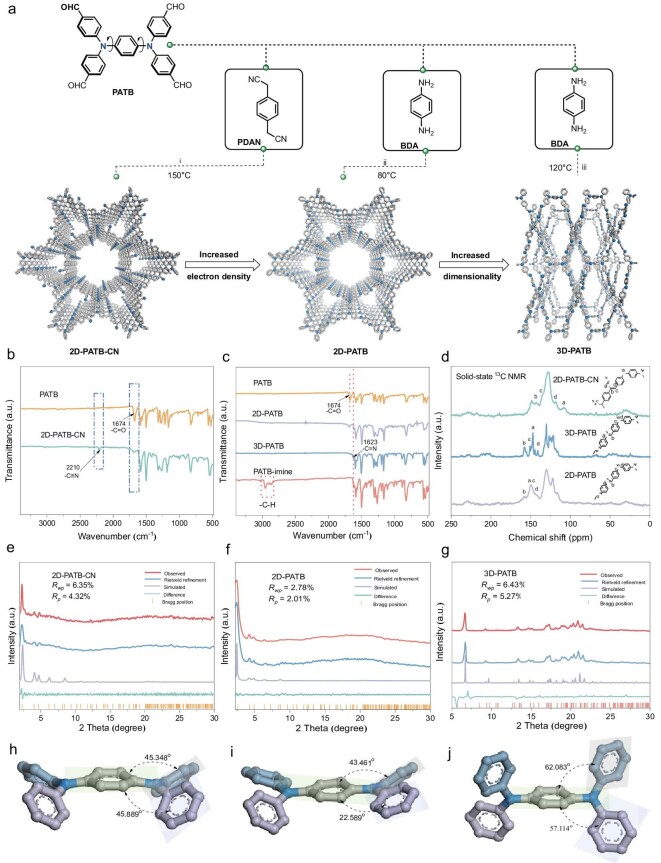
Synthesis and characterization of 2D-PATB-CN, 2D-PATB, and 3D-PATB COFs. (a) Synthesis of three PATB-based COFs (2D-PATB-CN, 2D-PATB, and 3D-PATB). Fourier transform infrared (FTIR) spectra of 2D-PATB-CN (b), 2D-PATB (c), and 3D-PATB (c). (d) Solid-state ^13^C NMR spectra of 2D-PATB-CN, 2D-PATB, and 3D-PATB. Rietveld refinement of 2D-PATB-CN (e), 2D-PATB (f) and 3D-PATB (g) COFs. The dihedral angle information of PATB in 2D-PATB-CN (h), 2D-PATB (i) and 3D-PATB (j) COFs was analyzed.

Inspired by previous elegant work on topologically engineered COFs [65], we selected the conformationally polymorphic 4,4′,4″,4″′-(1,4-phenylenebis(azanetriyl))tetrabenzaldehyde (PATB) as the structural backbone, combining it with 2,2′-(1,4-phenylene)diacetonitrile (PDAN) or benzene-1,4-diamine (BDA) as molecular linkers. Three COFs, 2D-PATB-CN, 2D-PATB, and 3D-PATB, were synthesized via solvothermal methods (Fig. 2a). For 2D-PATB-CN, a Pyrex tube was charged with PATB, PDAN, and Cs_2_CO_3_ in *o*-dichlorobenzene and heated at 150°C for 72 h. The resulting solid was filtered, subjected to Soxhlet extraction with tetrahydrofuran (THF) and ethanol for 3 days, and vacuum-dried at 100°C for 10 h to yield a powdered product. For 2D-PATB and 3D-PATB, PATB and BDA were mixed in either *o*-dichlorobenzene: *t*-butanol (v/v, 3:7) or *o*-dichlorobenzene: mesitylene (v/v, 1:1) with acetic acid (3 M for 2D-PATB; 9 M for 3D-PATB) in Pyrex tubes. The mixtures were heated at 80°C (for 2D-PATB) and 120°C (for 3D-PATB) for 72 h. The resulting solids were filtered, washed via Soxhlet extraction with THF and dichloromethane for 2 days, and vacuum-dried at 120°C for 12 h, yielding powdered 2D-PATB and 3D-PATB.

The formation of 2D-PATD-CN, 2D-PATB, and 3D-PATB COFs was supported by Fourier transform infrared (FT-IR) spectroscopy (Fig. 2b and c) and solid-state nuclear magnetic resonance (ssNMR) spectroscopy (Fig. 2d). For 2D-PATB-CN, the C=O stretching vibration of the aldehyde group at 1674 cm^−1^ diminished, while the C=C stretching vibration at 1600 cm^−1^ intensified, accompanied by a new peak at 2210 cm^−1^ corresponding to the CN stretching vibration. ssNMR spectroscopy further supported this assignment, showing characteristic peaks around 107.3 ppm for vinyl nitrile carbon atoms. These spectroscopic data support the formation of C=C bonds via Knoevenagel condensation and the synthesis of an sp^2^-carbon–conjugated COF. 2D-PATB and 3D-PATB COFs exhibited a stretching vibration at 1623 cm^−1^ in the FT-IR spectra and a new ^13^C resonance at ∼158 ppm in the ssNMR spectra, conforming the successful formation of imine linkages. The pore structures of these crystalline frameworks were evaluated using nitrogen sorption measurements at 77 K. Brunauer–Emmett–Teller analysis revealed a specific surface area of 251 m²/g for 2D-PATB-CN (Fig. S5). Quenched solid density functional theory calculations of the pore size distribution showed two distinct peaks at 1.4 and 2.0 nm (Fig. S5). 2D-PATB exhibited a specific surface area of 516 m^2^/g (Fig. S6), with narrow pore distributions centered at 1.2 and 2.5 nm (Fig. S6). In contrast, 3D-PATB-COF with a multiply interpenetrated structure adsorbed only negligible amounts of nitrogen due to its complex structural architecture (Fig. S7). Thermogravimetric analysis demonstrated that all three COFs exhibit excellent thermal stability under nitrogen atmosphere (Figs S8–S10), with 3D-PATB showing the highest thermal stability, maintaining structural integrity up to ∼500°C.

The long-range order of the COFs was confirmed by powder X-ray diffraction (PXRD) analysis (Fig. 2e–g). The experimental PXRD pattern of 2D-PATB-CN closely matched the simulated pattern, with Rietveld refinement indicating excellent agreement (*R*_p_ = 4.32% and *R*_wp_ = 6.35%). Similarly, the experimental patterns of 2D-PATB and 3D-PATB aligned well with the simulated results from Pawley refinement (2D-PATB: *R*_p_ = 2.01%, *R*_wp_ = 2.78%; 3D-PATB: *R*_p_ = 5.27%, *R*_wp_ = 6.43%). Structural modeling using Materials Studio provided further insights into the conformational characteristics of the PATB units relevant to catalytic activity in the COFs (Fig. 2h–j). In both 2D-PATB-CN and 2D-PATB, the PATB moiety adopted a planar conformation. In contrast, the PATB moiety in 3D-PATB exhibited an approximately tetrahedral conformation, with two highly distorted propeller-like arrangements around the nitrogen atoms.

### Photophysical and electrochemical properties

Photophysical and electrochemical measurements were conducted to assess the optical properties and charge separation efficiencies of the COFs (Figs S11–S15). The COFs exhibited broad absorption across the visible range (400–600 nm), indicating strong light-harvesting capabilities (Fig. S11). The optical bandgaps, estimated from diffuse reflectance spectra using the Kubelka–Munk function (Fig. S12), were determined to be 2.15 eV for 2D-PATB-CN, and 2.39 eV for both 2D-PATB and 3D-PATB. The energy band structures were further examined using Mott–Schottky analysis (Fig. S14). The flat band potentials, extrapolated from the linear regions of the plots, gave conduction band (CB) positions of −1.24, −1.34, and −1.47 V vs. saturated calomel electrode (SCE) for 2D-PATB-CN, 2D-PATB, and 3D-PATB, respectively. Using the relationship *E*_g_ = *E*_VB_ − *E*_CB_ the corresponding valence band (VB) positions were calculated as 0.91, 1.05, and 0.92 V vs. SCE (Fig. S15).

To assess charge separation efficiency under photocatalytic conditions, light-dependent electrochemical measurements were performed (Fig. S12). Photocurrent measurements showed a pronounced increase in photocurrent density under illumination compared to dark conditions, with 3D-PATB exhibiting the highest values. This highlights its superior charge carrier generation and separation capability. Consistent insights were provided by electrochemical impedance spectroscopy. The associated Nyquist plot revealed a smaller semicircle for 3D-PATB, indicating lower charge transfer resistance (Fig. S12). Additionally, the reduction potentials for 2D-PATB, 2D-PATB-CN, and 3D-PATB were measured to be −0.85, −0.83, and −0.81 V vs. SCE, respectively (Fig. S13).

### Photocatalytic activities

**Figure 3.**
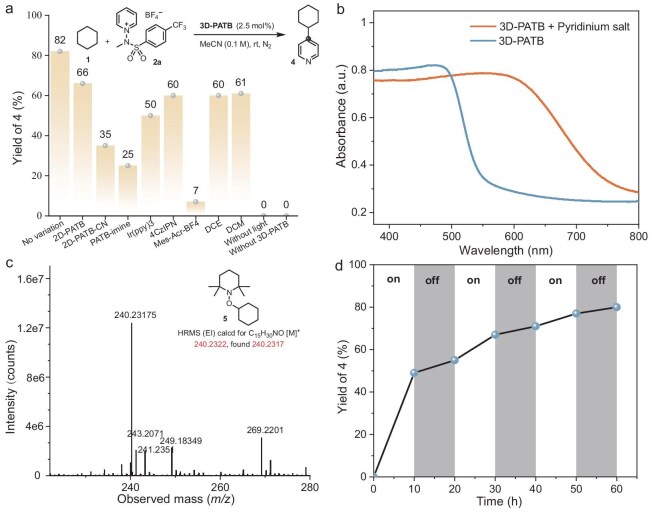
C–H pyridylation reaction and control experiments. (a) C–H pyridylation between cyclohexane **1** and *N*-aminopyridinium salt **2** with different catalysts. (b) Solid UV–vis spectroscopy of 3D-PATB COF and 3D-PATB COF with **2**. (c) High-resolution mass spectrometry (HRMS) of radical capture experiment. (d) Light on/off experiments for the synthesis of **4**.

The distinctive properties of PATB-based COFs, including their crystalline frameworks, chemical stability, tunable topological conformations, high charge separation efficiency, and accessible reduction potentials provide them promising donor catalyst platforms for sustainable EDA complex-mediated photoredox catalysis. To evaluate their photocatalytic performance, we selected the C–H pyridylation of cyclohexane **1** and *N*-aminopyridinium salt **2a** as a model reaction [66]. Using 3D-PATB as the catalyst (2.5 mol% loading) under blue LED irradiation at room temperature, the desired product **4** was obtained in 82% yield. In contrast, 2D-PATB, 2D-PATB-CN, and PATB-imine produced **4** in 66%, 35%, and 25% yields, respectively, substantially lower than the performance of 3D-PATB. Notably, several representative molecular photo-sensitizers, including Ir(ppy)_3_, 4CzIPN, and Mes-Acr-BF_4_, were also tested under identical conditions, but all exhibited inferior activity (Fig. 3a). Solvent screening revealed acetonitrile as the most effective medium for this transformation. Furthermore, control experiments confirmed that both light irradiation and the presence of the 3D-PATB catalyst were essential for successful product formation (Fig. 3a).

### Mechanistic studies

To investigate the reaction mechanism, we conducted solid and liquid-state UV–vis absorption spectroscopy, steady-state fluorescence, radical capture experiments, light on/off studies, and quantum yield measurements. UV–vis analysis was performed to confirm the formation of an EDA complex between the 3D-PATB COF donor catalyst and the pyridinium salt (Fig. 3b). The UV–vis spectrum of 3D-PATB displayed absorption across the 350–700 nm range. Upon adsorption of substrate **2a** into the 3D-PATB framework, a significantly enhanced absorption band appeared between 500 and 700 nm, indicative of EDA complex formation between the COF and **2a**. Job’s plot analyses provided further evidence for this interaction. UV–vis spectra were recorded for mixtures of 3D-PATB and the pyridinium substrate **2a** at systematically varied donor/acceptor ratios. The observed absorption changes were composition-dependent, with a maximum at a 1:1 ratio, strongly supporting the formation of a well-defined 1:1 EDA complex (Fig. S26). Steady-state photoluminescence (PL) titration experiments were performed by gradually adding pyridinium acceptors to suspensions of the three COFs. Notably, the emission intensity of 3D-PATB increased continuously and significantly with higher acceptor concentration, a hallmark of strong EDA interactions that alter excited-state behavior. In contrast, 2D-PATB and 2D-PATB-CN showed little to no PL enhancement under identical conditions, indicating much weaker donor–acceptor interactions (Fig. S28).

Radical trapping experiments supported a SET reaction mechanism. The addition of TEMPO as a radical scavenger completely suppressed the formation of product **4**. HRMS confirmed the presence of the TEMPO-trapped radical species **5** (Fig. 3c). Furthermore, light on/off experiments revealed that product **4** formation persisted even in the absence of light (Fig. 3d), suggesting a radical chain propagation pathway. Notably, the quantum yield (*Φ*) for the reaction was determined to be 1.3 under standard conditions (Fig. S34). A value exceeding unity indicates that a single absorbed photon initiates more than one catalytic turnover. This observation is consistent with, and provides quantitative support for, a photo-initiated radical chain mechanism.

**Figure 4.**
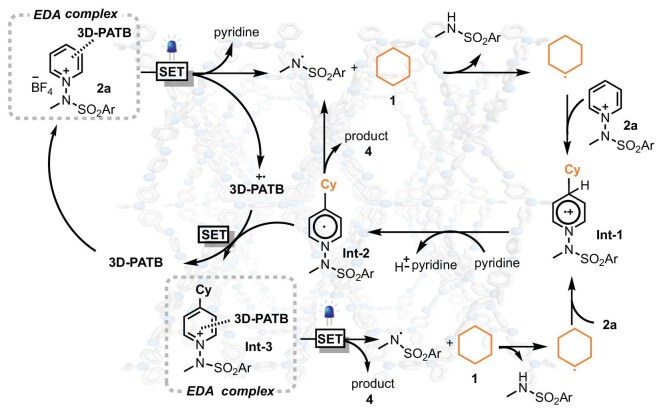
The proposed reaction mechanism for 3D-PATB-catalyzed C–H pyridylation between cyclohexane **1** and *N*-aminopyridinium salt **2**.

Based on these experimental findings and prior literature precedents [7,66], a plausible reaction mechanism is proposed (Fig. 4). Initially, 3D-PATB interacts with pyridinium salt **2a** to form a photoactive EDA complex. Upon light irradiation, intra-complex SET occurs from 3D-PATB to **2a**, generating a catalyst radical cation, neutral pyridine, and an amidyl radical. The amidyl radical abstracts a hydrogen atom from cyclohexane **1**, generating a carbon-centered radical. This radical selectively attacks the C4-position of pyridinium salt **2a**, forming the radical adduct intermediate **Int-1**. Subsequent deprotonation of **Int-1** by pyridine yields intermediate **Int-2**, which can proceed via two possible pathways: (1) Electron transfer pathway: **Int-2** donates an electron to the catalyst radical cation, forming **Int-3** and regenerating 3D-PATB. A subsequent SET between 3D-PATB and **Int-3** yields the final product **4** and *N*-centered radical, thereby completing the catalytic cycle. (2) Fragmentation pathway: **Int-2** undergoes fragmentation to produce **4** and regenerate the amidyl radical.

### DFT calculations

**Figure 5.**
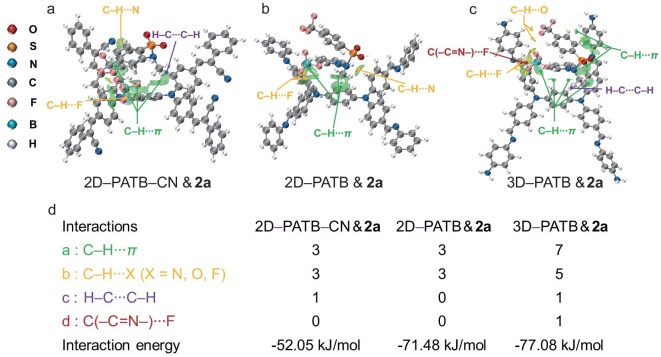
DFT calculation studies. The interactions between substrate **2a** and PATB moiety in 2D-PATB-CN (a), 2D-PATB (b), and 3D-PATB (c), individually. (d) The types and quantities of interactions and interaction energies.

Additionally, we performed DFT calculations to investigate the interactions between pyridinium salt **2a** and the PATB moiety in the COFs, providing insights into how topological structure influences catalytic activity (Fig. 5). In 2D-PATB-CN and 2D-PATB, the PATB moiety adopts a planar conformation, with **2a** interacting primarily through C–H···π, C–H···N, C–H···F, and C–H···C–H interactions. In contrast, the tetrahedral conformation of PATB in 3D-PATB preserve these interactions while introducing additional C–H···O and C···F contacts (Fig. 5a–c). Further computational analysis revealed that **2a** exhibits a stronger binding affinity to 3D-PATB, with an interaction energy of −77.08 kJ/mol, compared to −71.48 kJ/mol for 2D-PATB and −52.05 kJ/mol for 2D-PATB-CN (Fig. 5d). These enhanced interactions in the 3D framework promote more robust EDA complex formation and facilitate efficient electron transfer.

### Photocatalytic C–H functionalization and [3+2] cyclization

**Figure 6.**
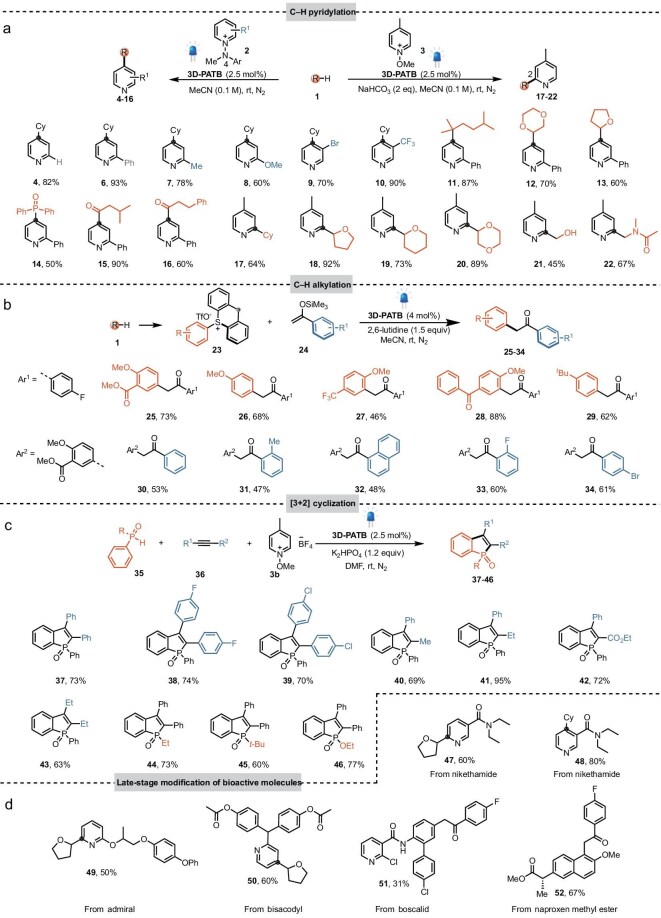
The generality of the catalytic system. Substrate scope of C–H pyridylation (a), C–H alkylation (b), and [3+2] cyclization (c) reactions. (d) Late-stage modification of bioactive molecules.

The versatility of 3D-PATB as a donor catalyst was evaluated in three representative EDA complex-mediated catalytic reactions, including C–H pyridylation [17,66], formal C–H alkylation [14] and radical cascade [3+2] cyclization [67,68], under mild conditions (visible light irradiation and ambient temperature). As shown in Fig. 6a, 2.5 mol% 3D-PATB efficiently catalyzed the C–H pyridylation of hydrocarbons with pyridinium salts **2** and **3**, yielding high-value pyridine derivatives **4–22**. Pyridinium salts bearing substituents such as phenyl (**2b**), methyl (**2c**), methoxy (**2d**), and trifluoromethyl (**2e**) groups were tolerated, affording C4-selective products **4–10** in 60%–93% yields. Hydrocarbons, including 2,5-dimethylhexane, 1,4-dioxane, tetrahydrofuran, and non-hydrocarbon substrates such as diphenylphosphine oxide and aldehydes reacted with **2** to give products **11–16** in 50%–90% yields. Reaction of **3** with hydrocarbons proceeded smoothly to form C2-selective products **17–22** in 45%–92% yields.

At 4 mol% catalyst loading, 3D-PATB also promoted the formal C–H al-kylation reaction of arenes with silyl enol ethers **24**, producing acetophenone derivatives **25–34** (Fig. 6b). Anisole derivatives bearing substituents such as trifluoromethyl, methoxycarbonyl, and benzoyl were compatible, affording products **25–28** in 46%–88% yields. The reaction of 4-*tert*-butylbenzene with **24** provided product **29** in 62% yield. Notably, silyl enol ethers with electron-withdrawing substituents (**24e** and **24f**) gave higher yields than those with electron-donating groups (**24b–24d**).

In addition, 3D-PATB at 2.5 mol% loading catalyzed the radical cascade [3+2] cyclization of phosphine oxides **35** with alkynes **36** (Fig. 6c). Substituted alkynes (**36a–36 g**) reacted with **35** to furnish benzo[b]phosphole oxides **37–43** in 63%–95% yields. Phosphine oxide bearing both alkyl and alkyloxy substituents reacted with **36a** to afford products **44, 45**, and **46** in 73%, 60%, and 77% yields, respectively.

The potential of 3D-PATB for late-stage functionalization was further demonstrated through C–H pyridylation and alkylation of complex drug-like or bioactive molecules (Fig. 6d). Substrates such as Nikethamide, Admiral, Bisacodyl, Boscalid, and Naproxen methyl ester were successfully converted to products **47–52** in 31%–80% yields.

**Figure 7.**
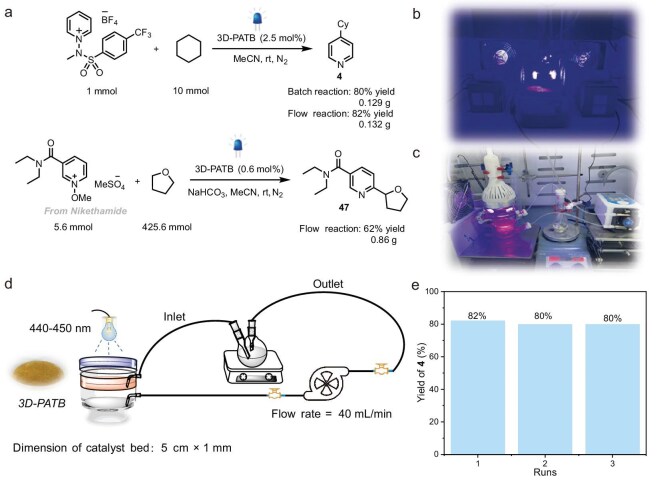
Gram-scale reaction and recycle experiments. (a) The synthesis of products **4** and **47** in batch and continuous flow reaction. (b) Batch set-up reaction. (c and d) Continuous flow set-up reaction. (e) The reaction yields in three consecutive cycles of the synthesis of 4-cyclohexylpyridine **4** (0.1 mmol scale).

To assess practical applicability, gram-scale C–H pyridylation reactions of cyclohexane and tetrahydrofuran were performed under batch and continuous flow conditions (Fig. 7). Products **4** and **47** were obtained in 82% and 62% yields, respectively (Fig. 7a). Remarkably, continuous-flow synthesis of compound **47** achieved exceptional catalytic efficiency with 0.6 mol% catalyst loading in a 5 cm × 1 mm fixed-bed reactor under optimized conditions (Fig. 7d). The robustness of the 3D-PATB catalyst was confirmed through recyclability tests in C–H pyridylation, C–H alkylation, and [3+2] cyclization. After each reaction, the catalyst was recovered by filtration, washed with acetonitrile, and directly reused in subsequent cycles, with minimal yield loss over three cycles (Figs S29–S32). PXRD and IR analyses of the recovered 3D-PATB confirmed retention of its crystalline structure (Figs S33–S34), underscoring its excellent stability and reusability in sustainable EDA complex mediated photoredox catalysis.

## CONCLUSION

In this work, we have developed a sustainable donor catalyst, 3D-PATB COF, through a novel conformational lock strategy. The tetrahedral conformation of PATB in 3D-PATB COF exhibits enhanced affinity for acceptors, enabling efficient EDA complex formation and facilitating electron transfer. This catalyst effectively promotes C–H pyridylation, formal C–H alkylation and radical cascade [3+2] cyclization to afford structurally diversified pyridines, acetophenones and benzo[b]phosphole oxides in good to excellent yields under mild conditions. Furthermore, we demonstrate its applicability in late-stage modification of bioactive molecules, gram-scale synthesis, and catalyst recyclability in both batch and continuous flow systems. These findings underscore the pivotal role of topology engineering in designing porous COFs as robust heterogeneous photocatalysts for sustainable organic synthesis.

## METHODS

### General procedure for C4-selective C–H pyridylation

*N*-aminopyridinium salt **2** (0.1 mmol), 3D-PATB (2.5 mol% catalyst loading based on the PATB linker) and hydrocarbon **1** (1 mmol) were mixed in anhydrous acetonitrile (MeCN, 1.0 mL) in a sealed test tube. The mixture was stirred under blue LED irradiation (440–450 nm) at room temperature under a nitrogen atmosphere for 48 h. After that, 3D-PATB was filtered off, the solvent was removed under vacuum, and the residue was purified by column chromatography on silica gel to give products **4–16** in 50%–93% yields.

### General procedure for C2-selective C–H pyridylation

*N*-Methoxy pyridinium methylsulfate **3a** (0.1 mmol), 3D-PATB (2.5 mol% catalyst loading based on the PATB linker), hydrocarbon **1** (7.2 mmol), and NaHCO_3_ (0.2 mmol) were mixed in anhydrous MeCN (1.0 mL) in a sealed test tube. The mixture was stirred under blue LED irradiation (440–450 nm) at room temperature in a nitrogen atmosphere for 24 h. After that, 3D-PATB was filtered off, the solvent was removed under vacuum, and the residue was purified by column chromatography on silica gel to give products **17–22** in 45%–92% yields.

### General procedure for formal C–H alkylation

Aryl sulfonium salts **23** (0.05 mmol), silyl enol ethers **24** (0.25 mmol), 2,6-lutidine (0.075 mmol), and 3D-PATB (4.0 mol% catalyst loading based on the PATB linker) were mixed in anhydrous MeCN (1.0 mL) in a sealed test tube. The mixture was stirred under blue LED irradiation (390–400 nm) at room temperature in a nitrogen atmosphere for 48 h. After that, 3D-PATB was filtered off, the solvent was removed under vacuum, and the residue was purified by column chromatography on silica gel to afford products **25–34** in 46%–88% yields.

### General procedure for [3+2] cyclization

*N*-methoxy-4-methylpyridinium tetrafluoroborate **3b** (0.2 mmol), alkyne **36** (0.1 mmol), phosphine oxide **35** (0.2 mmol) and potassium phosphate dibasic (0.12 mmol), 3D-PATB (2.5 mol% catalyst loading based on the PATB linker) were mixed in anhydrous *N,N-*dimethylformamide (DMF, 1.0 mL) in a sealed test tube. The mixture was stirred under blue LED irradiation (440–450 nm) at room temperature in a nitrogen atmosphere for 48 h. After that, 3D-PATB was filtered off and the solvent was diluted, extracted with ethyl acetate three times. The organic layer was dried over sodium sulfate, filtered, and concentrated under reduced pressure, and purified by flash column chromatography on silica gel to afford the desired product **37–46** in 60%–95% yields.

## Supplementary Material

nwag183_Supplemental_File
