# Supplementary material for: Topological engineering of covalent organic frameworks enhances photoactivation of electron donor–acceptor complexes via conformational locking
Source: Natl Sci Rev. 2026 Mar 24;13(11):nwag183. doi: 10.1093/nsr/nwag183 (PMC13292214; doi:10.1093/nsr/nwag183)
Supplement: nwag183_Supplemental_File [file nwag183_supplemental_file.pdf]

Supporting Information

Topological Engineering of Covalent Organic Frameworks Enhances Photoactivation of Electron Donor–Acceptor Complexes via Conformational Locking

Yifan Dong[1,3], Yimin Pan[1], Zhenze Yang[1], Ailin Pan[1], Wenjie Shi[2,*], Haifeng Zheng[1,3,*], Wenbin Lin[3,*]

[1] Frontier Institute of Science and Technology, Xi'an Jiaotong University, Xi'an, Shaanxi 710054, China

[2] MOE International Joint Laboratory of Materials Microstructure, Institute for New Energy Materials and Low Carbon Technologies, School of Materials Science and Engineering, Tianjin University of Technology, Tianjin 300384, China

[3] Department of Chemistry, Westlake University, Hangzhou 310030, Zhejiang, China

*Corresponding Authors: wjshi@email.tjut.edu.cn; haifengzheng@xjtu.edu.cn; linwenbin@westlake.edu.cn

Table of Contents

S1. General Information……………………………………………………………………...4

S2. Synthesis of model compound and COFs……………………………………………….5

S2.1 Synthesis of model compound PATB–imine.. 5

S2.2 Synthesis of 2D–PATB.. 5

S2.3 Synthesis of 3D–PATB.. 6

S2.4 Synthesis of 2D–PATB–CN 7

S3. Characterization of COFs 7

S3.1 N_2 adsorption analyses... 7

S3.2 Thermogravimetric analysis.. 9

S3.3 Photophysical and electrochemical characterization 11

S3.4 Structure modeling and atomic coordinates of 2D–PATB–CN COF............................... 13

S3.5 Structure modeling and atomic coordinates of 2D–PATB COF...................................... 16

S3.6 Atomic coordinates of 3D–PATB COF 18

S4. General procedures for the synthesis of substrates………………………………………… 20

S4.1 Synthesis of *N*-protected aminopyridinium salts 20

S4.2 Synthesis of pyridinium salts.. 21

S4.3 Synthesis of aryl silyl enol ethers 21

S4.4 Synthesis of aryl sulfonium salts from simple arenes... 22

S4.5 Synthesis of aryl sulfonium salts from amide-containing or complex arenes 22

S5 Optimization of reaction conditions…………………………………………………….22

S6 General procedure and product characterization……………………………………..25

S6.1 General procedure and product characterization for C4–site-selective C–H pyridylation 26

S6.2 General procedure and product characterization for C2–site-selective C–H pyridylation 30

S6.3 General procedure and product characterization for formal C–H alkylation................... 32

S6.4 General procedure and product characterization for [3+2] cyclization 35

S6.5 General procedure for the scale-up synthesis of **4** 38

S7 Mechanistic Studies………………………………………………………………..39

S7.1 UV-Vis diffuse reflectance spectroscopy (DRS) analysis 39

S7.2 UV-Vis determination of EDA complexes 40
S7.3 UV-Vis analyses of the mixtures of 3D-PATB and **2a** with different ratios and Job Plot 43
S7.4 UV-Vis analyses of the mixtures of 3D-PATB and **3a** with different ratios and Job Plot 44
S7.5 Steady-state fluorescence analyses of the mixtures of 3D-PATB and **2a** with different ratios 45
S7.6 Radical capture experiments for C–H pyridylation 46
S7.7 Light on/off experiments for C–H pyridylation 48
S7.8 Quantum yield measurements 49
S7.9 Proposed reaction mechanism 52
S8 DFT computational studies..........53
S9 Recycle experiments and catalyst characterization..........80
S9.1 Recycle experiments for C–H pyridylation of **2a** with cyclohexane 80
S9.2 Recycle experiments for C–H pyridylation of **3a** with THF 81
S9.3 Recycle experiments for formal C–H alkylation of **23a** with **24a** 82
S9.4 Recycle experiments for [3+2] cyclization of **35a** with **36d** 83
S9.5 Characterization of the recovered catalyst from C–H pyridylation of **2a** with cyclohexane 85
S10 General procedure for continuous flow set-up reactions..........86
S11 NMR spectra..........88
S12 References..........112

S1. General Information

All the reactions and manipulations were carried out under N_2 with the use of a glovebox or Schlenk technique, unless otherwise indicated. The structures of the photocatalytic materials were confirmed by Powder X-ray diffraction (PXRD) with Cu Kα radiation (λ=1.5406Å) at a scanning rate of 10°/min. The Fourier Transform Infrared (FTIR) spectra were obtained from a FTIR-VERTEX70 spectrometer with potassium bromide and sample powder tablet. UV–Vis diffuse reflectance spectra (DRS) were carried out on Varian Cary 500 Scan UV–visible system. Electrochemical measurements were conducted with a BAS Epsilon electrochemical system in a conventional three electrode cell, using a Pt plate as the counter electrode and an Hg/Hg_2Cl_2 electrode (Saturated KCl) as the reference electrode, the active area is confined to 0.25 cm^2. The electrolyte was 0.2 M Na_2SO_4 aqueous solution without additive (pH 6.8). The working electrode was prepared on Fluorine doped Tin Oxide (FTO) glass that was cleaned by sonication in ethanol for 30 min and dried at 353 K. Then 5 mg sample was dispersed in 1 mL of ethanol/Nafion (50/50 V %) mixture by sonication to get a slurry mixture. The slurry was spread onto pre-treated FTO glass. Centrifugation was carried out using a centrifuge Minispin Rotor F-45-12-11. ^1H NMR spectra were recorded on commercial instruments (400 MHz). Chemical shifts are recorded in ppm relative to tetramethylsilane and with the solvent resonance as the internal standard ($CDCl_3$, δ = 7.26 ppm). Spectra are reported as follows: chemical shift, multiplicity (s = singlet, d = doublet, t = triplet, m = multiplet, comp = composite of magnetically non-equivalent protons, dd = doublet of doublets), coupling constants (Hz), integration and assignment. ^{13}C NMR data were collected on commercial instruments (101 MHz) with complete proton decoupling. Chemical shifts are reported in ppm from tetramethylsilane with the solvent resonance as internal standard ($CDCl_3$, δ = 77.0 ppm). High-resolution mass spectrometry (HRMS) was recorded on a commercial apparatus (ESI Source). *N*-protected aminopyridinium salts [1], pyridine *N*-oxides [2], aryl sulfonium salts [3] and (hetero)aryl silyl enol ethers [3] were prepared according to literature methods.

S2. Synthesis of model compound and COFs

S2.1 Synthesis of model compound PATB–imine

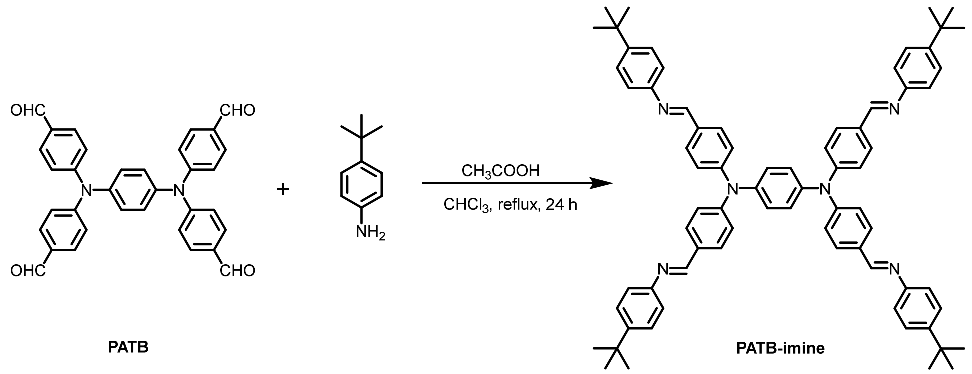

Figure S1. Synthesis of model compound PATB–imine.

PATB (524.0 mg, 1.0 mmol) and 4-tert-butylaniline (1.7 mL, 11 mmol) were added to a 50 mL round-bottom flask with 20 mL chloroform. Four drops of acetic acid were added to the mixture, which was then stirred under reflux overnight. After cooling to room temperature, the solvent was evaporated under reduced pressure. The resulting solid was collected, washed thoroughly with ethanol, and dried to afford PATB–imine as a yellow solid (820.0 mg, 78% yield) [4].

^{1}H NMR (400 MHz, $CDCl_3$) δ 8.44 (s, 4H), 7.85 (d, *J* = 8.4 Hz, 8H), 7.44 (d, *J* = 8.4 Hz, 8H), 7.24 (d, *J* = 8.4 Hz, 8H), 7.20 (d, *J* = 8.4 Hz, 8H), 1.36 (s, 36H).

^{13}C NMR (101 MHz, $CDCl_3$) δ 158.8, 149.7, 149.0, 142.9, 131.4, 130.1, 126.8, 126.2, 123.5, 120.7, 34.6, 31.6.

HRMS (ESI-TOF) calcd for $C_{74}H_{77}N_6^+$ ([M+H$^+$]) = 1049.6204, Found 1049.6203.

S2.2 Synthesis of 2D–PATB

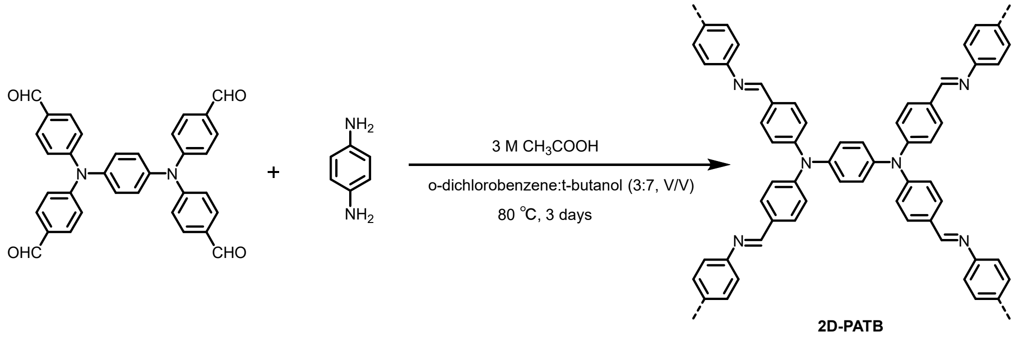

Figure S2. Synthesis of 2D–PATB

A Pyrex tube was charged with PATB (15.6 mg, 0.03 mmol), BDA (6.5 mg, 0.06 mmol), *o*-dichlorobenzene (*o*-DCB, 0.3 mL), *tert*-butanol (0.7 mL) and 3 mol/L aqueous acetic acid (0.1 mL). The mixture was degassed via three freeze-pump-thaw cycles, sealed under vacuum, and heated in an oven at 80 °C for 3 days. The resulting precipitate was filtered, exhaustively washed via Soxhlet extraction with tetrahydrofuran and dichloromethane for 2 days, and dried under vacuum at 120 °C for 12 h to yield 2D–PATB COF as a yellow powder (16.9 mg, 85% yield) [4].

S2.3 Synthesis of 3D–PATB

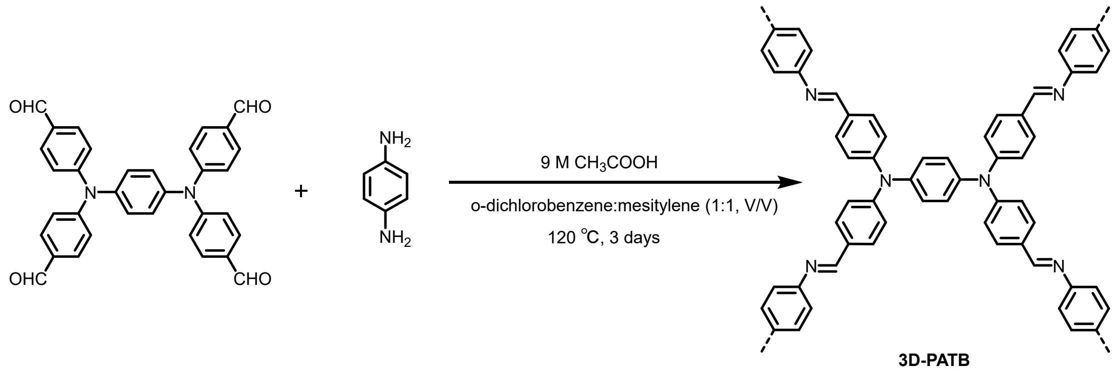

Figure S3. Synthesis of 3D–PATB

A Pyrex tube was charged with PATB (10.4 mg, 0.02 mmol), BDA (4.3 mg, 0.04 mmol), *o*-dichlorobenzene (*o*-DCB, 0.3 mL), mesitylene (0.3 mL) and 9 mol/L aqueous acetic acid (0.1 mL). The mixture was degassed via three freeze-pump-thaw cycles, sealed under vacuum, and heated in an oven at 120 °C for 3 days. The resulting precipitate was filtered, exhaustively washed via Soxhlet extractions with tetrahydrofuran and dichloromethane for 2 days, and dried under vacuum at 120 °C for 12 h to yield 3D–PATB COF as a yellow powder (6.3 mg, 23% yield) [4].

S2.4 Synthesis of 2D–PATB–CN

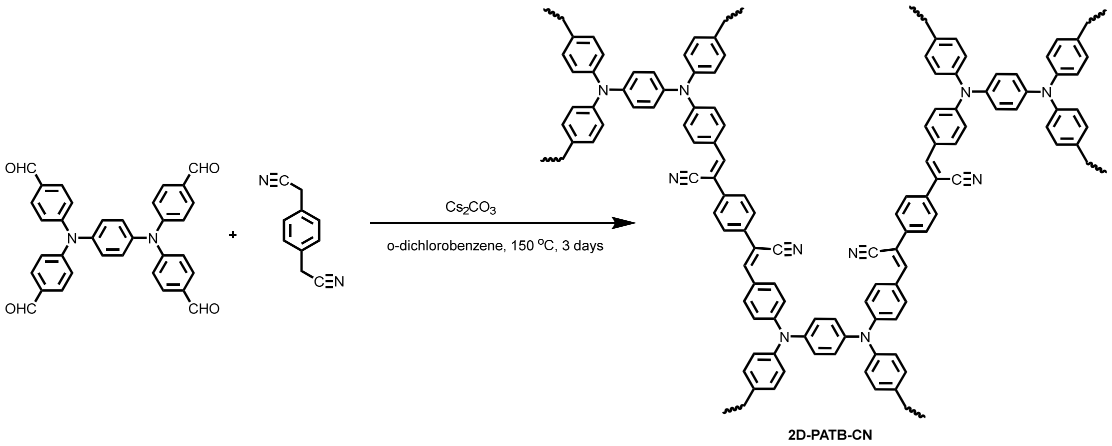

Figure S4. Synthesis of 2D–PATB–CN

A Pyrex tube was charged with PATB (15.6 mg, 0.03 mmol), 2,2'-(1,4-phenylene) di-acetonitrile (9.4 mg, 0.06 mmol), Cs_2CO_3 (44.0 mg, 0.135 mmol) and *o*-dichlorobenzene (0.5 mL). The mixture was degassed via three freeze-pump-thaw cycles, sealed under vacuum, and heated at 150 °C for 3 days. After cooling to room temperature, the precipitate was collected by centrifugation, washed via Soxhlet extraction with tetrahydrofuran and ethanol for 3 days, and dried under vacuum at 100 °C for 10 h to obtain 2D–PATB–CN as a red powder (25.3 mg, 73% yield).

S3. Characterization of COFs

S3.1 N_2 Adsorption Analyses

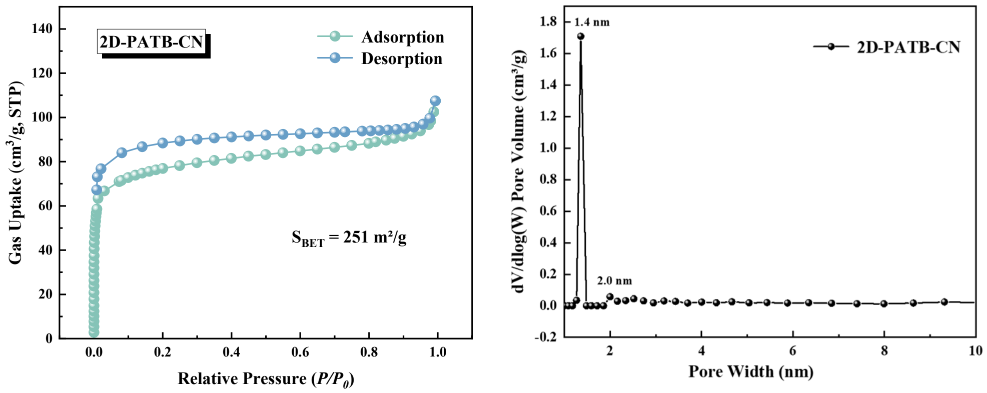

Figure S5. Nitrogen sorption isotherms (77 K) and pore size distribution of 2D-PATB-CN

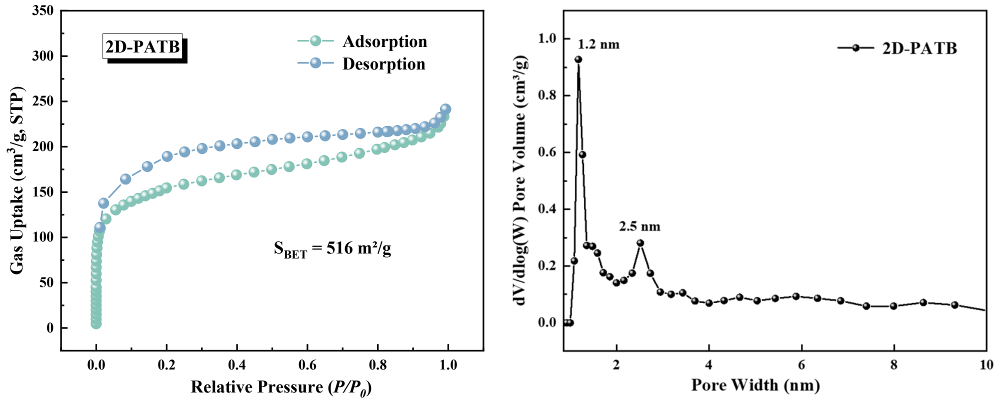

Figure S6. Nitrogen sorption isotherms (77 K) and pore size distribution of 2D-PATB.

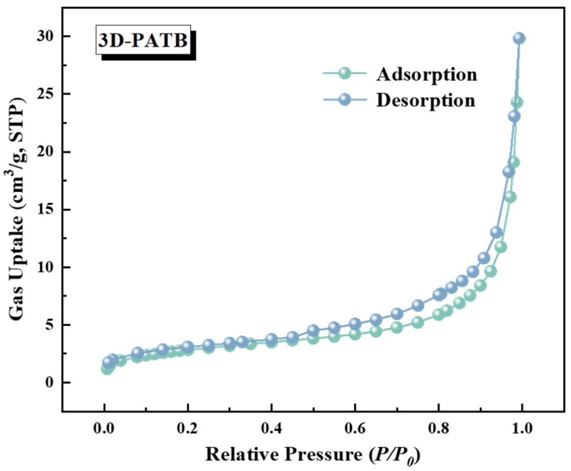

Figure S7. Nitrogen sorption isotherms (77 K) of 3D-PAPB.

S3.2 Thermogravimetric Analysis

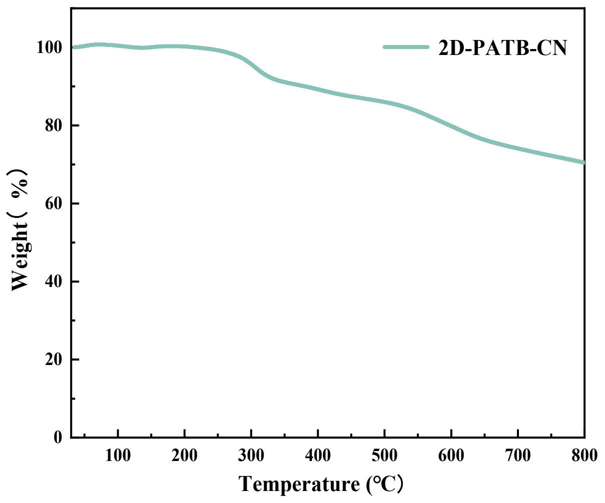

Figure S8. TGA plot of 2D–PATB-CN under N_2 atmosphere.

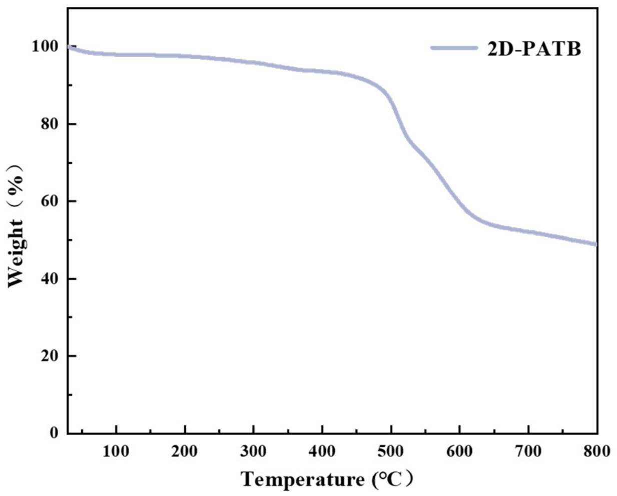

Figure S9. TGA plot of 2D–PATB under N_2 atmosphere.

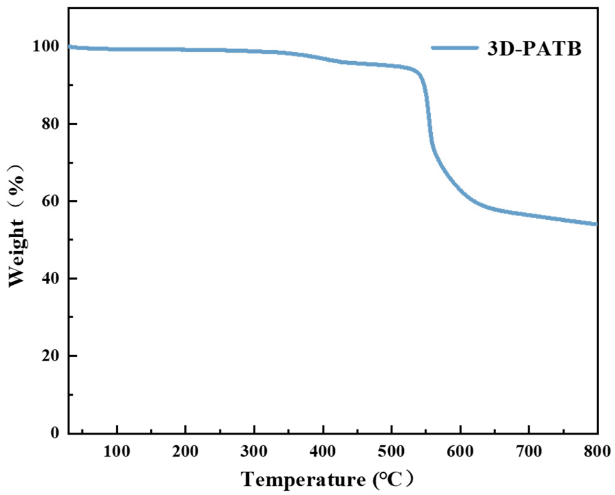

Figure S10. TGA plot of 3D–PATB under N_2 atmosphere.

S3.3 Photophysical and electrochemical characterization.

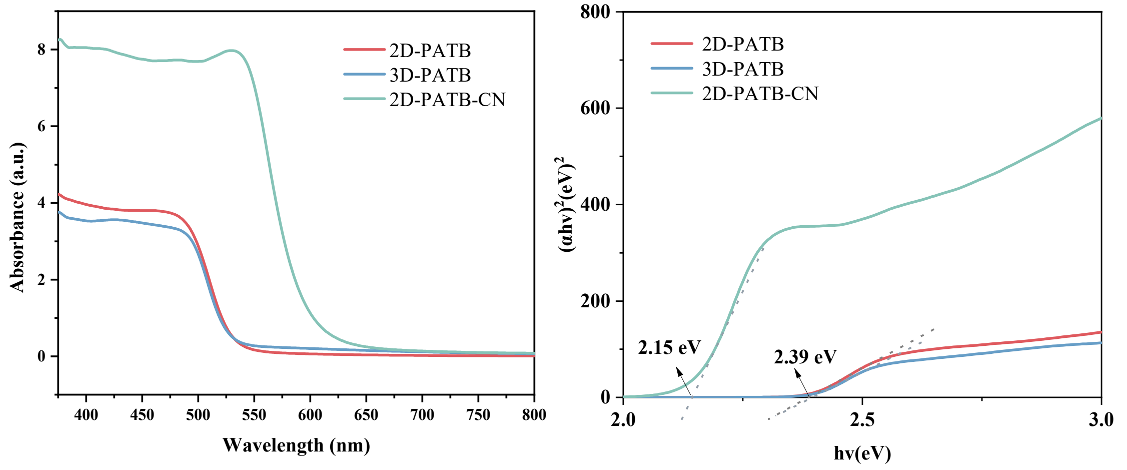

Figure S11. Diffuse reflectance UV-vis absorption spectra and Tauc plots of 2D-PATB, 3D-PATB and 2D-PATB-CN.

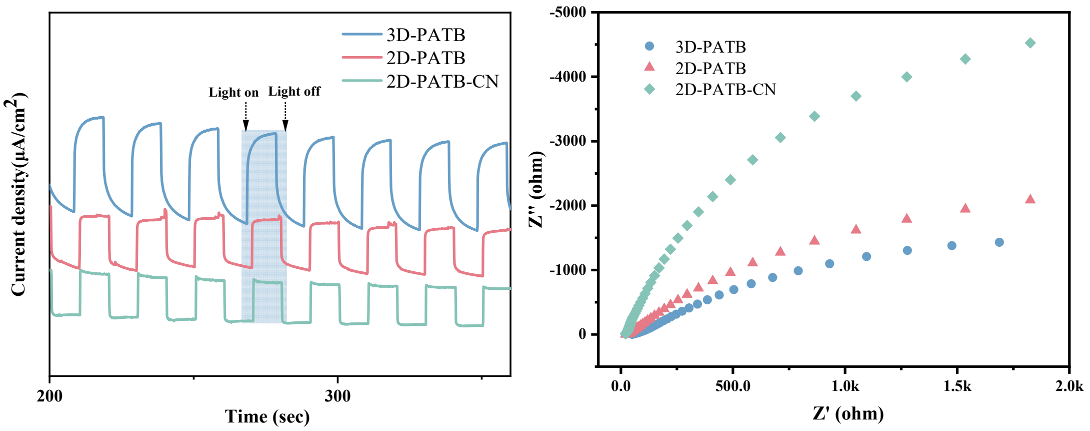

Figure S12. Photocurrent response curves and Nyquist plots of 2D-PATB, 3D-PATB and 2D-PATB-CN.

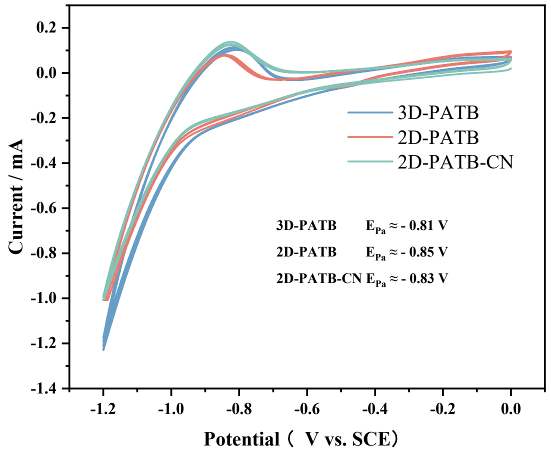

Figure S13. Cyclic voltammograms of 2D-PATB, 3D-PATB and 2D-PATB-CN recorded in 0.2 M Na_2SO_4 at a scan rate of 50 mV/s.

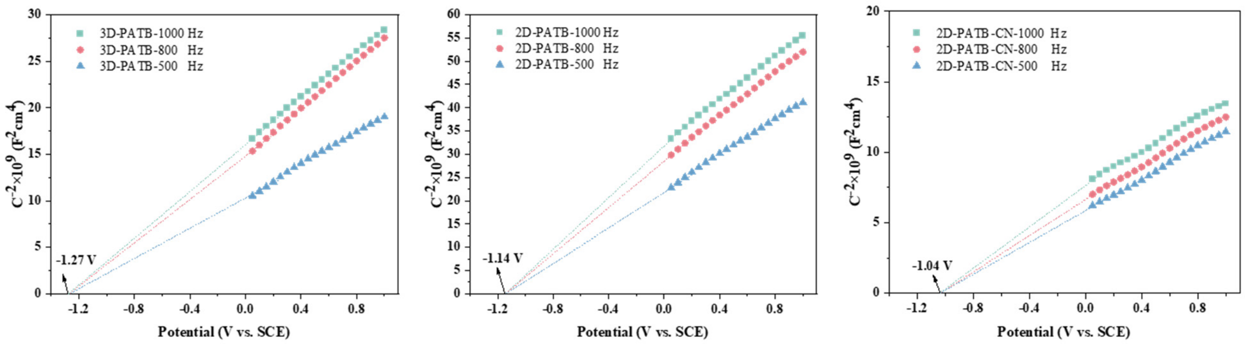

Figure S14. Mott-Schottky analysis of 2D-PATB, 3D-PATB and 2D-PATB-CN.

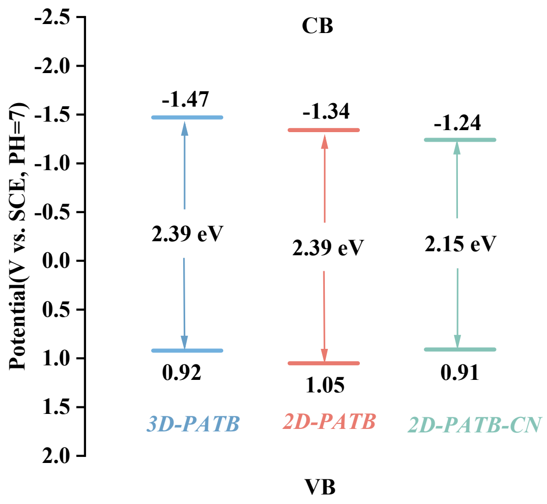

Figure S15. VB and CB positions of 2D-PATB, 3D-PATB and 2D-PATB-CN.

S3.4 Structure modeling and atomic coordinates of 2D–PATB–CN COF

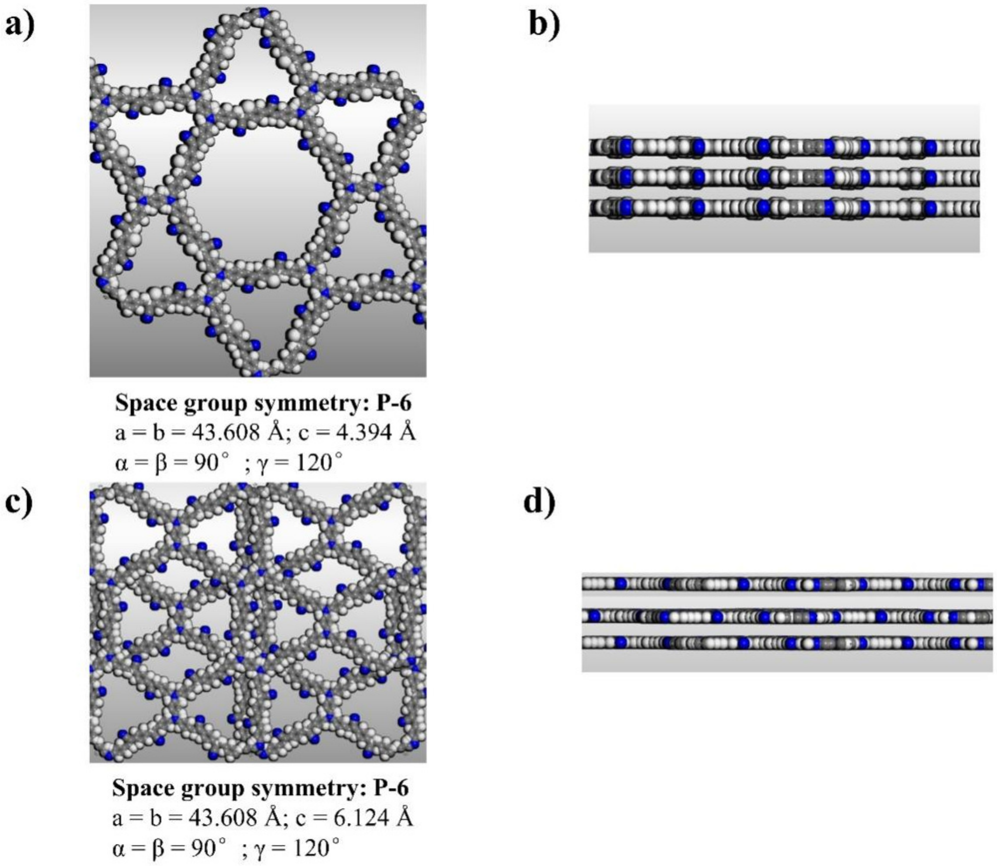

Figure S16 Computationally determined structure of 2D–PATB–CN. a) Theoretical structure of 2D-PATB-CN with eclipsed (AA) stacking arrangement. b) Side view of 2D–PATB–CN structure with eclipsed (AA) stacking arrangement. c) Theoretical structure of 2D–PATB–CN with staggered (AB) stacking arrangement. d) Side view of 2D–PATB–CN structure with staggered (AB) stacking arrangement.

Table S1. Fractional atomic coordinates for 2D–PATB–CN.

**2D–PATB–CN**<br>Space group symmetry: P-6 (168)<br>a = b = 43.608 Å; c = 4.394 Å<br>α = β = 90°; γ = 120°				
**Atom name**	**Atom**	**x**	**y**	**z**
C1	C	33.829	18.626	5.162
C2	C	33.221	19.883	5.166
C3	C	31.822	20.019	5.045
N4	N	31.192	21.324	4.807
C5	C	32.005	22.498	4.53
C6	C	29.771	21.413	4.504
C7	C	28.927	22.31	5.186
C8	C	27.567	22.384	4.885
C9	C	26.996	21.519	3.946
C10	C	27.824	20.619	3.259
C11	C	29.2	20.594	3.514
C12	C	33.011	22.456	3.548
C13	C	33.844	23.558	3.319
C14	C	33.621	24.763	3.995
C15	C	32.613	24.817	4.968
C16	C	31.83	23.696	5.245
C17	C	34.511	25.925	3.759
C18	C	25.546	21.631	3.679
C19	C	22.65	21.923	4.343
C20	C	21.274	22.146	4.326
C21	C	20.419	21.301	3.6
C22	C	20.976	20.23	2.878
C23	C	22.359	20.009	2.89
C24	C	23.213	20.857	3.618
C25	C	24.676	20.601	3.672
C26	C	18.957	21.553	3.623
C27	C	-3.497	18.227	3.84
N28	N	-3.857	19.318	3.976
N28	N	3.124	14.54	3.515
C29	C	3.484	13.443	3.428

N30	N	34.907	18.601	5.224
H31	H	33.87	20.744	5.233
H32	H	29.277	22.904	6.008
H33	H	26.944	23.081	5.433
H34	H	27.418	19.972	2.491
H35	H	29.823	19.916	2.956
H36	H	33.168	21.554	2.979
H37	H	34.632	23.484	2.58
H38	H	32.473	25.709	5.564
H39	H	31.162	23.762	6.082
H40	H	35.575	25.726	3.748
H41	H	25.161	22.641	3.6
H42	H	23.27	22.567	4.954
H43	H	20.872	22.963	4.913
H44	H	20.345	19.575	2.291
H45	H	22.765	19.182	2.319
H46	H	33.829	18.626	5.162

S3.5 Structure modeling and atomic coordinates of 2D–PATB COF

a)

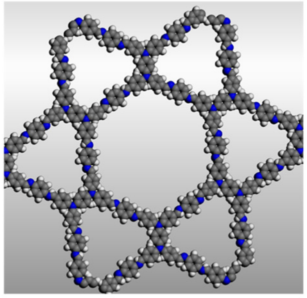

b)

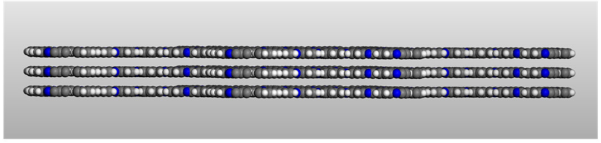

Space Symmetry Group: P6
a = b = 43.26 Å; c = 4.35 Å
α = β = 90.00°; γ = 120.00°

c)

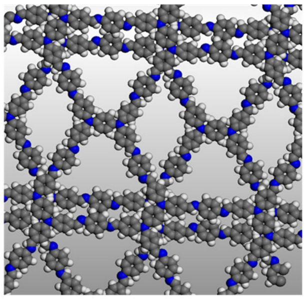

d)

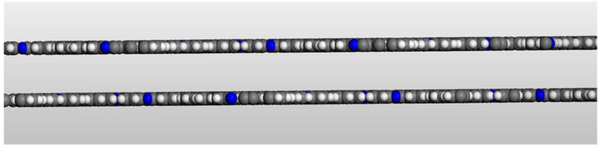

Space Symmetry Group: P1
a = 17.4 Å; b = c = 43.26 Å
α = 60.00°; β = γ = 90.00°

e)

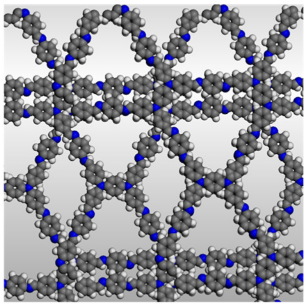

f)

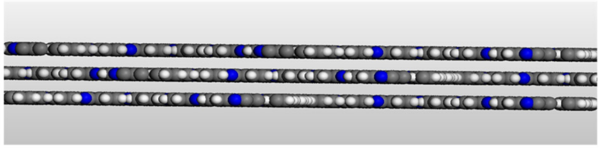

Space Symmetry Group: P1
a = 13.05 Å; b = c = 43.26 Å
α = 60.00°; β = γ = 90.00°

Figure S17 Computationally determined structure of 2D–PATB. a) Theoretical structure of 2D-PATB with eclipsed (AA) stacking arrangement. b) Side view of 2D–PATB structure with eclipsed (AA) stacking arrangement. c) Theoretical structure of 2D–PATB with staggered (AB) stacking arrangement. d) Side view of 2D–PATB structure with staggered (AB) stacking arrangement. e) Theoretical structure of 2D–PATB with staggered (ABC) stacking arrangement. f) Side view of 2D–PATB structure with staggered (ABC) stacking arrangement.

Table S2. Fractional atomic coordinates for 2D–PATB.

**2D–PATB**<br>Space group symmetry: P6<br>a = b = 43.26 Å; c = 4.35 Å<br>α = β = 90°; γ = 120°				
**Atom name**	**Atom**	**x**	**y**	**z**
C1	C	1.031	0.499	1.036
C2	C	1.033	0.531	1.005
C3	C	1.001	0.534	1.018
N4	N	1.002	0.569	1.010
C5	C	1.036	0.602	1.033
C6	C	0.970	0.571	0.965
C7	C	0.965	0.596	1.131
C8	C	0.934	0.598	1.093
C9	C	0.907	0.574	0.898
C10	C	0.912	0.550	0.722
C11	C	0.944	0.549	0.746
C12	C	1.068	0.603	0.945
C13	C	1.100	0.635	0.936
C14	C	1.103	0.667	1.034
C15	C	1.072	0.666	1.157
C16	C	1.039	0.634	1.151
C17	C	1.138	0.698	1.021
C18	C	0.875	0.576	0.876
C19	C	0.818	0.589	0.823
C20	C	0.790	0.596	0.813
C21	C	0.754	0.567	0.826
C22	C	0.748	0.532	0.846
C23	C	0.776	0.525	0.851
C24	C	0.812	0.554	0.843
N25	N	0.843	0.549	0.854
N26	N	0.723	0.574	0.818
N30	N	1.057	0.498	1.076
H26	H	1.059	0.557	0.968
H27	H	0.986	0.615	1.299
H28	H	0.931	0.619	1.222
H29	H	0.890	0.531	0.558
H30	H	0.949	0.531	0.588
H31	H	1.067	0.577	0.880
H32	H	1.125	0.635	0.849

H33	H	1.074	0.691	1.262
H34	H	1.015	0.634	1.244
H35	H	0.847	0.612	0.816
H36	H	0.719	0.509	0.859
H37	H	0.795	0.625	0.794
H38	H	0.771	0.497	0.862
H39	H	1.159	0.701	1.199
H40	H	0.877	0.604	0.878
H41	H	1.031	0.499	1.036
H42	H	1.033	0.531	1.005

S3.6 Atomic coordinates of 3D–PATB COF

Table S3. Fractional atomic coordinates for 3D–PATB.

**3D–PATB**<br>Space group symmetry: Pba2<br>a = 18.22 Å; b = 18.88 Å; c = 10.46 Å<br>α = β = γ = 90°				
**Atom name**	**Atom**	**x**	**y**	**z**
C1	C	0.959	0.498	0.749
C2	C	0.924	0.502	0.856
C3	C	0.958	0.502	0.976
N4	N	0.847	0.501	0.856
C5	C	0.836	0.399	0.719
C6	C	0.802	0.364	0.620
C7	C	0.742	0.394	0.561
C8	C	0.716	0.459	0.602
C9	C	0.751	0.494	0.701
C10	C	0.810	0.464	0.760
C11	C	0.809	0.531	0.958
C12	C	0.835	0.591	1.017
C13	C	0.807	0.613	1.133
C14	C	0.751	0.574	1.189
C15	C	0.725	0.514	1.130
C16	C	0.754	0.493	1.014
C17	C	0.729	0.588	1.321
N18	N	0.758	0.640	1.380
C19	C	0.731	0.653	1.500
C20	C	0.785	0.669	1.589
C21	C	0.766	0.680	1.714

C22	C	0.693	0.677	1.751
C23	C	0.640	0.661	1.662
C24	C	0.659	0.650	1.537
N25	N	0.674	0.695	1.878
C26	C	0.625	0.744	1.904
C27	C	0.718	0.365	0.443
N28	N	0.668	0.394	0.370
C29	C	0.653	0.360	0.255
C30	C	0.705	0.325	0.185
C31	C	0.688	0.297	0.067
C32	C	0.618	0.304	0.020
C33	C	0.565	0.339	0.090
C34	C	0.582	0.367	0.208
N35	N	0.597	0.262	-0.085
C36	C	0.585	0.298	-0.189
C37	C	0.557	0.260	-0.295
C38	C	0.537	0.190	-0.285
C39	C	0.506	0.188	-0.507
C40	C	0.552	0.294	-0.411
C41	C	0.495	0.075	-0.611
C42	C	0.436	0.028	-0.619
C43	C	0.444	-0.044	-0.617
C44	C	0.457	0.180	-0.722
C45	C	0.481	0.153	-0.837
C46	C	0.455	0.179	-0.950
C47	C	0.404	0.234	-0.949
C48	C	0.380	0.261	-0.835
C49	C	0.407	0.234	-0.722
N50	N	0.482	0.148	-0.610

S4. General procedures for the synthesis of substrates

S4.1 Synthesis of *N*-protected aminopyridinium salts

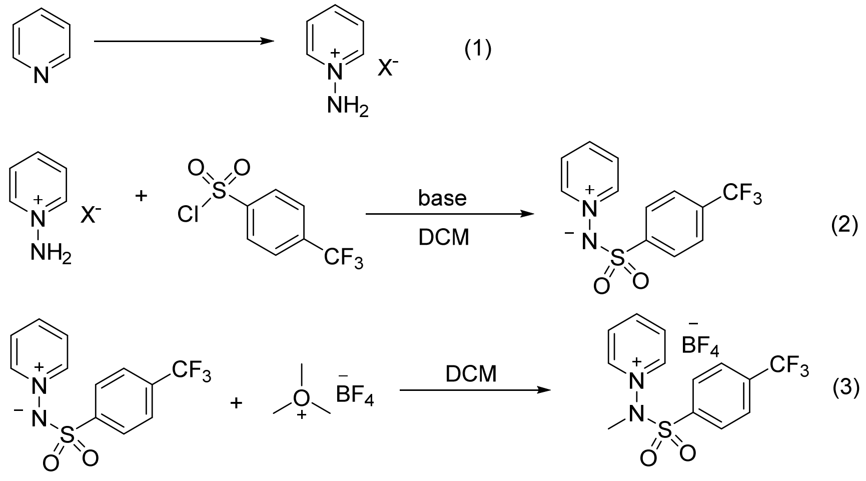

Figure S18. Synthesis of *N*-protected aminopyridinium salts.

(1) Synthesis of *N*-amino pyridinium salts from pyridines

Amination of pyridine was conducted using the previously developed method with hydroxylamine-*O*-sulfonic acid [5] or *O*-mesitylsulfonylhydroxylamine (MSH) [6].

(2) Synthesis of *N*-protected 1-aminopyridinium ylides

1-Aminopyridinium (1 equiv.) was dissolved in dichloromethane (0.2 M). Then, triethylamine (2.2 equiv.) and trifluoromethyl sulfonyl chloride (1.0 equiv.) were added at 0 °C. The reaction mixture was stirred at room temperature for 24 h. The resulting mixture was diluted with 1N NaOH and extracted with dichloromethane three times. The combined organic layers were dried over sodium sulphate, filtered, and concentrated in a vacuum. The resulting mixture was purified by flash column chromatography on silica gel (CH_2Cl_2: MeOH = 20: 1) to obtain *N*-protected 1-aminopyridinium ylides.

(3) Synthesis of *N*-protected aminopyridinium salts

N-protected 1-aminopyridinium ylide (1 equiv.) was dissolved in dichloromethane (0.1 M). Then, trimethyloxonium tetrafluoroborate (1.1 equiv.) was added at room temperature. The

reaction mixture was stirred at room temperature for 24 h. The resulting mixture was concentrated under reduced pressure. The product was recrystallized with diethyl ether at -20 °C. A white solid product was obtained.

S4.2 Synthesis of pyridinium salts

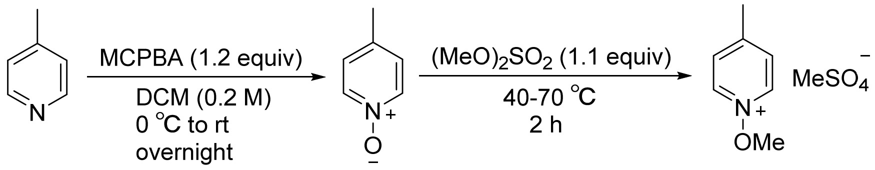

Figure S19. Synthesis of pyridinium salts.

General procedures for the synthesis of pyridinium salt:

Step 1: 4-Methylpyridine (1 equiv.) was dissolved in dichloromethane (0.2 M). The mixture was cooled to 0 °C and stirred for 15 min. A solution of *m*-chloroperbenzoic acid (MCPBA, 1.2 equiv.) in dichloromethane (5 mL) was added dropwise over 10 min. After completion, the result mixture was allowed to warm to room temperature and stirred overnight. the solvent was removed under reduced pressure, and the crude residue was purified by flash chromatography on silica gel (CH_2Cl_2: MeOH = 15: 1) to give the pyridine *N*-oxide.

Step 2: Pyridine *N*-oxide (1 equiv.) and dimethyl sulfate (1.1 equiv.). were added into a reaction tube. The mixture was warmed to 40-50 °C with stirring until fully homogenized, then heated to 70 °C and maintained for 2 h. After cooling, excess dimethyl sulfate was removed by rotary evaporation under high vacuum for12 h. The resulting pyridinium salt was obtained as a white solid and used directly in the next step without further purification.

S4.3 Synthesis of aryl silyl enol ethers

Sodium iodide (1 equiv.) and ketone precursor (1.2 equiv.) were placed in a flame-dried Schlenk tube under a nitrogen atmosphere. Anhydrous MeCN (1.7 M) was then added, and the suspension was stirred for 5 min. A solution of anhydrous triethylamine (1.5 equiv.) and trimethylchlorosilane

(1.2 equiv.) in MeCN were added dropwise. The reaction mixture was stirred for 13 hours at room temperature. The reaction was quenched with sat. aq. of NH_4Cl and the crude mixture was extracted three times with pentane. The organic layers were combined and washed sequentially with water and sat. aq. of NH_4Cl. After which the organic layers were dried with $MgSO_4$ and filtered. The solvent was evaporated under reduced pressure to give the desired silyl enol ether product.

S4.4 Synthesis of aryl sulfonium salts from simple arenes

Arene (1.0 equiv.) and *S*-oxide (1.1 equiv.) were dissolved in dry CH_2Cl_2 (0.1 M). Tf_2O (1.2 equiv.) was slowly added to the mixture under a nitrogen atmosphere at -78°C. The resulting solution was maintained at −78 °C for 15 min with vigorous stirring, then warmed to room temperature for one hour. The reaction was carefully quenched with methanol and the solvent was removed under vacuum, after which the sulfonium salt was precipitated by the addition of ice ether. If crystallization failed, purification was performed by silica gel chromatography (CH_2Cl_2: MeOH).

S4.5 Synthesis of aryl sulfonium salts from amide-containing or complex arenes

S-oxide (1.1 equiv.) was dissolved in dry CH_2Cl_2 (0.1 M). Tf_2O (1.2 equiv.) was slowly added to the solution under a nitrogen atmosphere at −78 °C. The resulting mixture was maintained at −78 °C for 1 hour with vigorous stirring. Amide-containing or complex arene (1.0 equiv.) was then added to the solution and stirring was continued at −78 °C for 15 min, then warmed to room temperature for one hour. The reaction was carefully quenched with methanol and the solvent was removed under vacuum, after which the sulfonium salt was precipitated by the addition of ice ether. If crystallization failed, purification was performed by silica gel chromatography (CH_2Cl_2: MeOH).

S5 Optimization of reaction conditions

Table S4. Optimization of reaction conditions for C4–site-selective C–H pyridylation of **2a** with cyclohexane **1**.

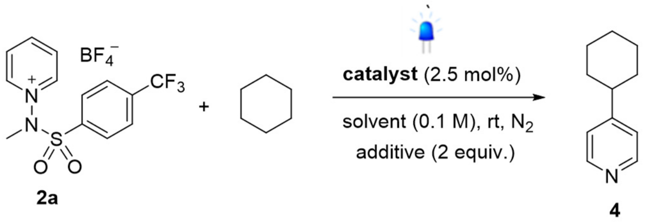

Entry	Catalyst	Solvent	Additive	Yield (%)[b]
1	2D–PATB	MeCN	-	66
2	3D–PATB	MeCN	-	82
3	2D–PATB–CN	MeCN	-	35
4	PATB–imine	MeCN	-	25
5	PATB	MeCN	-	48
6	BDA	MeCN	-	0
7	3D–PATB	DCE	-	60
8	Ir(ppy)$_3$	MeCN	-	50
9	4CzIPN	MeCN		60
10	Mes-Acr-BF$_4$	MeCN	-	7
11	3D–PATB	Toluene	-	trace
12	3D–PATB	DCM	-	61
13 [c]	3D–PATB	MeCN	-	0
14	-	MeCN	-	0
15	3D–PATB	MeCN	$NaHCO_3$ (2.0 equiv)	37

[a]Standard conditions: **2a** (0.1 mmol), cyclohexane **1** (1 mmol, 110 uL), additive (0.2 mmol) and catalyst (2.5 mol%) in solvent (1.0 mL) under blue LEDs in a N_2 atmosphere for 48 h. [b]Yields were determined by ^{1}H NMR with dibromomethane as an internal standard. [c] Reaction performed in dark.

Table S5. Optimization of reaction conditions for C2–site-selective C–H pyridylation of **3a** with THF.

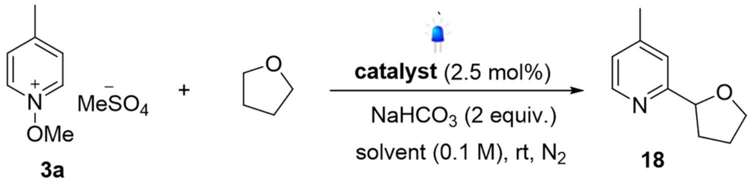

Entry	Catalyst	Solvent	Yield (%)[b]

1	2D–PATB	MeCN	43
2	3D–PATB	MeCN	92
3	2D–PATB-CN	MeCN	64
4	PATB–imine	MeCN	30
5 [c]	3D–PATB	MeCN	trace
6	-	MeCN	trace

[a]Standard conditions: **3a** (0.1 mmol), tetrahydrofuran (7.2 mmol), $NaHCO_3$ (0.2 mmol) and catalyst (2.5 mol%) in solvent (1.0 mL) under blue LEDs in a N_2 atmosphere for 24 h. [b]Yields were determined by ^1H NMR with dibromomethane as an internal standard. [c] Reaction performed without $NaHCO_3$.

Table S6. Optimization of reaction conditions for the formal C–H alkylation of **23a** with **24a**.

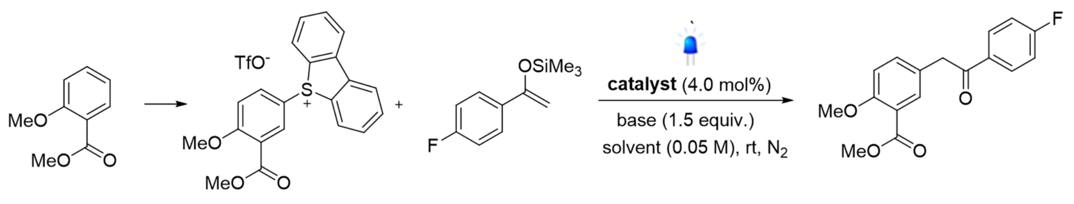

Entry	Catalyst	Solvent	Base	Yield (%)[b]
1	2D–PATB	MeCN	2,6-lutidine	46
2	3D–PATB	MeCN	2,6-lutidine	73
3	2D–PATB-CN	MeCN	2,6-lutidine	20
4	PATB–imine	MeCN	2,6-lutidine	57
5	3D–PATB	DCE	2,6-lutidine	51
6	3D–PATB	DMF	2,6-lutidine	27
7	3D–PATB	DCM	2,6-lutidine	45
8	3D–PATB	THF	2,6-lutidine	16
9[c]	3D–PATB	MeCN	2,6-lutidine	0
10	-	MeCN	2,6-lutidine	27
11	3D–PATB	MeCN	K_2CO_3 (1.5 equiv.)	trace
12	3D–PATB	MeCN	DMAP (1.5 equiv.)	trace
13	3D–PATB	MeCN	CH_3COONa (1.5 equiv.)	53

[a]Standard conditions: triarylsulfonium salt **23a** (0.05 mmol), silyl enol ether **24a** (0.25 mmol), base (0.075 mmol) and catalyst (4.0 mol%) in solvent (1.0 mL) under blue LEDs in a N_2 atmosphere for 48 h. [b]Yields were determined by ^1H NMR with dibromomethane as an internal standard. [c]Reaction performed in dark.

Table S7. Optimization of reaction conditions for the [3+2] cyclization of **35a** with **36d**.

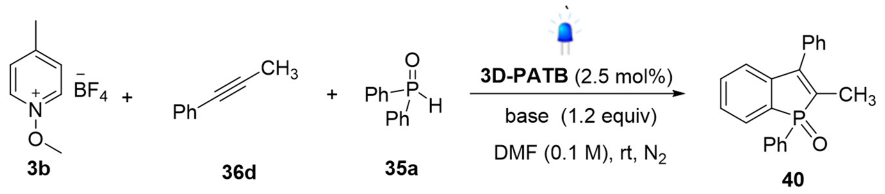

Entry	Catalyst	Base	Yield (%)[b]
1	3D–PATB	K_2HPO_4	69
2	3D–PATB	CH_3COONa	46
3	3D–PATB	Na_2CO_3	23
4	3D–PATB	$NaHCO_3$	40
5	3D–PATB	2,6-lutidine	47
6	2D-PATB	K_2HPO_4	37
7	2D-PATB-CN	K_2HPO_4	25
8	-	K_2HPO_4	trace

[a]Standard conditions: pyridinium salt **3b** (0.2 mmol), 1-phenylpropyne **36d** (0.1 mmol), diphenylphosphine oxide **35a** (0.2 mmol), K_2HPO_4 (0.12 mmol) and catalyst (2.5 mol%) in solvent (1.0 mL) under blue LEDs in a N_2 atmosphere for 48 h. [b]Yields were determined by ^1H NMR with dibromomethane as an internal standard.

S6 General procedure and product characterization

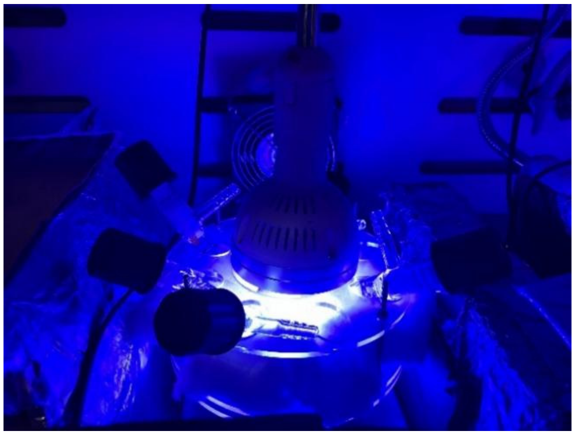

Figure S20. General reaction setup.

S6.1 General procedure and product characterization for C4–site-selective C–H pyridylation

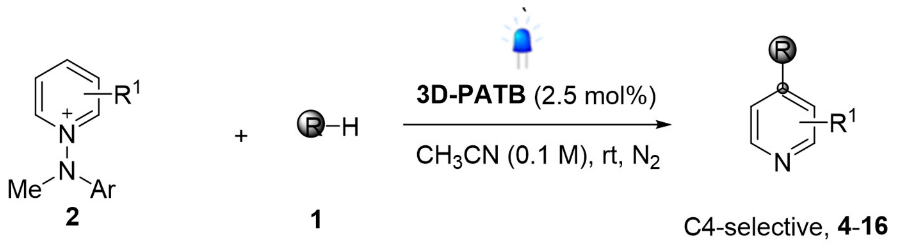

N-aminopyridinium salt **2** (0.1 mmol), 3D–PATB (2.5 mol % catalyst loading based on the PATB linker) and hydrocarbon **1** (1 mmol) were mixed in anhydrous acetonitrile (MeCN, 1.0 mL) in a sealed test tube. The resulting mixture was stirred under blue LED irradiation (440-450 nm) at room temperature under a nitrogen atmosphere for 48 hours. After that, 3D–PATB was filtered off, the solvent was removed under vacuum, and the residue was purified by column chromatography on silica gel to give products **4–16**.

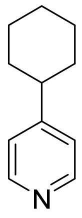

4-Cyclohexylpyridine (4). Yield 82%, 13.2 mg, pale yellow oil. ^{1}H NMR (400 MHz, $CDCl_3$) δ 8.47 (d, *J* = 6.0 Hz, 2H), 7.10 (d, *J* = 6.0 Hz, 2H), 2.48 (m, 1H), 1.92 – 1.80 (comp, 4H), 1.78 – 1.70 (m, 1H), 1.44 – 1.36 (comp, 4H), 1.30 – 1.19 (m, 1H). Characterization data matched that reported in the literature [1].

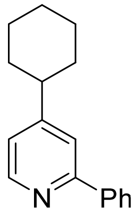

4-Cyclohexyl-2-phenylpyridine (6). Yield 93%, 22.1 mg, colorless oil. ^{1}H NMR (400 MHz, $CDCl_3$) δ 8.57 (d, *J* = 4.8 Hz, 1H), 7.98 – 7.96 (comp, 2H), 7.56 (s, 1H), 7.48 – 7.44 (comp, 2H), 7.42 – 7.40 (m, 1H), 7.08 (d, *J* = 4.8 Hz, 1H), 2.65 – 2.49 (m, 1H), 1.97 – 1.85 (comp, 4H), 1.80 – 1.75 (m, 1H), 1.54 – 1.39 (comp, 4H), 1.39 – 1.30 (m, 1H). Characterization data matched that reported in the literature [1].

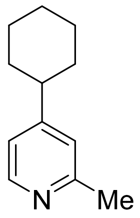

4-Cyclohexyl-2-methylpyridine (7). Yield 78%, 13.7 mg, pale yellow oil. ^{1}H NMR (400 MHz, $CDCl_3$) δ 8.36 (d, *J* = 5.2 Hz, 1H), 6.98 (s, 1H), 6.93 (d, *J* = 5.2 Hz, 1H), 2.51 (s, 3H), 2.48 – 2.39 (m, 1H), 1.90 – 1.79 (comp, 4H), 1.78 – 1.72 (m, 1H), 1.46 – 1.34 (comp, 4H), 1.28 – 1.23 (m, 1H). Characterization data matched that reported in the literature [1].

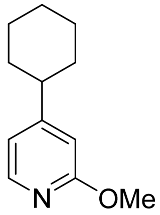

4-Cyclohexyl-2-methoxypyridine (8). Yield 60%, 11.5 mg, pale yellow Oil. ^{1}H NMR (400 MHz, $CDCl_3$) δ 8.04 (d, *J* = 5.2 Hz, 1H), 6.72 (d, *J* = 5.2 Hz, 1H), 6.56 (s, 1H), 3.91 (s, 3H), 2.52 – 2.39 (m, 1H), 1.92 – 1.80 (comp, 4H), 1.77 – 1.70 (m, 1H), 1.44 – 1.32 (comp, 4H), 1.29 – 1.22 (m, 2H). Characterization data matched that reported in the literature [1].

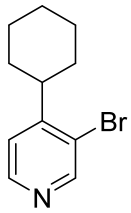

3-Bromo-4-cyclohexylpyridine (9). Yield 70%, 16.8 mg, pale yellow oil. ^{1}H NMR (400 MHz, $CDCl_3$) δ 8.79 (s, 1H), 8.68 (d, *J* = 5.2 Hz, 1H), 7.36 (d, *J* = 5.2 Hz, 1H), 2.93 – 2.88 (m, 1H), 1.90 – 1.79 (comp, 4H), 1.71 – 1.66 (m, 1H), 1.49 – 1.36 (comp, 4H), 1.33 – 1.24 (m, 1H). Characterization data matched that reported in the literature [1].

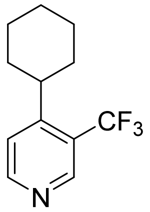

4-Cyclohexyl-3-(trifluoromethyl)pyridine (10). Yield 90%, 20.6 mg, pale yellow oil. ^{1}H NMR (400 MHz, $CDCl_3$) δ 8.64 (s, 1H), 8.41 (d, *J* = 4.8 Hz, 1H), 7.16 (d, *J* = 4.8 Hz, 1H), 3.01 – 2.82 (m, 1H), 1.92 – 1.80 (comp, 4H), 1.80 – 1.72 (m, 1H), 1.50 – 1.30 (comp, 4H), 1.29 – 1.20 (m, 1H). Characterization data matched that reported in the literature [1].

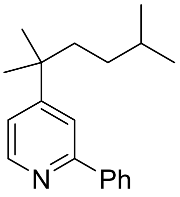

4-(2,5-Dimethylhexan-2-yl)-2-phenylpyridine (11). Yield 87%, 23.2 mg, colorless oil. ^{1}H NMR (400 MHz, $CDCl_3$) δ 8.60 (d, *J* = 5.2 Hz, 1H), 7.99 – 7.95 (m, 2H), 7.65 (d, *J* = 1.6 Hz, 1H), 7.50 – 7.46 (m, 2H), 7.44 – 7.39 (m, 1H), 7.18 (dd, *J* = 5.2, 1.6 Hz, 1H), 1.67 – 1.63 (m, 2H), 1.46 – 1.40 (m, 1H), 1.33 (comp, 6H), 0.99 – 0.94 (m, 2H), 0.82 (comp, *J* = 6.6 Hz, 6H). Characterization data matched that reported in the literature [1].

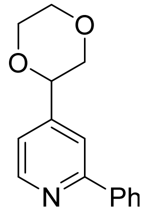

4-(1,4-Dioxan-2-yl)-2-phenylpyridine (12). Yield 70%, 22.1 mg, pale yellow oil. ^{1}H NMR (400 MHz, $CDCl_3$) δ 8.66 (d, J = 5.2 Hz, 1H), 8.00 (d, *J* = 7.6 Hz, 2H), 7.72 (s, 1H), 7.47 (t, *J* = 7.6 Hz, 2H), 7.42 (d, *J* = 7.6 Hz, 1H), 7.19 (d, *J* = 5.2 Hz, 1H), 4.70 (dd, *J* = 10.0, 2.8 Hz, 1H), 4.03 – 3.88 (comp, 3H), 3.84 – 3.81 (m, 1H), 3.77 – 3.70 (m, 1H), 3.44 (t, *J* = 10.8 Hz, 1H). Characterization data matched that reported in the literature [1].

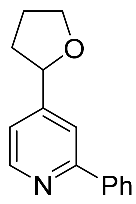

2-Phenyl-4-(tetrahydrofuran-2-yl)pyridine (13). One hundred equivalents of tetrahydrofuran (THF) were used as substrate. Yield 60%, 13.5 mg, pale yellow oil. ^{1}H NMR (400 MHz, $CDCl_3$) δ 8.63 (d, *J* = 5.2 Hz, 1H), 8.00 (d, *J* =7.2 Hz, 2H), 7.70 (s, 1H), 7.51 – 7.44 (m, 2H), 7.44 – 7.38 (m, 1H), 7.18 (d, *J* = 4.8, 1.6 Hz, 1H), 4.97 (t, *J* = 6.8 Hz, 1H), 4.20 – 4.07 (m, 1H), 4.05 – 3.93 (m, 1H), 2.48 – 2.35 (m, 1H), 2.07 – 1.95 (m, 2H), 1.87 – 1.79 (m, 1H). Characterization data matched that reported in the literature [1].

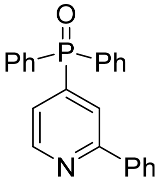

Diphenyl(2-phenylpyridin-4-yl)phosphine oxide (14). Yield 50%, 17.8 mg, white solid. ^{1}H NMR (400 MHz, $CDCl_3$) δ 8.80 (t, *J* = 4.8 Hz, 1H), 8.08 (d, *J* = 12.8 Hz, 1H), 7.97 (dd, *J* = 8.0, 1.6 Hz, 2H), 7.73 – 7.68 (comp, 4H), 7.63 – 7.57 (m, 2H), 7.54 – 7.49 (comp, 4H), 7.48 – 7.42 (m, 3H), 7.40 – 7.36 (m, 1H). Characterization data matched that reported in the literature [1].

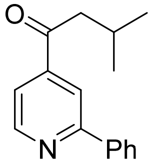

3-Methyl-1-(2-phenylpyridin-4-yl)butan-1-one (15). Two equivalents of sodium acetate (NaOAc) were used as a base. Yield 90%, 21.5 mg, colorless oil. ^{1}H NMR (400 MHz, $CDCl_3$) δ 8.86 (d, *J* = 4.8 Hz, 1H), 8.15 (s, 1H), 8.04 (d, *J* = 7.6 Hz, 2H), 7.64 (d, *J* = 5.2 Hz, 1H), 7.54 – 7.48 (m, 2H), 7.48 – 7.42 (m, 1H), 2.89 (d, *J* = 6.8 Hz, 2H), 2.38 – 2.27 (m, 1H), 1.03 (s, 3H), 1.02 (s, 3H). Characterization data matched that reported in the literature [1].

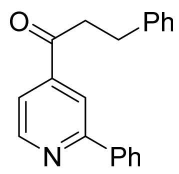

3-Phenyl-1-(2-phenylpyridin-4-yl)propan-1-one (16). 2 equiv of NaOAc used as a base. Yield 60%, 17.2 mg, colorless oil. ^{1}H NMR (400 MHz, $CDCl_3$) δ 8.85 (d, *J* = 4.8 Hz, 1H), 8.14 (s, 1H), 8.02 (d, *J* = 6.8 Hz, 2H), 7.63 (dd, *J* = 5.2, 1.6 Hz, 1H), 7.54 – 7.42 (comp, 3H), 7.35 – 7.29 (m, 2H), 7.28 – 7.24 (comp, 3H), 3.35 (t, *J* = 7.6 Hz, 2H), 3.11 (t, *J* = 7.6 Hz, 2H). Characterization data matched that reported in the literature [1].

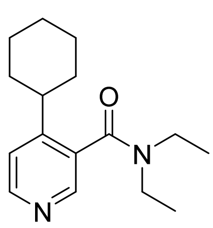

4-Cyclohexyl-*N*,*N*-diethylnicotinamide (48). Yield 80%, 20.8 mg, colorless oil. ^{1}H NMR (400 MHz, $CDCl_3$) δ 8.52 (s, 1H), 8.37 (s, 1H), 7.22 (d, *J* = 5.2 Hz, 1H), 3.89 – 3.84 (m, 1H), 3.33 – 3.28 (m, 1H), 3.12 (q, *J* = 7.2 Hz, 2H), 2.60 – 2.52 (m, 1H), 2.02 – 1.92 (m, 1H), 1.89 – 1.78 (comp, 3H), 1.75 – 1.68 (m, 2H), 1.37 – 1.30 (m, 2H), 1.26 (t, *J* = 7.2 Hz, 6H), 1.07 (t, *J* = 7.2 Hz, 3H). 13C NMR (101 MHz, $CDCl_3$) δ 168.3, 153.1, 150.1, 146.3, 121.7, 77.5, 77.2, 76.8, 43.1, 41.0, 38.9, 26.0, 14.1, 12.8. HRMS (ESI–TOF) calcd for $C_{16}H_{25}N_2O^+$ ([M+H$^+$]) = 261.1967, Found 261.1959.

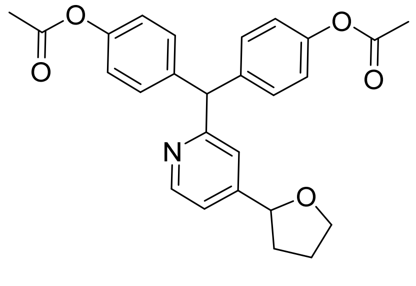

((4-(Tetrahydrofuran-2-yl)pyridin-2-yl)methylene e)bis(4,1-phenylene) diacetate (50). Yield 60%, 25.9 mg, pale yellow oil. ^{1}H NMR (400 MHz, $CDCl_3$) δ 8.52 (d, *J* = 5.2 Hz, 1H), 7.22 – 7.15 (comp, 4H), 7.11 (d, *J* = 5.2 Hz, 1H), 7.08 (s, 1H), 7.04 – 6.97 (comp, 4H), 5.62 (s, 1H), 4.83 (t, *J* = 7.2 Hz, 1H), 4.10 – 3.97 (m, 1H), 3.97 – 3.85 (m, 1H), 2.38 – 2.29 (m, 1H), 2.27 (comp, 6H), 2.01 – 1.90 (m, 2H), 1.76 – 1.65 (m, 1H). ^{13}C NMR (101 MHz, $CDCl_3$) δ 169.5, 162.5, 153.6, 149.6, 149.3, 140.1, 140.0, 130.3, 121.5, 121.4, 120.6, 118.6, 79.2, 77.4, 77.1, 76.8, 68.9, 58.0, 34.3, 25.8, 21.2. HRMS (ESI-TOF) calcd for $C_{26}H_{26}NO_5^+$ ([M+H$^+$]) = 432.1811, Found 432.1806.

S6.2 General procedure and product characterization for C2–site-selective C–H pyridylation

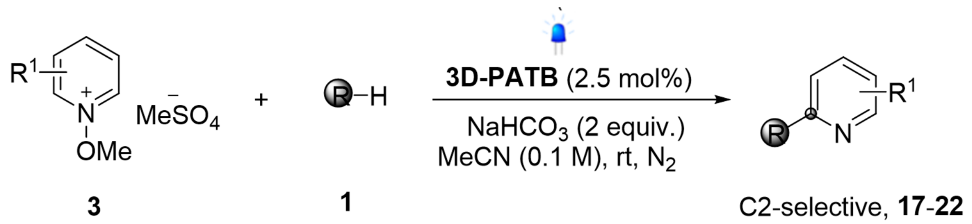

N-Methoxy pyridinium methylsulfate **3a** (0.1 mmol), 3D–PATB (2.5 mol % catalyst loading based on the PATB linker), hydrocarbon **1** (7.2 mmol) and $NaHCO_3$ (0.2 mmol) were mixed in anhydrous acetonitrile (MeCN, 1.0 mL) in a sealed test tube. The resulting mixture was stirred under blue LED irradiation (440-450 nm) at room temperature in a nitrogen atmosphere for 24 hours. After that, 3D–PATB was filtered off, the solvent was removed under vacuum, and the residue was purified by column chromatography on silica gel to give products **17-22**.

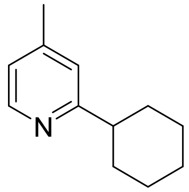

2-Cyclohexyl-4-methylpyridine (17). Yield 64%, 11.2 mg, colorless oil. ^{1}H NMR (400 MHz, $CDCl_3$) δ 8.37 (d, *J* = 5.2 Hz, 1H), 6.96 (s, 1H), 6.91 (d, *J* = 5.2 Hz, 1H), 2.68 – 2.60 (m, 1H), 2.31 (s, 3H), 1.94 – 1.90 (m, 2H), 1.86 – 1.81 (m, 2H), 1.76 – 1.70 (m, 1H), 1.56 – 1.32 (comp, 5H). Characterization data matched that reported in the literature [2].

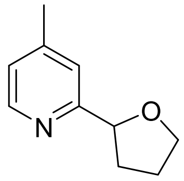

4-Methyl-2-(tetrahydrofuran-2-yl)pyridine (18). Yield 92%, 15.0 mg, colorless oil. ^{1}H NMR (400 MHz, $CDCl_3$) δ 8.37 (d, *J* = 5.6 Hz, 1H), 7.25 (s, 1H), 6.96 (d, *J* = 5.6 Hz, 1H), 4.97 (t, *J* = 6.8 Hz, 1H), 4.16 – 4.05 (m, 1H), 4.03 – 3.91 (m, 1H), 2.44 – 2.36 (m, 1H), 2.33 (s, 3H), 2.01 – 1.92 (comp, 3H). Characterization data matched that reported in the literature [2].

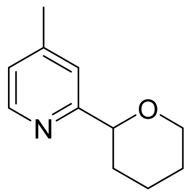

4-Methyl-2-(tetrahydro-2H-pyran-2-yl)pyridine (19). Yield 73%, 12.9 mg, colorless oil. ^{1}H NMR (400 MHz, $CDCl_3$) δ 8.32 (d, *J* = 5.2 Hz, 1H), 7.20 (s, 1H), 6.92 (d, *J* = 5.2 Hz, 1H), 4.36 (dd, *J* = 11.2, 2.4 Hz, 1H), 4.10 (dd, *J* = 11.2, 4.4 Hz, 1H), 3.63 – 3.50 (m, 1H), 2.28 (s, 3H), 2.01 – 1.94 (m, 1H), 1.91 – 1.83 (m, 1H), 1.67 – 1.47 (comp, 4H). Characterization data matched that reported in the literature [2].

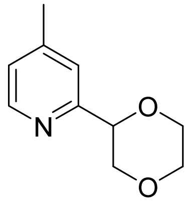

2-(1,4-Dioxan-2-yl)-4-methylpyridine (20). Yield 89%, 16.0 mg, colorless oil. ^{1}H NMR (400 MHz, $CDCl_3$) δ 8.40 (d, *J* = 5.2 Hz, 1H), 7.28 (s, 1H), 7.02 (d, *J* = 5.2 Hz, 1H), 4.71 (dd, *J* = 10.0, 2.8 Hz, 1H), 4.13 (dd, *J* = 11.2, 2.8 Hz, 1H), 4.01 – 3.89 (m, 2H), 3.86 – 3.67 (m, 2H), 3.63 – 3.45 (m, 1H), 2.36 (s, 3H). Characterization data matched that reported in the literature [2].

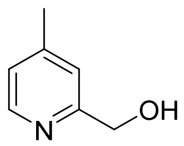

(4-Methylpyridin-2-yl)methanol (21). Yield 45%, 5.5 mg, colorless oil. ^{1}H NMR (400 MHz, $CDCl_3$) δ 8.39 (d, *J* = 5.2 Hz, 1H), 7.07 (s, 1H), 7.02 (d, *J* = 5.2 Hz, 1H), 4.71 (s, 2H), 2.35 (s, 3H). Characterization data matched that reported in the literature [2].

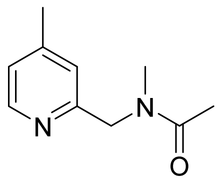

***N*-Methyl-*N*-((4-methylpyridin-2-yl)methyl)acetamide (22)**. Yield 70%, 12.5 mg, colorless oil. ^{1}H NMR (400 MHz, $CDCl_3$) δ 8.38 (dd, *J* = 21.8, 5.2 Hz, 1H), 7.09 – 6.91 (m, 2H), 4.61 (d, *J* = 30.4 Hz, 2H), 3.01 (d, *J* = 17.6 Hz, 3H), 2.33 (d, *J* = 13.9 Hz, 3H), 2.14 (d, *J* = 11.0 Hz, 3H). Characterization data matched that reported in the literature [2].

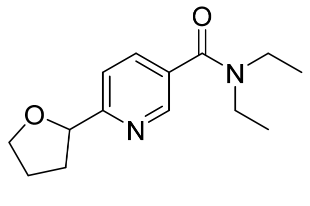

***N*, *N*-Diethyl-6-(tetrahydrofuran-2-yl)nicotinamide (47)**. Yield 48%, 11.9 mg, colorless oil. ^{1}H NMR (400 MHz, $CDCl_3$) δ 8.55 (d, *J* = 2.4 Hz, 1H), 7.69 (dd, *J* = 8.0, 2.4 Hz, 1H), 7.47 (d, *J* = 8.0 Hz, 1H), 5.01 (t, *J* = 7.2 Hz, 1H), 4.14 – 4.04 (m, 1H), 3.99 – 3.94 (m 1H), 3.60 – 3.50 (m,

2H), 3.33 – 3.20 (m, 2H), 2.49 – 2.35 (m, 1H), 2.03 – 1.90 (m, 4H), 1.32 – 1.11 (comp, 6H). Characterization data matched that reported in the literature [2].

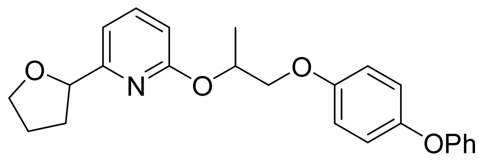

2-((1-(4-Phenoxyphenoxy)propan-2-yl)oxy)-6-(tetrahydrofuran-2-yl)pyridine (49). Yield 50%, 19.6 mg, colorless oil. 1H NMR (400 MHz, CDCl3) δ 7.54 (t, J = 8.0 Hz, 1H), 7.33 – 7.25 (m, 2H), 7.04 (t, J = 7.6 Hz, 1H), 7.02 – 6.89 (comp, 7H), 6.60 (d, J = 8.0 Hz, 1H), 5.60 – 5.54 (m, 1H), 5.01 – 4.87 (m, 1H), 4.22 (dd, J = 10.0, 5.2 Hz, 1H), 4.12 – 4.00 (m, 2H), 3.98 – 3.92 (m, 1H), 2.41 – 2.25 (m, 1H), 2.10 – 1.90 (m, 3H), 1.48 (d, J = 6.4 Hz, 3H). Characterization data matched that reported in the literature [2].

S6.3 General procedure and product characterization for formal C–H alkylation

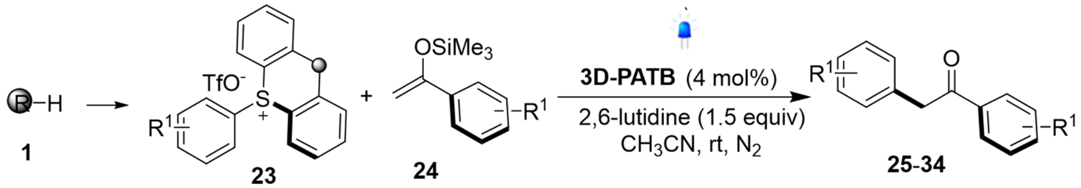

Aryl sulfonium salts **23** (0.05 mmol), silyl enol ethers **24** (0.25 mmol), 2,6-lutidine (0.075 mmol) and 3D–PATB (4.0 mol% catalyst loading based on the PATB linker) were mixed in anhydrous acetonitrile (MeCN, 1.0 mL) in a sealed test tube. The resulting mixture was stirred under blue LED irradiation (390-400 nm) at room temperature in a nitrogen atmosphere for 48 hours. After that, the 3D–PATB was filtered off, the solvent was removed under vacuum, and the residue was purified by column chromatography on silica gel to afford products **25–34**.

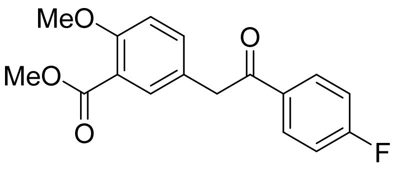

Methyl-5-(2-(4-fluorophenyl)-2-oxoethyl)-2-methoxybenzoate (25). Yield 73%, 11.0 mg, yellow crystalline solid. ^{1}H NMR (400 MHz, CDCl$_3$) δ 8.05 – 7.99 (comp, 2H), 7.70 (d, *J* = 2.4 Hz, 1H), 7.36 (dd, *J* = 8.4, 2.4 Hz, 1H), 7.16 – 7.10 (comp, 2H), 6.95 (d, *J* = 8.4 Hz, 1H), 4.22 (s, 2H), 3.89 (s, 3H), 3.87 (s, 3H). Characterization data matched that reported in the literature [3].

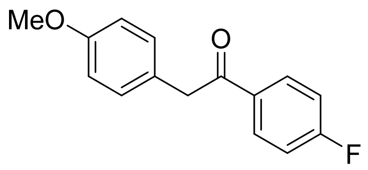

1-(4-Fluorophenyl)-2-(4-methoxyphenyl)ethan-1-one (26). Yield 68%, 8.3 mg, white solid. ^{1}H NMR (400 MHz, CDCl$_3$) δ 8.05 – 7.99 (comp, 2H), 7.33 (dd, *J* = 8.8, 2.0 Hz, 1H), 7.26 (d, *J* = 2.8 Hz, 1H), 7.24 (d, *J* = 1.2 Hz, 1H), 7.16 – 7.07 (comp, 2H), 6.74 (d, *J* = 8.4 Hz, 1H), 4.18 (s, 2H), 3.73 (s, 3H). Characterization data matched that reported in the literature [3].

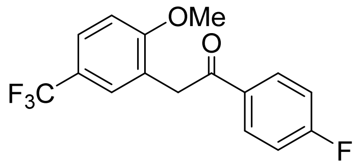

1-(4-Fluorophenyl)-2-(2-methoxy-5-(trifluoromethy l)phenyl)ethan-1-one (27). Phenoxathiinium (PXT$^+$) salt was used to prepare the compound. Yield 46%, 7.2 mg, yellow amorphous solid. ^{1}H NMR (400 MHz, CDCl$_3$) δ 8.19 – 7.95 (comp, 2H), 7.55 (dd, *J* = 8.8, 2.4 Hz, 1H), 7.43 (d, *J* = 2.4 Hz, 1H), 7.19 – 7.12 (comp, 2H), 6.95 (d, *J* = 8.8 Hz, 1H), 4.28 (s, 2H), 3.82 (s, 3H). Characterization data matched that reported in the literature [3].

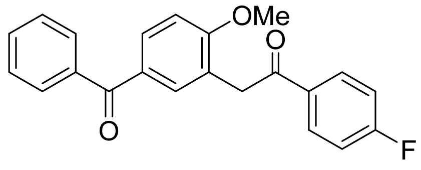

2-(5-Benzoyl-2-methoxyphenyl)-1-(4-fluorophen yl)ethan-1-one (28). Yield 88%, 15.3 mg, colorless amorphous solid. ^{1}H NMR (400 MHz, CDCl$_3$) δ 8.09 – 8.03 (comp, 2H), 7.81 (dd, *J* = 8.8, 2.2 Hz, 1H), 7.78 - 7.68 (comp, 3H), 7.59 – 7.52 (m, 1H), 7.47 – 7.44 (t, *J* = 7.6 Hz, 2H), 7.17 – 7.13 (comp, 2H), 6.95 (d, *J* = 8.8 Hz, 1H), 4.29 (s, 2H), 3.86 (s, 3H). Characterization data matched that reported in the literature [3].

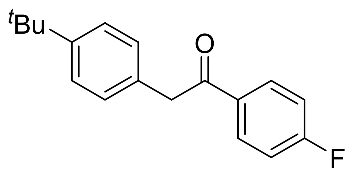

2-(4-(Tert-butyl)phenyl)-1-(4-fluorophenyl)ethan-1-on e (29). Yield 62%, 8.4 mg, pale yellow solid. ^{1}H NMR (400 MHz, CDCl$_3$) δ 8.08 – 8.01 (comp, 2H), 7.37 – 7.32 (comp, 2H), 7.21 – 7.16 (comp, 2H), 7.15 – 7.10 (comp, 2H), 4.22 (s, 2H), 1.30 (s, 9H). Characterization data matched that reported in the literature [3].

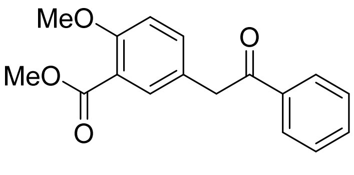

Methyl-2-methoxy-5-(2-oxo-2-phenylethyl)benzoate (30). Yield 53%, 8.7 mg, brown amorphous solid. ^{1}H NMR (400 MHz, $CDCl_3$) δ 8.02 – 7.97 (comp, 2H), 7.71 (d, *J* = 2.4 Hz, 1H), 7.57 (t, *J* = 7.2 Hz, 1H), 7.49 – 7.45 (comp, 2H), 7.37 (dd, *J* = 8.8, 2.4 Hz, 1H), 6.95 (d, *J* = 8.8 Hz, 1H), 4.25 (s, 2H), 3.89 (s, 3H), 3.87 (s, 3H). Characterization data matched that reported in the literature [3].

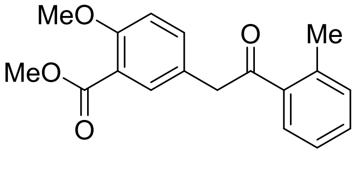

Methyl-2-methoxy-5-(2-oxo-2-(o-tolyl)ethyl)benzoate (31). Yield 47%, 7.0 mg, brown amorphous solid. ^{1}H NMR (400 MHz, $CDCl_3$) δ 7.71 (d, *J* = 6.4 Hz, 1H), 7.68 (d, *J* = 2.4 Hz, 1H), 7.41 – 7.32 (comp, 2H), 7.29 – 7.23 (comp, 3H), 6.95 (d, *J* = 8.4 Hz, 1H), 4.17 (s, 2H), 3.89 (s, 3H), 3.87 (s, 3H), 2.45 (s, 3H). Characterization data matched that reported in the literature [3].

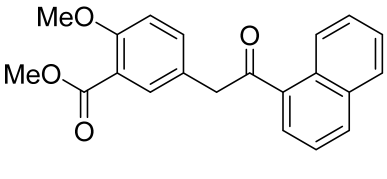

Methyl-2-methoxy-5-(2-(naphthalen-1-yl)-2-oxoethyl)benzoate (32). Yield 48%, 8.0 mg, brown amorphous solid. ^{1}H NMR (400 MHz, $CDCl_3$) δ 8.54 (s, 1H), 8.05 (d, *J* = 8.0 Hz, 1H), 7.97 (d, *J* = 8.0 Hz , 1H), 7.91 – 7.85 (comp, 2H), 7.78 – 7.75 (m, 1H), 7.62 – 7.54 (comp, 2H), 7.44 – 7.38 (comp, 1H), 6.96 (d, *J* = 8.8 Hz, 1H), 4.39 (s, 2H), 3.89 (s, 3H), 3.88 (s, 3H). Characterization data matched that reported in the literature [3].

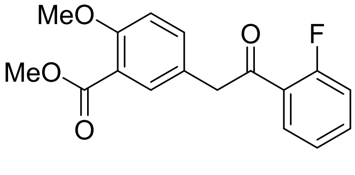

Methyl-5-(2-(2-fluorophenyl)-2-oxoethyl)-2-methox y benzoate (33). Yield 60%, 9.1 mg, pale orange amorphous solid. ^{1}H NMR (400 MHz, $CDCl_3$) δ 7.89 - 7.82 (m, 1H), 7.69 (d, *J* = 2.4 Hz, 1H), 7.56 – 7.48 (m, 1H), 7.36 (dd, *J* = 8.8, 2.4 Hz, 1H), 7.24 – 7.20 (m, 1H), 7.17 – 7.11 (m, 1H), 6.95 (d, *J* = 8.8 Hz, 1H), 4.25 (d, *J* = 2.8 Hz, 2H), 3.89 (s, 3H), 3.87 (s, 3H). Characterization data matched that reported in the literature [3].

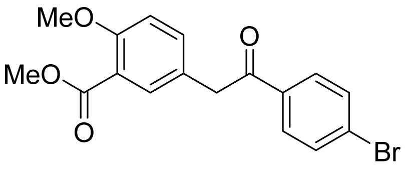

Methyl-5-(2-(4-bromophenyl)-2-oxoethyl)-2-methoxybenzoate (34). Yield 61%, 11.1 mg, white solid. ^{1}H NMR (400 MHz, $CDCl_3$) δ 7.87 – 7.82 (comp, 2H), 7.69 (d, J = 2.4 Hz, 1H), 7.62 – 7.57 (comp, 2H), 7.34 (dd, J = 8.8, 2.4 Hz, 1H), 6.95 (d, J = 8.8 Hz, 1H), 4.21 (s, 2H), 3.89 (s, 3H), 3.87 (s, 3H). Characterization data matched that reported in the literature [3].

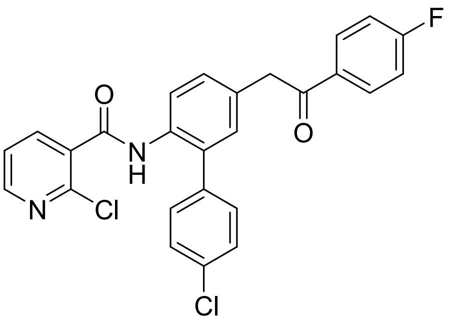

2-Chloro-N-(4'-chloro-5-(2-(4-fluorophenyl)-2-oxoethyl)-[1,1'-biphenyl]-2-yl) nicotinamide (51). Yield 31%, 7.4 mg, yellow solid. ^{1}H NMR (400 MHz, $CDCl_3$) δ 8.44 (d, J = 4.4 Hz, 1H), 8.38 (d, J = 8.4 Hz, 1H), 8.16 – 8.09 (comp, 2H), 8.06 – 8.03 (comp, 2H), 7.44 – 7.39 (comp, 2H), 7.39 – 7.27 (comp, 4H), 7.18 – 7.10 (comp, 3H), 4.29 (s, 2H). Characterization data matched that reported in the literature [3].

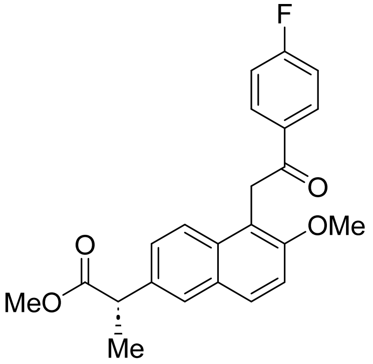

Methyl-(S)-2-(5-(2-(4-fluorophenyl)-2-oxoethyl)-6-methoxynaphthalen-2-yl)propanoate (52). Yield 67%, 12.7 mg, yellow amorphous solid. ^{1}H NMR (400 MHz, $CDCl_3$) δ 8.16 – 8.10 (comp, 2H), 7.78 (d, J = 8.8 Hz, 1H), 7.72 (d, J = 8.8 Hz, 1H), 7.68 (s, 1H), 7.39 (dd, J = 8.8, 2.0 Hz, 1H), 7.28 (d, J = 9.2 Hz, 1H), 7.16 – 7.10 (comp, 2H), 4.71 (s, 2H), 3.90 (s, 3H), 3.84 (q, J = 7.2 Hz, 1H), 3.64 (s, 3H), 1.55 (d, J = 7.1 Hz, 3H). Characterization data matched that reported in the literature [3].

S6.4 General procedure and product characterization for [3+2] cyclization

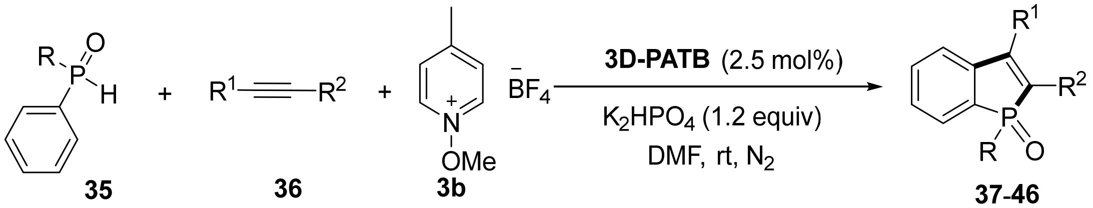

N-methoxy-4-methylpyridinium tetrafluoroborate (0.2 mmol), alkyne **36** (0.1 mmol), phosphine oxide **35** (0.2 mmol) and potassium phosphate dibasic (0.2 mmol), 3D–PATB (2.5 mol% catalyst loading based on the PATB linker) were mixed in anhydrous *N,N*-Dimethylformamide (DMF, 1.0 mL) in a sealed test tube. The resulting mixture was stirred under blue LED irradiation (440-450 nm) at room temperature in a nitrogen atmosphere for 48 hours. After that, the 3D–PATB was filtered off and the solvent was diluted, extracted with ethyl acetate three times. The organic layer was dried over sodium sulfate and filtered. The resulting mixture was concentrated under reduced pressure and purified by flash column chromatography on silica gel to obtain the desired product **37–46**.

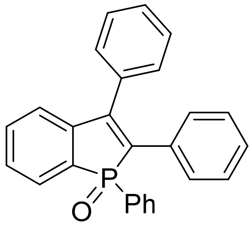

1,2,3-Triphenylphosphindole 1-oxide (37). Yield 73%, 27.6 mg, white solid. ^{1}H NMR (400 MHz, $CDCl_3$) δ 7.80 – 7.74 (comp, 2H), 7.72 – 7.67 (m, 1H), 7.50 – 7.30 (comp, 10H), 7.25 – 7.17 (comp, 3H), 7.13 – 7.03 (comp, 3H). Characterization data matched that reported in the literature [7].

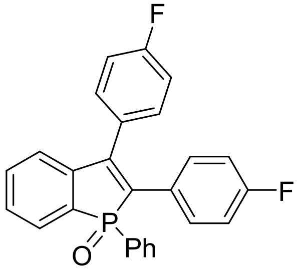

2,3-Bis(4-fluorophenyl)-1-phenylphosphindole 1-oxide (38). Yield 74%, 30.7 mg, colorless solid. ^{1}H NMR (400 MHz, $CDCl_3$) δ 7.77 – 7.68 (comp, 3H), 7.52 – 7.46 (comp, 2H), 7.44 – 7.36 (comp, 3H), 7.33 – 7.27 (comp, 2H), 7.24 – 7.17 (comp, 3H), 7.17 – 7.11 (comp, 2H), 6.84 – 6.77 (comp, 2H). Characterization data matched that reported in the literature [7].

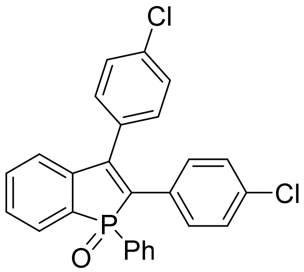

2,3-Bis(4-chlorophenyl)-1-phenylphosphindole 1-oxide (39). Yield 70%, 31.3 mg, colorless solid. ^{1}H NMR (400 MHz, $CDCl_3$) δ 7.81 – 7.63 (comp, 3H), 7.56 – 7.46 (comp, 2H), 7.45 – 7.35 (comp, 5H), 7.28 – 7.24 (comp, 2H), 7.22 – 7.14 (comp, 3H), 7.13 – 7.06 (comp, 2H). Characterization data matched that reported in the literature [7].

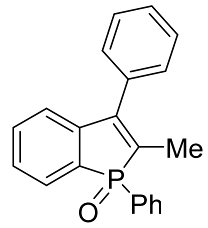

2-Methyl-1,3-diphenylphosphindole 1-oxide (40). Yield 69%. 21.8 mg, colorless solid. ^{1}H NMR (400 MHz, $CDCl_3$) δ 7.74 – 7.63 (comp, 2H), 7.60 – 7.56 (m, 1H), 7.54 – 7.31 (comp, 7H), 7.31 – 7.20 (comp, 3H), 7.04 (dd, *J* = 7.6, 3.2 Hz, 1H), 1.83 (d, *J* = 12.4 Hz, 3H). Characterization data matched that reported in the literature [7].

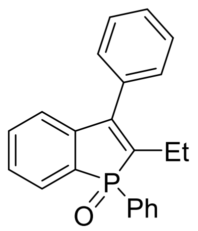

2-Ethyl-1,3-diphenylphosphindole 1-oxide (41). Yield 95%, 31.4 mg, colorless solid. ^{1}H NMR (400 MHz, $CDCl_3$) δ 7.73 – 7.70 (comp, 2H), 7.59 – 7.52 (m, 1H), 7.49 – 7.28 (comp, 7H), 7.28 – 7.20 (comp, 3H), 6.96 (dd, *J* = 7.6, 3.2 Hz, 1H), 2.51 – 2.30 (m, 1H), 2.28 – 2.08 (m, 1H), 0.88 (t, *J* = 7.6 Hz, 3H). Characterization data matched that reported in the literature [7].

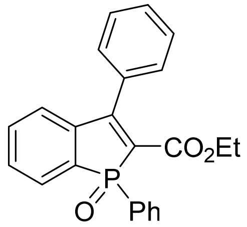

Ethyl 1,3-diphenylphosphindole-2-carboxylate 1-oxide (42). Yield 72%, 27.0 mg, colorless solid. ^{1}H NMR (400 MHz, $CDCl_3$) δ 7.83 – 7.70 (comp, 3H), 7.58 – 7.34 (comp, 10H), 7.25 – 7.19 (m, 1H), 4.12 – 4.03 (m, 1H), 4.03 – 3.93 (m, 1H), 0.96 (t, *J* = 7.6 Hz, 3H). Characterization data matched that reported in the literature [7].

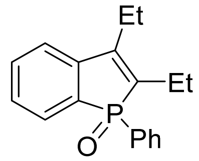

2,3-Diethyl-1-phenylphosphindole 1-oxide (43). Yield 63%, 17.8 mg, white solid. ^{1}H NMR (400 MHz, $CDCl_3$) δ 7.69 – 7.61 (comp, 2H), 7.58 – 7.52 (m, 1H), 7.51 – 7.45 (comp, 2H), 7.42 – 7.34 (comp, 3H), 7.30 – 7.24 (m, 1H), 2.63 (q, *J* = 7.6 Hz, 2H), 2.57 – 2.44 (m, 1H), 2.41 – 2.28 (m, 1H), 1.23 (t, *J* = 7.6 Hz, 4H), 1.00 (t, *J* = 7.6 Hz, 3H). Characterization data matched that reported in the literature [7].

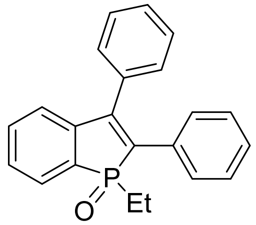

1-Ethyl-2,3-diphenylphosphindole 1-oxide (44). Yield 73%. 24.1 mg, colorless oil. ^{1}H NMR (400 MHz, $CDCl_3$) δ 7.85 – 7.78 (m, 1H), 7.53 – 7.45 (m, 1H), 7.45 – 7.34 (comp, 7H), 7.31 – 7.26 (m, 1H), 7.23 – 7.18 (comp, 3H), 7.18 – 7.13 (m, 1H), 2.25 – 2.08 (m, 1H), 2.03 – 1.92 (m, 1H), 0.98 (dt,

J = 18.8, 7.6 Hz, 3H). Characterization data matched that reported in the literature [7].

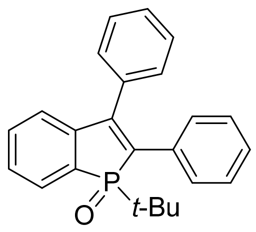

1-(Tert-butyl)-2,3-diphenylphosphindole 1-oxide (45). Yield 60%, 21.5 mg, colorless solid. ^{1}H NMR (400 MHz, $CDCl_3$) δ 7.87 – 7.77 (m, 1H), 7.50 – 7.30 (comp, 7H), 7.24 – 7.11 (comp, 5H), 1.08 (d, J = 15.2 Hz, 9H). Characterization data matched that reported in the literature [7].

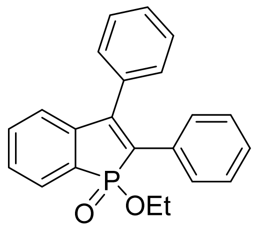

1-Ethoxy-2,3-diphenylphosphindole 1-oxide (46). Yield 77%, 31.3 mg, colorless oil. ^{1}H NMR (400 MHz, $CDCl_3$) δ 7.82 – 7.70 (m, 1H), 7.46 – 7.35 (comp, 6H), 7.30 – 7.19 (comp, 5H), 7.15 – 7.09 (m, 1H), 4.27 – 4.00 (m, 2H), 1.26 (t, J = 3.6 Hz, 3H). Characterization data matched that reported in the literature [7].

S6.5 General procedure for the scale-up synthesis of 4

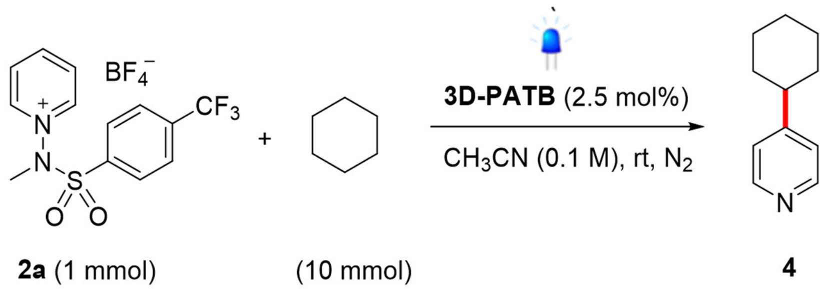

N-aminopyridinium salt **2a** (1 mmol, 0.4 g), 3D–PATB (2.5 mol% catalyst loading based on the PATB linker) and cyclohexane (10 mmol) were mixed in anhydrous acetonitrile (MeCN, 10 mL) in a sealed test tube. The resulting mixture was stirred under blue LED irradiation (440-450 nm) at room temperature in a nitrogen atmosphere for 48 hours. After that, 3D–PATB was filtered off, the solvent was removed under vacuum, and the residue was subjected to column chromatography on silica gel to give product **4** (0.129g, 80%).

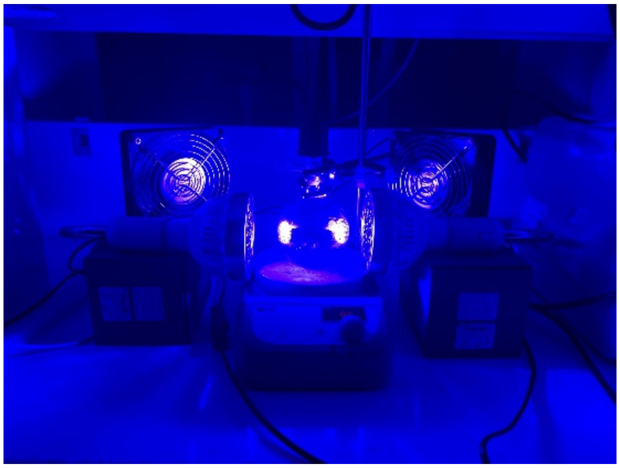

Figure S21. General scale-up reaction setup.

S7 Mechanistic Studies

S7.1 UV-Vis diffuse reflectance spectroscopy (DRS) analysis

UV-Vis diffuse reflectance spectroscopy (DRS) was employed to characterize the EDA interaction between 3D-PATB and pyridinium salts.

UV-Vis DRS spectrum of 3D–PATB: 5.0 mg sample was used to determine absorption from 400 nm to 800 nm.

UV-Vis DRS spectrum of **2a**: 5.0 mg sample was used to determine absorption from 400 nm to 800 nm.

UV-Vis DRS spectrum of 3D–PATB and Pyridium salt **2a**: 1.5 mL of **2a** (0.005 M in MeCN) and 1.5 mL of 3D–PATB (0.005 M in MeCN, based on the PATB linker) were added to a centrifugal tube (5 mL). The centrifugal tube was then sealed and incubated for 5 min at ambient temperature with an ultrasonicator. The sample was then centrifuged and the supernatant was discarded. The centrifuged material was dried and tested for absorption from 400 nm to 800 nm.

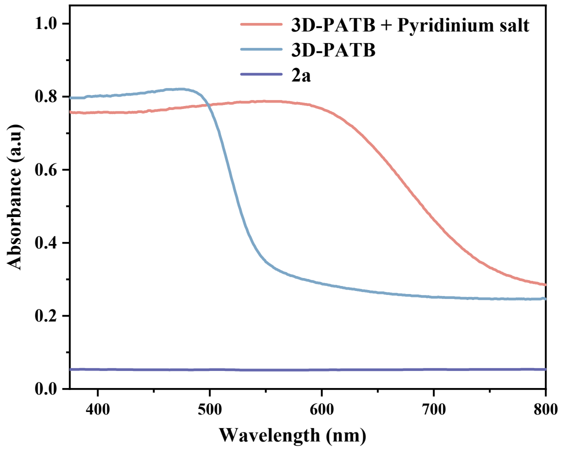

Figure S22. UV-Vis DRS of pyridium salt **2a**, 3D–PATB and the mixture of **2a** with 3D–PATB.

S7.2 UV-Vis determination of EDA complexes

UV-Vis determination of EDA interaction between 3D-PATB and **2a**. UV-Vis spectrum of **2a**: 1.0 mL of **1a** (0.0004 M in MeCN) was added to a cuvette (3.5 ml) along with 1.0 mL of anhydrous MeCN. The cuvette was then sealed and incubated for 5 minutes at ambient temperature with a mixer. Afterwards the sample was tested for absorption from 200 nm to 600 nm.

UV-Vis spectrum of 3D-PATB: 1.0 mL 3D-PATB (0.0004 M in MeCN, based on the linker) was added to a cuvette (3.5 ml) along with 1.0 mL of anhydrous MeCN. The cuvette was then sealed and ultrasonicated for 5 minutes at ambient temperature. Afterwards the sample was tested for absorption from 200 nm to 600 nm.

UV-Vis spectrum of the mixture: 1.0 mL of **2a** (0.0004 M in MeCN) and 1.0 mL of 3D-PATB (0.0004 M in MeCN, based on the linker) were added to a cuvette (3.5 ml). The cuvette was then sealed and ultrasonicated for 5 minutes at ambient temperature. Afterwards the sample was tested for absorption from 200 nm to 600 nm. The EDA interactions between 2D-PATB, 2D-PATB-CN, and substrate **2a** were also analyzed according to the procedure described above.

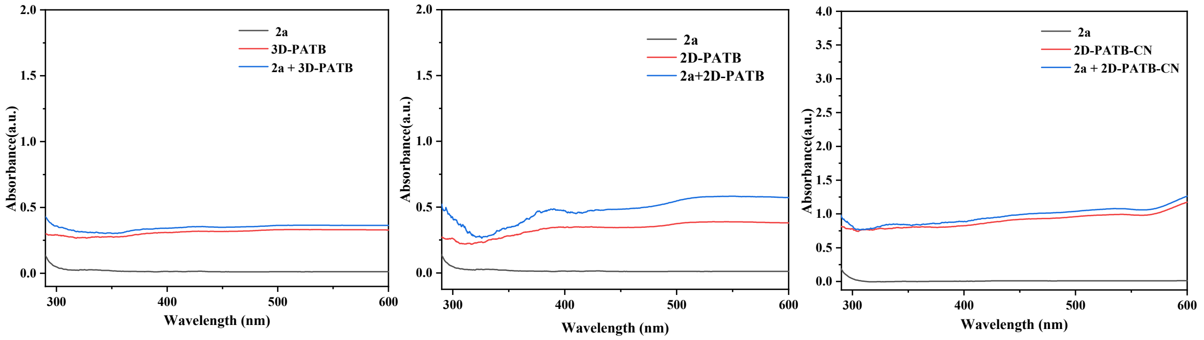

Figure S23. UV-vis spectra of **2a**, 3D-PATB, and their mixture; 2D-PATB, and their mixture; 2D-PATB-CN, and their mixture in MeCN ($2.0{\times}10^{-4}$M).

UV-Vis determination of EDA interaction between 3D-PATB and **3a**. UV-Vis spectrum of **3a**: 1.0 mL of 1a (0.0004 M in MeCN) was added to a cuvette (3.5 ml) along with 1.0 mL of anhydrous MeCN. The cuvette was then sealed and incubated for 5 minutes at ambient temperature with a mixer. Afterwards the sample was tested for absorption from 200 nm to 600 nm.

UV-Vis spectrum of 3D-PATB: 1.0 mL 3D-PATB (0.0004 M in MeCN, based on the linker) was added to a cuvette (3.5 mL) along with 1.0 mL of anhydrous MeCN. The cuvette was then sealed and ultrasonicated for 5 minutes at ambient temperature. Afterwards the sample was tested for absorption from 200 nm to 600 nm.

UV-Vis spectrum of the mixture: 1.0 mL of **3a** (0.0004 M in MeCN) and 1.0 mL of 3D-PATB (0.0004 M in MeCN, based on the linker) were added to a cuvette (3.5 ml). The cuvette was then sealed and ultrasonicated for 5 minutes at ambient temperature. Afterwards the sample was tested for absorption from 200 nm to 600 nm.

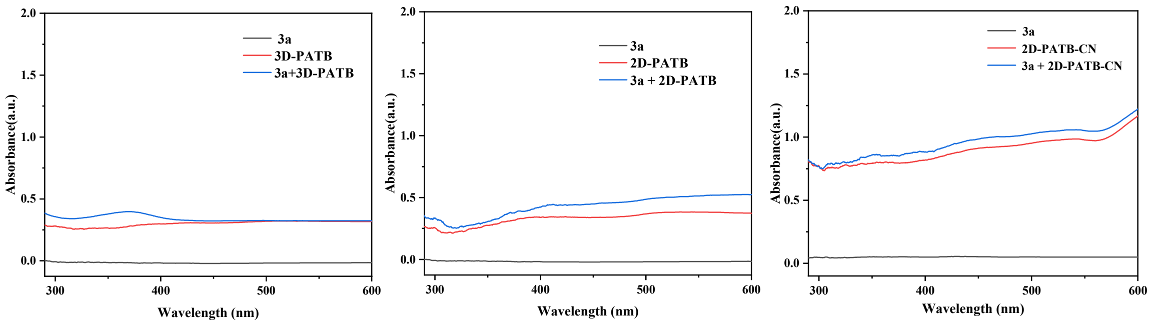

Figure S24. UV-vis spectra of **3a**, 3D-PATB, and their mixture; **3a**, 2D-PATB, and their mixture; **3a**, 2D-PATB-CN, and their mixture in MeCN (2.0×10^{-4}M).

UV-Vis determination of EDA interaction between 3D-PATB and **23a**. UV-Vis spectrum of **23a**: 1.0 mL of 1a (0.0004 M in MeCN) was added to a cuvette (3.5 ml) along with 1.0 mL of anhydrous MeCN. The cuvette was then sealed and incubated for 5 minutes at ambient temperature with a mixer. Afterwards the sample was tested for absorption from 200 nm to 600 nm.

UV-Vis spectrum of 3D-PATB: 1.0 mL 3D-PATB (0.0004 M in MeCN, based on the linker) was added to a cuvette (3.5 ml) along with 1.0 mL of anhydrous MeCN. The cuvette was then sealed and ultrasonicated for 5 minutes at ambient temperature. Afterwards the sample was tested for absorption from 200 nm to 600 nm.

UV-Vis spectrum of the mixture: 1.0 mL of **23a** (0.0004 M in MeCN) and 1.0 mL of 3D-PATB (0.0004 M in MeCN, based on the linker) were added to a cuvette (3.5 ml). The cuvette was then sealed and ultrasonicated for 5 minutes at ambient temperature. Afterwards the sample was tested for absorption from 200 nm to 600 nm.

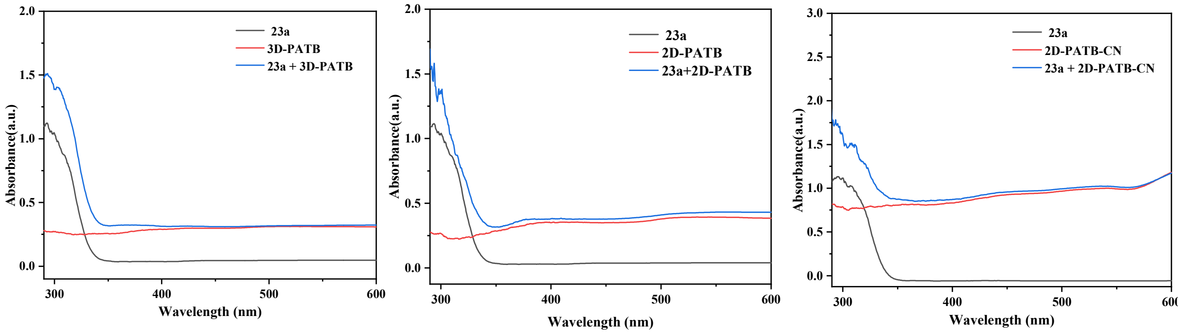

Figure S25. UV-vis spectra of **23a**, 3D-PATB, and their mixture; **23a**, 2D-PATB, and their mixture; **23a**, 2D-PATB-CN, and their mixture in MeCN (2.0×10^{-4}M).

S7.3 UV-vis analyses of the mixtures of 3D-PATB and 2a with different ratios and Job's Plot

Two stock solutions of **2a** and 3D-PATB in MeCN were independently prepared at a concentration of 0.0016M. For the Job's plot analysis, seven samples with different mixing ratios of **2a** and 3D-PATB were prepared by combining appropriate volumes of the two stock solutions, while maintaining a constant total volume of 2.0 mL. The compositions corresponded to volume ratios of **2a**/3D-PATB of 2.0:0, 1.75:0.25, 1.5:0.5, 1.0:1.0, 0.5:1.5, 0.25:1.75, and 0:2.0. UV–vis absorption spectra of the resulting solutions were recorded over the wavelength range of 300 – 800 nm, and the spectra are summarized in **Figure 26**. The absorbance at 385 nm was extracted for each sample and is listed in **Table S8**.

Job's plot analysis was constructed based on the absorbance values at 385 nm derived from the UV–vis spectra. For reference, the absorbance of **2a** at 385 nm in the absence of 3D-PATB was measured to be 0.0264. The corresponding absorbance values and calculations used for the Job's plot are provided in **Table S8**.

Table S8. UV-vis results for Job's plot.

Entry	V (2a, mL)	V (3D-PATB, mL)	X (2a) (mmol)	Normalization X (1a)	A (385 nm)	ΔA (385 nm)	X (2a) * ΔA
**1**	2	0	0.0032	1	0.0264	0	0.000000
**2**	1.75	0.25	0.0028	0.875	0.2410	0.2146	0.187775
**3**	1.5	0.5	0.0024	0.75	0.5008	0.4744	0.355800
**4**	1	1	0.0016	0.5	0.9119	0.8855	0.442750
**5**	0.5	1.5	0.0008	0.25	1.2343	1.2079	0.301975
**6**	0.25	1.75	0.0004	0.125	1.5032	1.4768	0.184600
**7**	0	2	0	0	1.8346	1.8082	0.000000

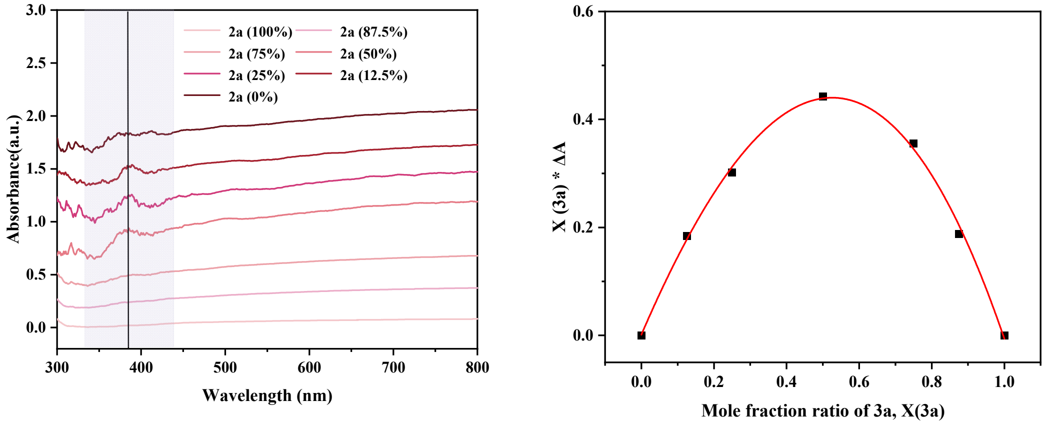

Figure 26. UV-vis spectra of mixtures of **2a** and 3D-PATB with different ratios of **2a** in MeCN (left). Job's plot based on UV-vis data in Table S8 (right).

S7.4 UV-vis analyses of the mixtures of 3D-PATB and 3a with different ratios and Job's Plot

Two stock solutions of **3a** and 3D-PATB in MeCN were independently prepared at a concentration of 0.0008 M. For the Job's plot analysis, seven samples with different mixing ratios of **3a** and 3D-PATB were prepared by combining appropriate volumes of the two stock solutions, while maintaining a constant total volume of 2.0 mL. The compositions corresponded to volume ratios of **3a**/3D-PATB of 2.0:0, 1.75:0.25, 1.5:0.5, 1.0:1.0, 0.5:1.5, 0.25:1.75, and 0:2.0. UV–vis absorption spectra of the resulting solutions were recorded over the wavelength range of 300 – 800 nm, and the spectra are summarized in **FigureS27**. The absorbance at 385 nm was extracted for each sample and is listed in **Table S9**.

Job's plot analysis was constructed based on the absorbance values at 385 nm derived from the UV–vis spectra. For reference, the absorbance of **3a** at 385 nm in the absence of 3D-PATB was measured to be 0.0264. The corresponding absorbance values and calculations used for the Job's plot are provided in **Table S9**.

Table S9. UV-vis results for Job's plot.

Entry	V (3a, mL)	V (3D-PATB, mL)	X (3a) (mmol)	Normalization X (1a)	A (370 nm)	ΔA (370 nm)	X (3a) * ΔA
**1**	2	0	0.0016	1	0.00427	0.00000	0.00000
**2**	1.75	0.25	0.0014	0.875	0.07399	0.06972	0.06100

**3**	1.5	0.5	0.0012	0.75	0.13780	0.13353	0.10015
**4**	1	1	0.0008	0.5	0.26293	0.25866	0.12933
**5**	0.5	1.5	0.0004	0.25	0.33857	0.33430	0.08357
**6**	0.25	1.75	0.0002	0.125	0.43724	0.43297	0.05412
**7**	0	2	0	0	0.66669	0.66242	0.00000

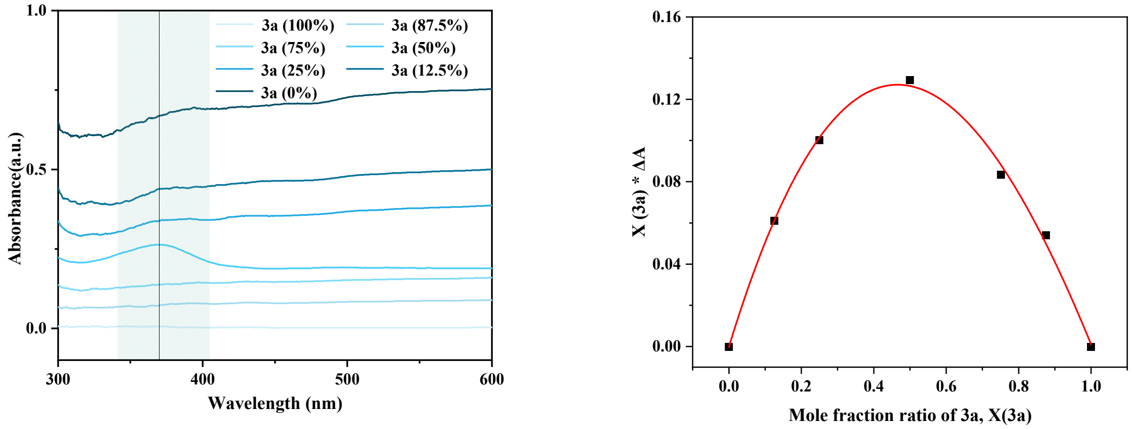

Figure 27. UV-vis spectra of mixtures of **3a** and 3D-PATB with different ratios of **3a** in MeCN (left). Job's plot based on UV-vis data in Table S9 (right).

S7.5 Steady-state fluorescence analyses of the mixtures of 3D-PATB and 2a with different ratios

Stock solutions of 3D-PATB, 2D-PATB, 2D-PATB-CN (based on the linker), and **2a** were individually prepared in acetonitrile at a concentration of 0.001 mol/L. Subsequently, a series of diluted solutions with varying concentration gradients were prepared according to the following table for steady-state fluorescence spectroscopy measurements.

Table S10. Steady-state fluorescence experiments.

**2a** (uL)	3D-PATB-COF (uL)	MeCN (uL)	**2a** (mol/L)
0	190	1810	0
30	190	1780	0.015
70	190	1740	0.035
110	190	1700	0.055
150	190	1660	0.075
170	190	1640	0.085
200	190	1610	0.1

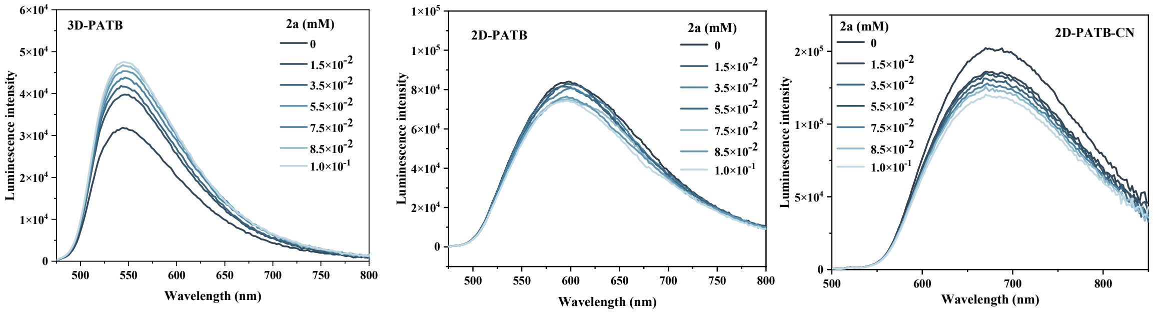

Figure S28. Luminescence emission spectra as a function of **2a** concentration upon excitation at 450 nm.

S7.6 Radical capture experiments for C–H pyridylation

N-aminopyridinium salt (**2a**, 0.1 mmol), 3D–PATB (2.5 mol % based on the PATB linker), cyclohexane (1 mmol) and (2,2,6,6-tetramethylpiperidin-1-yl)oxyl (TEMPO) (0.5 mmol, 5.0 equiv) were mixed with anhydrous acetonitrile (MeCN, 1.0 mL) in a sealed test tube. The resulting mixture was stirred under blue LED irradiation (440-450 nm) at room temperature in a N_2 atmosphere for 48 hours. After that, 3D–PATB was filtered off, the solvent was removed under vacuum, and the residue was subjected to LCMS analysis.

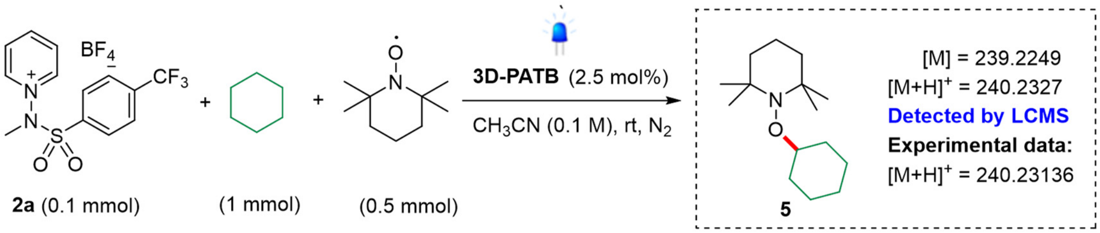

Figure S29. Radical capture experiment with TEMPO.

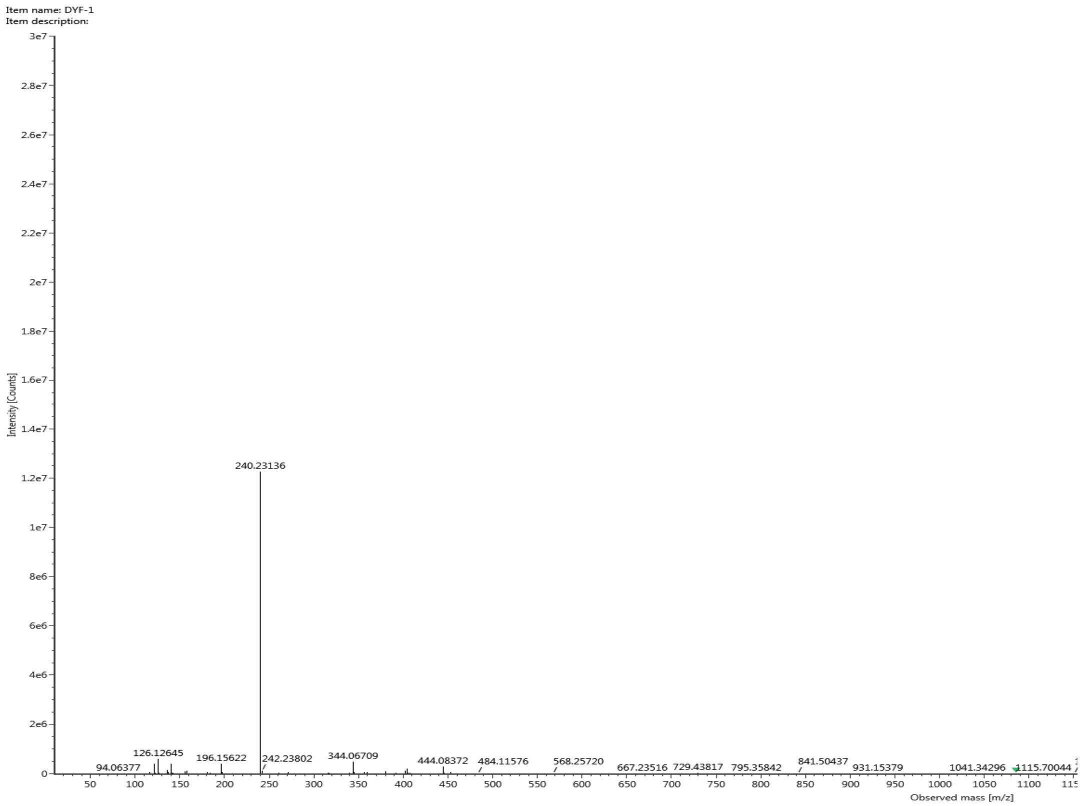

Figure S30. Cyclohexyl-TEMPO adduct (Mw = 240.23136) detected on LC-MS.

N-methoxy pyridinium methylsulfate (**3a**, 0.1 mmol), 3D–PATB (2.5 mol % based on the PATB linker), tetrahydrofuran (7.2 mmol), $NaHCO_3$ (0.2 mmol) and (2,2,6,6-tetramethylpiperidin-1-yl)oxyl (TEMPO) (0.5 mmol, 5.0 equiv) were mixed with anhydrous acetonitrile (MeCN, 1.0 mL) in a sealed test tube. The resulting mixture was stirred under blue LED irradiation (440-450 nm) at room temperature in a N_2 atmosphere for 24 hours. After that, 3D–PATB was filtered off, the solvent was removed under vacuum, and the residue was subjected to LCMS analysis.

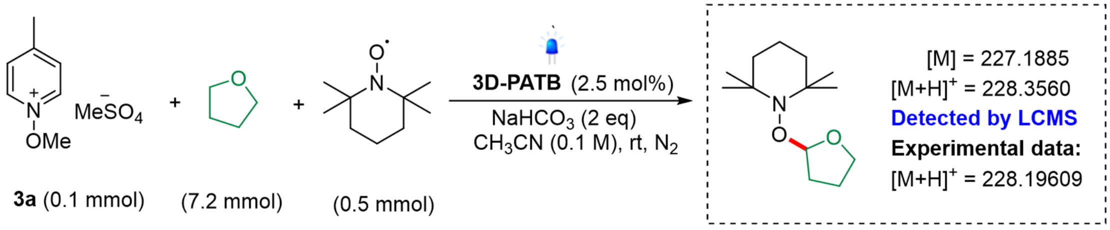

Figure S31. Radical capture experiment with TEMPO.

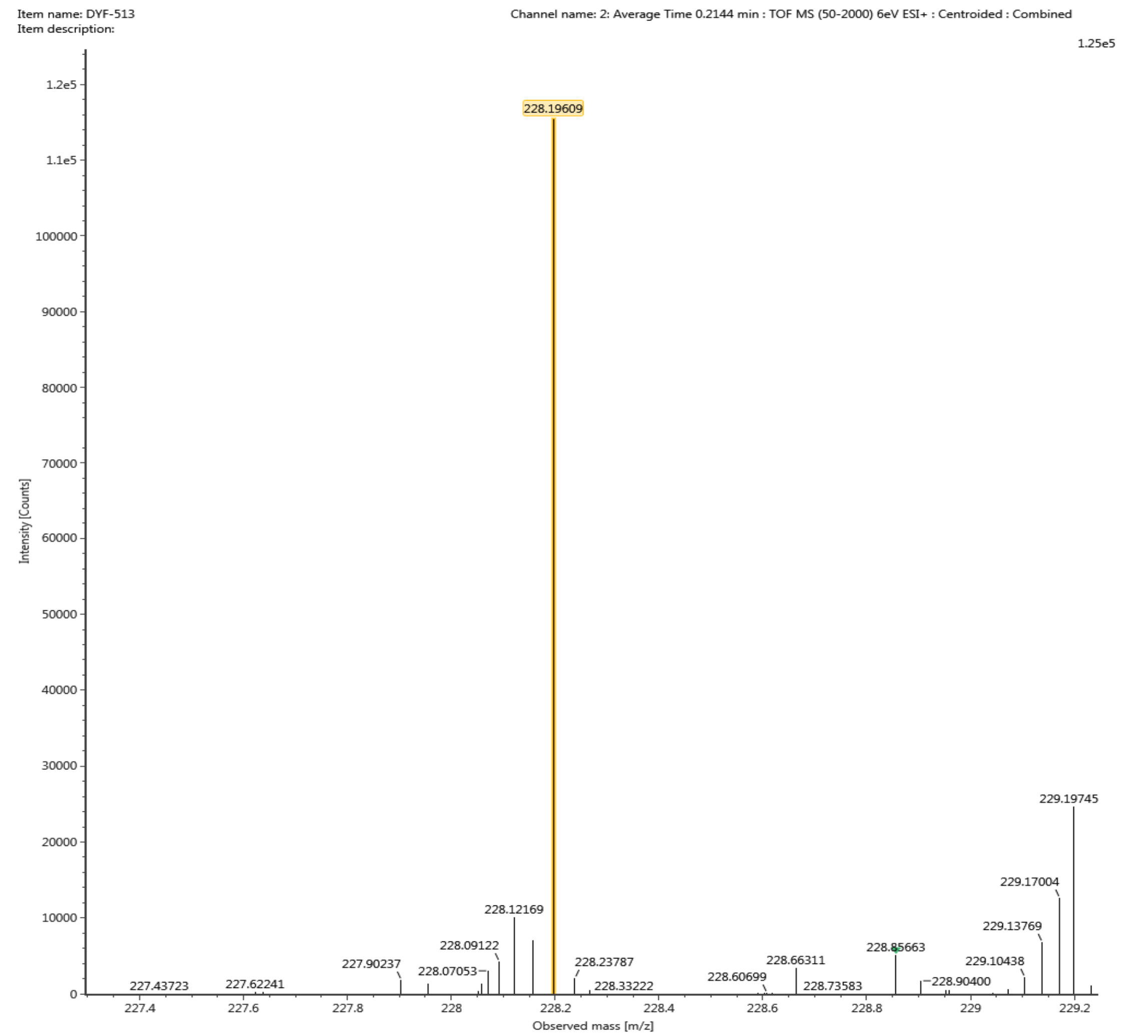

Figure S32. Tetrahydrofuran-TEMPO adduct (Mw = 228.19609) detected on LC-MS.

S7.7 Light on/off experiments for C–H pyridylation

N-aminopyridinium salt (**2a**, 0.1 mmol), 3D–PATB (2.5 mol % based on the PATB linker), cyclohexane (1 mmol) were mixed in anhydrous acetonitrile (MeCN, 1.0 mL) in a sealed test tube. Six parallel reactions were carried out simultaneously. The resulting mixture was stirred under blue LED irradiation (440-450 nm) at room temperature in a N_2 atmosphere for 10 hours. After that, the light was turned off, and one of the parallel reactions was quenched and subjected to ^{1}H NMR analysis. The other reactions were stirred in the dark for another 10 hours. Then, one of them was quenched and subjected to ^{1}H NMR analysis. The same light on/off procedure was repeated until all the parallel reactions were quenched and analyzed.

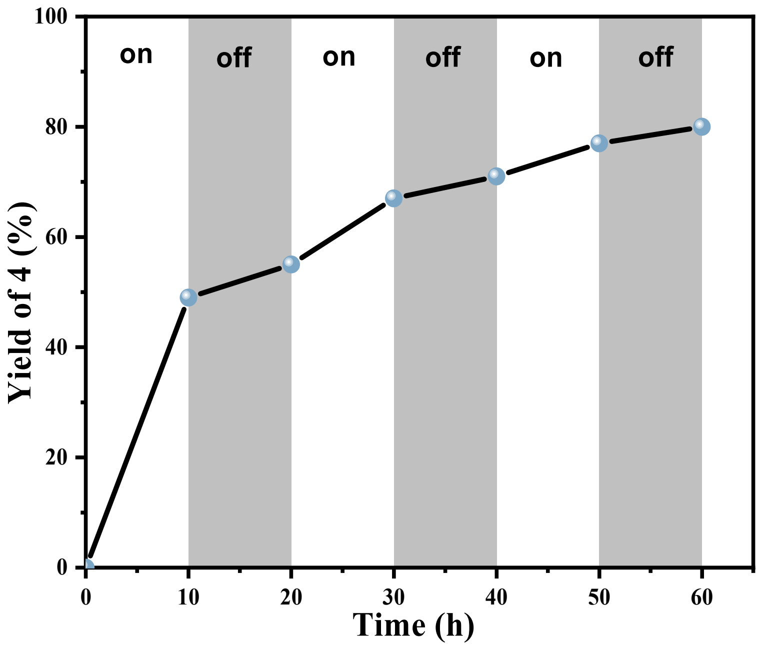

Figure S33. Light on/off experiments for the synthesis of **4**.

S7.8 Quantum yield measurements

The photon flux of the spectrophotometer was determined by standard ferrioxalate actinometry following the reported procedure by Yoon.[8] A 0.15 M ferrioxalate solution was prepared by dissolving potassium ferrioxalate hydrate (1.47 g) in 20 mL of 0.05 M H_2SO_4. A buffered 1,10-phenanthroline solution was prepared by dissolving 1,10-phenanthroline (25 mg) and sodium acetate (5.63 g) in 25 mL of 0.5 M H_2SO_4. Both solutions were stored in the dark prior to use.

For photon flux determination, 2.0 mL of the ferrioxalate solution was transferred to a quartz cuvette and irradiated for 100 s at a wavelength range of 440 – 450 nm. After irradiation, 0.35 mL of the phenanthroline solution was added, and the resulting mixture was kept in the dark for 1 h to ensure complete coordination of the generated ferrous ions with phenanthroline. The absorbance of the resulting solution was then measured at 510 nm. A non-irradiated ferrioxalate sample was treated identically and used as a reference. The conversion was calculated according to **eq. S1.**

$$mol\ Fe^{2+} = \frac{V \cdot \Delta A}{l \cdot \varepsilon} = \frac{0.00235\ \times (1.26 - 0.72)}{1.0 \times 11{,}100} = 1.14 \times 10^{-7}\ mol$$

(eq. S1)

Where V is the total volume (0.00235 L) of the solution after addition of phenanthroline, ΔA is the difference in absorbance at 510 nm between the irradiated and non-irradiated solutions, l is the path length (1.000 cm), and ε is the molar absorptivity at 510 nm (11,100 L $mol^{-1}cm^{-1}$). The photon flux can be calculated using **eq. S2**.

$$Photon\ flux = \frac{mol\ Fe^{2+}}{\emptyset \cdot t \cdot f} = \frac{1.14 \times 10^{-7}}{0.92 \times 100 \times 0.9975} = 1.24 \times 10^{-9}\ einstein\ s^{-1}$$

(eq. S2)

Where $\emptyset$ is the quantum yield for the ferrioxalate actinometer (0.92 for a 0.15 M solution at λ = 445 nm), t is the time (100 s), and f is the fraction of light absorbed at λ = 445 nm. The value of f is shown in **eq S3**.

Determination of fraction of light absorbed at 445 nm for the ferrioxalate solution:

The fraction of light absorbed (f) by this solution was calculated using **eq. S3**, where A is the measured absorbance of the above ferrioxalate solution at 445 nm which was measured to be 2.6146, indicating the fraction of light absorbed (f) is 0.9975. Finally, the photon flux was calculated to be 1.24×10^{-9} einstein s^{-1}.

$$f = 1 - 10^{-A} = 0.9975$$

(eq. S3)

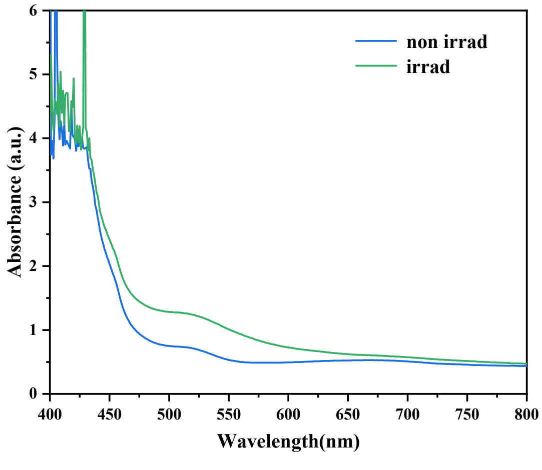

Figure S34. Absorption spectra of the irradiation experiment and non-irradiation experiment.

Quantum yield calculation:

The reaction was performed following general procedure with *N*-aminopyridinium salt **2** (0.1 mmol), 3D–PATB (2.5 mol % catalyst loading based on the PATB linker) and hydrocarbon **1** (1 mmol) were mixed in anhydrous acetonitrile (MeCN, 1.0 mL) in a sealed test tube. The resulting mixture was stirred under blue LED irradiation (440-450 nm) at room temperature under a nitrogen atmosphere for 7 hours. After irradiation, the yield of product formed was determined by NMR to be 40% using CH_2Br_2 as an internal standard. The quantum yield was determined as 1.3 as shown in **eq. S4**

$$\emptyset = \frac{mol\ Prod}{Photon\ flux \cdot t \cdot f} = \frac{0.40 \times 0.1 \times 10^{-3}}{1.24 \times 10^{-9} \times 25200 \times 0.9918} = 1.3$$

(eq. S4)

Absorbance of Catalyst: The absorbance of 3D-PATB-COF in MeCN was measured at the reaction concentration of 1.0×10^{-3} M (Supplementary **Figure S35**). The absorbance at 445 nm is 2.0855, indicating the fraction of light absorbed (f) is 0.9918.

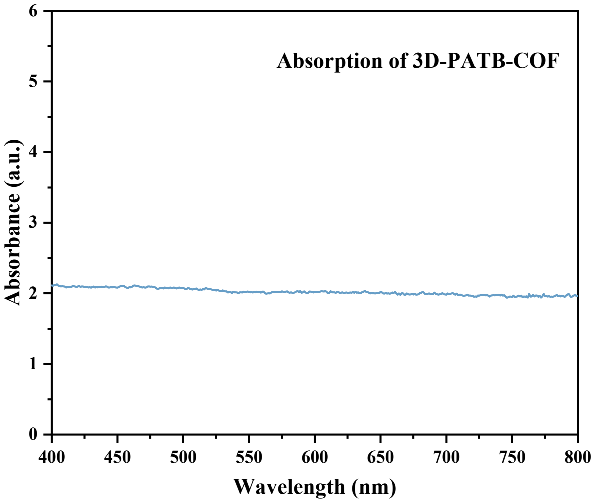

Figure S35. Absorption spectra of 1.0×10^{-3} M solution of 3D-PATB-COF in MeCN.

S7.9 Proposed reaction mechanism

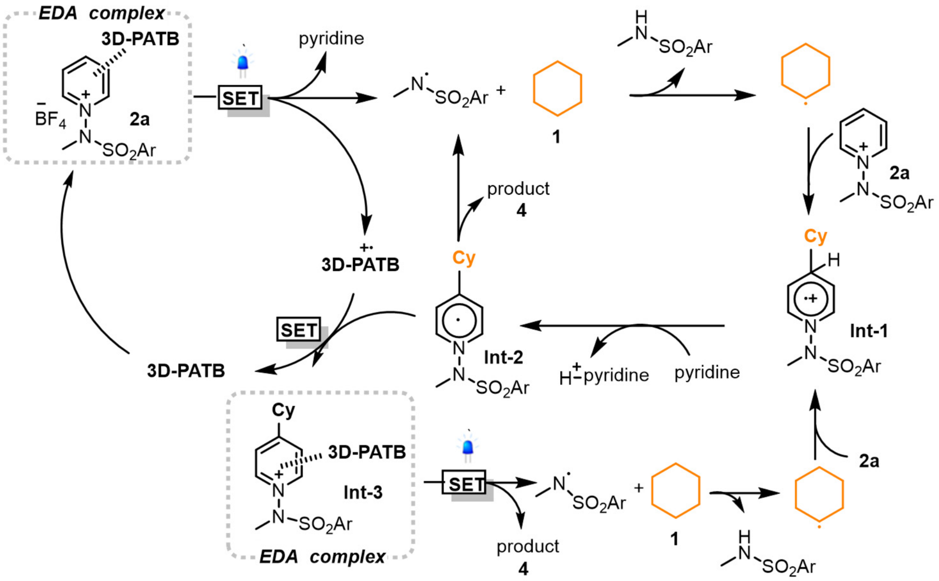

Figure S36. The proposed reaction mechanism for C4–site-selective C–H pyridylation.

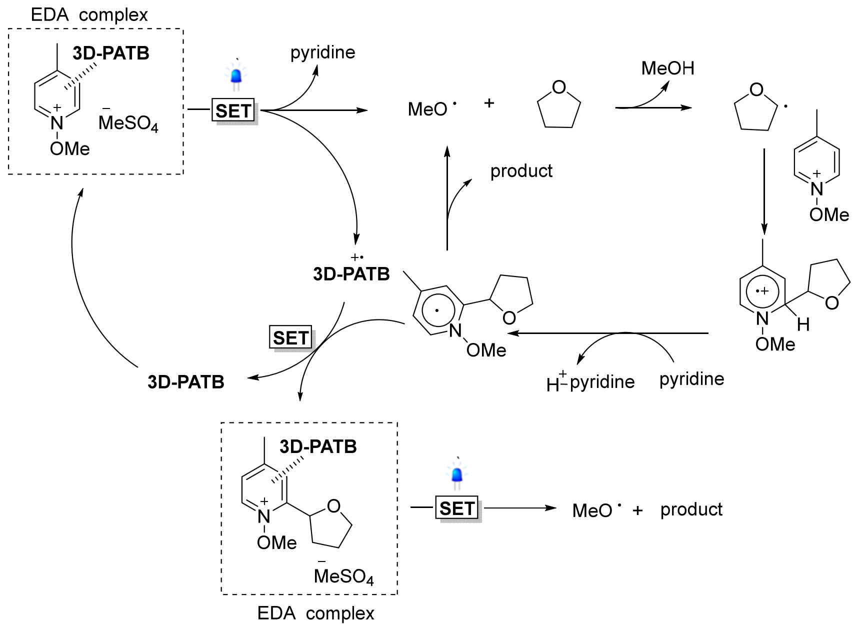

Figure S37. The proposed reaction mechanism for C2–site-selective C–H pyridylation.

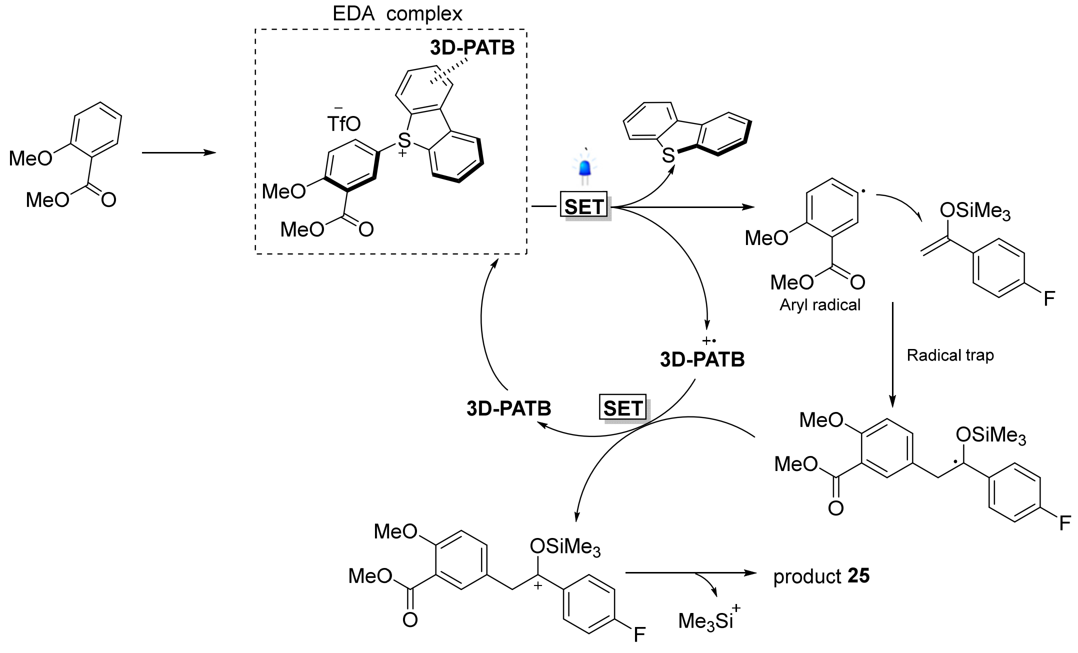

Figure S38. The proposed reaction mechanism for formal C–H alkylation.

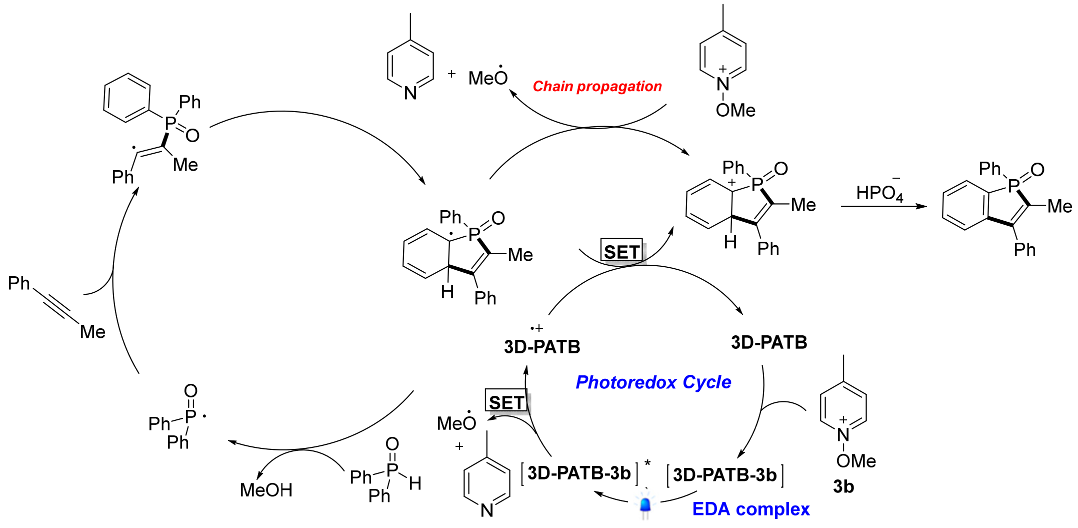

Figure S39. The proposed reaction mechanism for [3+2] cyclization.

S8 DFT computational studies

Spin-polarized density functional theory (DFT) calculations were performed using the Vienna Ab Initio Simulation Package (VASP) [9,10]. The Perdew-Burke-Ernzerhof (PBE) functional [11] was employed to account for exchange and correlation effects. The electron–ion interactions were described using the projected augmented wave (PAW) method [12]. A plane-wave basis set with a cutoff energy of 400 eV was applied. The Gaussian smearing method, with a smearing width of 0.05 eV, was used to determine partial occupancies. Van

der Waals interactions were considered through the DFT-D3 method [13]. To balance computational cost and accuracy, the convergence tolerances were set to 10^{-5} eV for energy variations and 0.03 eV/Å for forces on each atom during structure optimization. Periodic boundary conditions were applied throughout the calculations, with a vacuum layer of 15 Å added to the facets. The Brillouin zone was sampled using a Monkhorst–Pack mesh with a 3 × 3 × 1 grid in reciprocal space. Structure optimization was considered complete when the residual Hellmann-Feynman forces were less than 0.02 eV/Å.

Additional DFT calculations were performed using the hybrid B3LYP-D3 functional, including Grimme's D3 dispersion correction [14,15,16], as implemented in the Gaussian16 program [17]. The self-consistent reaction field (SCRF) theory was employed [18]. To obtain more accurate molecular structures, we extracted relevant molecular fragments from the VASP-optimized periodic models and performed geometry optimizations with partial atomic coordinates constrained, using the def2-SVP basis set for all elements. To achieve more accurate relative energies, single-point calculations using the M06 functional [19] with the def2-tzvp basis set were carried out based on the optimized molecular geometries. Energy decomposition analysis [20] and independent gradient model based on Hirshfeld partition (IGMH） analysis [21] performed using Multiwfn software [22]. The 3D structures of the molecules were visualized using CYL View [23].

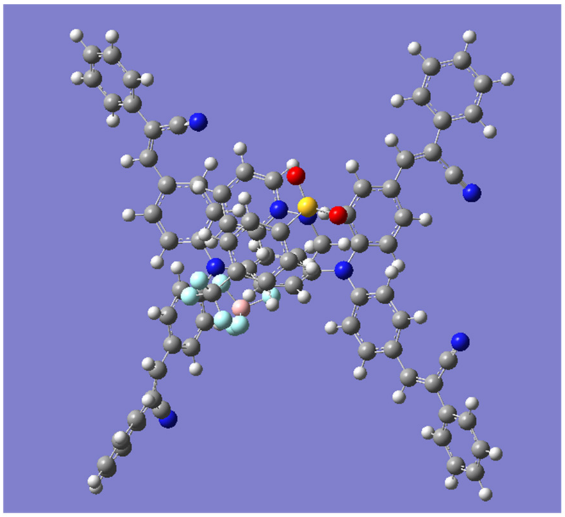

Interaction energy components between all fragments:

	Electrostatic	Repulsion	Dispersion	Total
Frag 1 -- Frag 2:	-25.81	93.31	-119.55	-52.05

Figure S40. DFT calculations of interaction energy components between substrate **2a** and PATB core in 2D–PATB–CN.

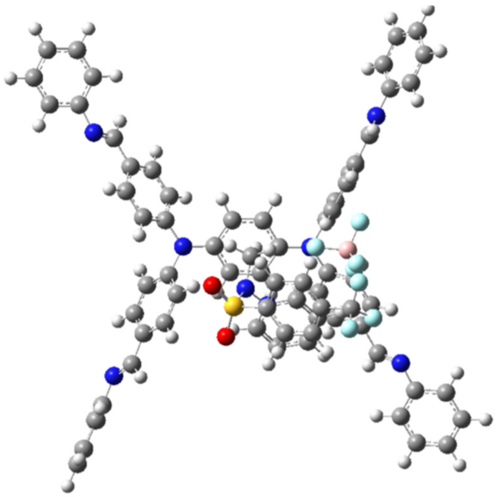

Interaction energy components between all fragments:

	Electrostatic	Repulsion	Dispersion	Total
Frag 1 -- Frag 2:	-29.02	95.82	-138.28	-71.48

Figure S41. DFT calculations of interaction energy components between substrate **2a** and PATB core in 2D–PATB.

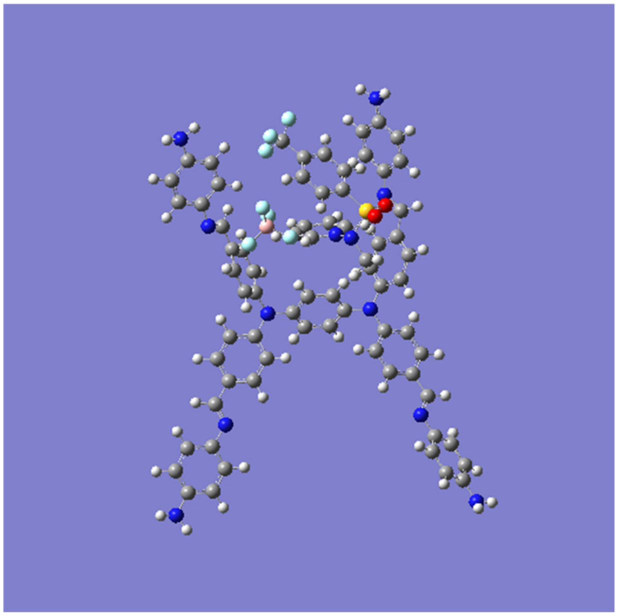

Interaction energy components between all fragments:

	Electrostatic	Repulsion	Dispersion	Total
Frag 1 -- Frag 2:	-17.38	114.10	-173.80	-77.08

Figure S42. DFT calculations of interaction energy components between substrate **2a** and PATB core in 3D–PATB.

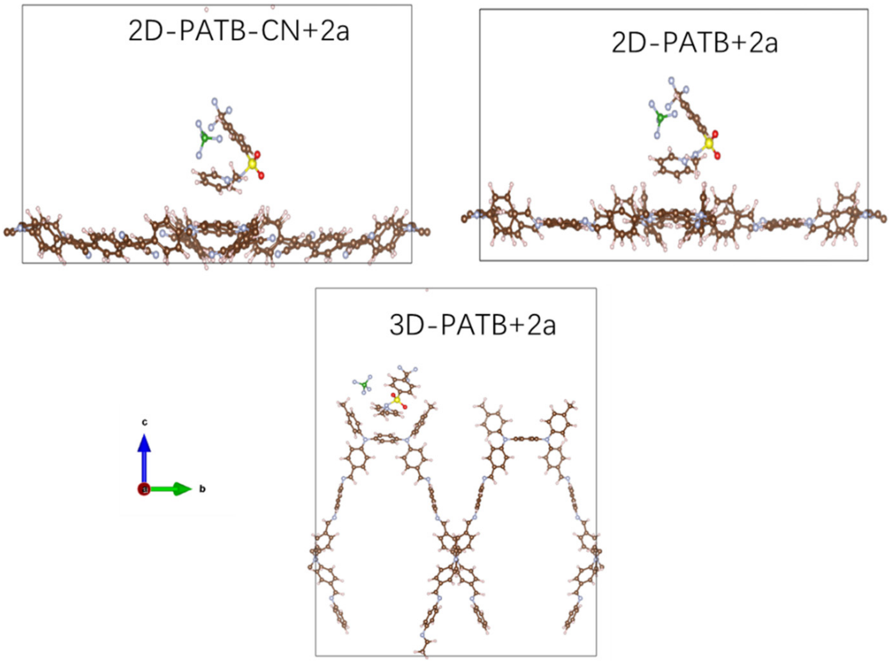

Figure S43. The binding configurations between **2a** and extended COFs surface

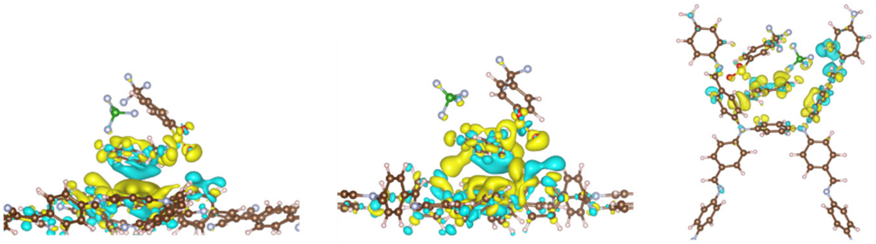

	2D-PATB-CN	2D-PATB	3D-PATB
Eads	-0.55808 eV	-0.56267 eV	-1.00254 eV

Figure S44. Charge density difference and the Eads of 2D-PATB-CN, 2D-PATB, and 3D-PATB.

Table S11. Cartesian coordinates of 2D–PATB–CN and **2a**.

Symbol	X	Y	Z
C	6.2894280	-10.5728060	0.2526330
C	6.0357830	-9.2135850	0.4452860
C	5.4856830	-8.4225920	-0.5820870
C	5.2141120	-9.0447950	-1.8162860
C	5.4672950	-10.4034450	-2.0060460
C	6.0072860	-11.1799690	-0.9737350
C	5.2294310	-6.9722330	-0.3561380
C	-2.5790470	7.1460150	0.0173020
C	1.4191100	0.7443320	-2.6443330

C	0.1176340	1.1856290	-2.8743040
C	-0.9603150	0.6122780	-2.1803000
C	-2.6189580	2.4369850	-2.3227810
C	-3.3254950	0.0920570	-2.2751950
C	-4.3034550	0.1409030	-1.2745760
C	-5.2251570	-0.8890670	-1.1534370
C	-5.2222750	-1.9907860	-2.0346600
C	-4.2332890	-2.0213100	-3.0416490
C	-3.3024740	-0.9963600	-3.1572760
C	-3.8852720	2.8785310	-2.7743210
C	-4.2521290	4.2114440	-2.6700570
C	-3.3956240	5.1829980	-2.1012250
C	-2.1042040	4.7477410	-1.7318020
C	-1.7231380	3.4188170	-1.8392290
C	-3.8872650	6.5396620	-1.9270830
C	-6.2311600	-3.0205610	-1.8178800
C	-7.9855230	-5.2554690	-0.8365430
C	-8.9976970	-6.1391710	-0.4633100
C	-9.7508870	-6.8070820	-1.4351170
C	-9.4725550	-6.5867620	-2.7863780
C	-8.4607070	-5.7026940	-3.1627940
C	-7.7093100	-5.0100500	-2.1950200
C	-6.6555130	-4.0460480	-2.6112530
C	9.0324640	2.4440440	-1.6169450
C	-4.5957080	10.9024860	0.2637270
C	-4.0337710	9.6255770	0.2084130
C	-4.0831670	8.8629940	-0.9740600
C	-4.6920320	9.4383560	-2.1063920
C	-5.2559920	10.7128750	-2.0472470
C	-5.2158420	11.4545850	-0.8611610
C	-3.5116800	7.4918130	-1.0168050
C	5.9243120	-6.3981150	0.7597220
C	-0.7109670	-0.4624170	-1.3120120
C	0.5881450	-0.9046440	-1.0882090
C	1.6756240	-0.2854950	-1.7232950
C	3.3404340	-2.0467180	-1.3145980
C	4.0025790	0.3144540	-1.2639940
C	3.7014200	1.5537040	-0.6652550
C	4.6832930	2.5240460	-0.5229460

C	6.0129150	2.3196440	-0.9595420
C	6.3011020	1.0701080	-1.5574650
C	5.3219450	0.0977900	-1.7080610
C	2.7681580	-2.9997200	-2.1746960
C	3.1235770	-4.3388980	-2.0694180
C	4.0631280	-4.7980720	-1.1166840
C	4.6126480	-3.8282160	-0.2448410
C	4.2538720	-2.4898210	-0.3388600
C	4.3954480	-6.2182260	-1.1337050
C	6.9563630	3.4110640	-0.7663780
C	8.4440950	6.0264020	-0.6580990
C	9.1935260	7.1755780	-0.4092550
C	10.5870530	7.1056980	-0.3029670
C	11.2204040	5.8697110	-0.4534760
C	10.4722660	4.7181480	-0.7017770
C	9.0686450	4.7719550	-0.7978900
C	8.2872710	3.5310800	-1.0521450
C	-6.1351690	-4.2346410	-3.9350230
C	-1.9454690	1.8733270	1.2868100
C	-0.8879090	1.0996090	1.7373880
C	0.3847870	3.0633280	2.1789890
C	-0.6536380	3.8650600	1.7462770
C	-1.8386520	3.2629920	1.2996170
C	1.6809810	-0.2621770	2.0923450
C	-0.4990500	0.0067250	4.6961900
C	-0.5050140	-1.3895790	4.6671250
C	-1.7291030	-2.0553030	4.7193060
C	-2.9147340	-1.3240570	4.7939590
C	-2.8936390	0.0751620	4.8252900
C	-1.6810140	0.7537580	4.7813170
C	-4.2568490	-2.0189360	4.8644420
N	-1.8508390	6.8411050	0.8712710
N	-2.2944130	1.0808380	-2.3446210
N	9.6679840	1.5834090	-2.0710770
N	6.4949870	-5.9657550	1.6752090
N	3.0103090	-0.6749790	-1.4244620
N	-5.7476490	-4.4129340	-5.0160610
N	0.2440460	1.7082780	2.1739600
N	1.2805520	0.9763780	2.7705340

H	6.7139710	-11.1593370	1.0707720
H	6.2669900	-8.7572040	1.4095510
H	4.8257050	-8.4593460	-2.6514280
H	5.2530320	-10.8570980	-2.9767100
H	2.2497510	1.2041240	-3.1818320
H	-0.0624670	1.9799380	-3.6001780
H	-4.3231830	0.9750670	-0.5734950
H	-5.9495250	-0.8600290	-0.3368780
H	-4.1783510	-2.8563450	-3.7368040
H	-2.5293330	-1.0430660	-3.9260600
H	-4.5831690	2.1568920	-3.1981670
H	-5.2427370	4.5147700	-3.0191880
H	-1.3778630	5.4614120	-1.3458590
H	-0.7236890	3.1306150	-1.5211170
H	-4.7194530	6.8135750	-2.5820920
H	-6.7553640	-2.9229640	-0.8641130
H	-7.3856350	-4.7745060	-0.0618470
H	-9.1892180	-6.3188820	0.5972430
H	-10.0460060	-7.1073690	-3.5569630
H	-8.2524340	-5.5408680	-4.2222500
H	-4.5479320	11.4692340	1.1965220
H	-3.5561430	9.2095610	1.0982020
H	-4.7048280	8.8958350	-3.0534660
H	-5.7190060	11.1362070	-2.9417630
H	-1.5331040	-0.9671310	-0.8076610
H	0.7425950	-1.7290190	-0.3921390
H	2.6872170	1.7545410	-0.3187980
H	4.4227700	3.4751360	-0.0510890
H	7.3008790	0.8531360	-1.9272830
H	5.5769170	-0.8481650	-2.1866600
H	2.0509070	-2.6825630	-2.9326790
H	2.6748930	-5.0580940	-2.7592630
H	5.3259370	-4.1166590	0.5240700
H	4.6950400	-1.7680770	0.3502360
H	3.8755800	-6.7704310	-1.9205080
H	6.5074980	4.2894000	-0.2959250
H	7.3622520	6.1148950	-0.7717000
H	8.6849640	8.1377060	-0.3115050
H	12.3079170	5.7974730	-0.3787930

H	10.9827910	3.7604200	-0.8191770
H	-2.8369740	1.3381280	0.9660920
H	-0.9371310	0.0079450	1.7709610
H	1.3347180	3.4249450	2.5665540
H	-0.5599340	4.9528740	1.7498980
H	-2.6643550	3.8918150	0.9657960
H	2.5390840	-0.6528810	2.6497960
H	2.0118380	-0.0043240	1.0798830
H	0.8833740	-1.0180430	2.0423310
H	0.4312030	-1.9445110	4.6056530
H	-1.7672400	-3.1417220	4.6688290
H	-3.8295020	0.6327990	4.8849810
H	-1.6373790	1.8424290	4.8201590
S	1.0403800	0.8747530	4.5191980
F	-4.8957870	-1.6583810	6.0021000
F	-4.1474410	-3.3485160	4.8755490
F	-5.0529650	-1.6631630	3.8498040
F	-3.0922350	-0.8096460	1.6418460
F	-1.0689570	-1.8841750	1.5589420
F	-2.7279230	-2.4314480	0.0610220
F	-2.9436130	-3.0168970	2.2746260
O	2.1526660	0.0529220	4.9713380
O	0.8479770	2.2639960	4.9270220
B	-2.4920110	-2.0668420	1.3808760
H	6.2103700	-12.2418730	-1.1280560
H	-10.5395430	-7.5026310	-1.1394070
H	-5.6527330	12.4545150	-0.8185690
H	11.1721070	8.0083310	-0.1134960

Table S12. Cartesian coordinates of 2D–PATB–CN.

Symbol	X	Y	Z
C	-10.2600980	6.4427580	1.3700310
C	-9.3900050	5.3669360	1.5525160
C	-10.2277950	7.1878100	0.1879330
H	-10.9711180	6.6958540	2.1596750
C	-8.4560710	5.0088800	0.5613170
H	-9.4333750	4.7943050	2.4818420
C	-9.3090560	6.8374520	-0.8088220
H	-10.9120730	8.0262110	0.0409090
C	-8.4393150	5.7626320	-0.6283050

C	-7.5399210	3.8525250	0.7758800
H	-9.2786120	7.3996210	-1.7457270
H	-7.7593480	5.4927170	-1.4382170
C	-7.9402740	2.9434820	1.8109230
C	-6.3936840	3.6482350	0.0591070
N	-8.2883790	2.2312960	2.6603370
C	-5.4132510	2.5686670	0.0730740
H	-6.1580200	4.4317450	-0.6647770
C	-4.3077560	2.6838210	-0.8006120
C	-5.4777560	1.3999140	0.8690900
C	-3.3376300	1.6944590	-0.9050610
H	-4.2210300	3.5729490	-1.4303670
C	-4.5063170	0.4121060	0.7768820
H	-6.2912610	1.2575650	1.5770430
C	-3.4287630	0.5306630	-0.1225860
H	-2.5077290	1.8136030	-1.6023500
H	-4.5845490	-0.4757030	1.4062950
N	-2.4649670	-0.4998900	-0.2331370
C	-1.0934750	-0.1752700	-0.4193890
C	-2.8576450	-1.8539200	-0.1864730
C	-0.3056330	-0.8896640	-1.3370370
C	-0.4918630	0.8516520	0.3255020
C	-2.0304590	-2.8283240	0.4084250
C	-4.0809900	-2.2753840	-0.7426640
C	1.0578370	-0.6308300	-1.4587770
H	-0.7681620	-1.6587860	-1.9584250
C	0.8699480	1.1082590	0.2095310
H	-1.0757300	1.4509810	1.0235860
C	-2.4216150	-4.1592920	0.4386020
H	-1.0732650	-2.5351140	0.8398430
C	-4.4691680	-3.6072230	-0.7033020
H	-4.7319780	-1.5425030	-1.2206610
C	1.6691940	0.3473180	-0.6571140
H	1.6508570	-1.1899500	-2.1837770
H	1.3049930	1.9144790	0.7972980
C	-3.6491530	-4.5970030	-0.1118920
H	-1.7633630	-4.8936660	0.9099640
H	-5.4203580	-3.8781510	-1.1569800
N	3.0701620	0.5943870	-0.7117830

C	-3.9508610	-6.0180250	-0.0292270
C	4.0129480	-0.4330400	-0.6919850
C	3.4803680	1.9520340	-0.5260170
C	-5.0314400	-6.7486670	-0.4369870
H	-3.1635830	-6.5970780	0.4589090
C	5.3608930	-0.1769500	-1.0369240
C	3.6793790	-1.7548240	-0.3138090
C	4.2878840	2.3184430	0.5568250
C	2.9892790	2.9470310	-1.3810080
C	-6.1718640	-6.1244080	-1.0415960
C	-5.1239110	-8.2265090	-0.2835210
C	6.3229850	-1.1683710	-0.9311330
H	5.6448940	0.8182390	-1.3792780
C	4.6519900	-2.7370430	-0.2036970
H	2.6479240	-2.0075130	-0.0807470
C	4.5797080	3.6557640	0.7854710
H	4.6646360	1.5552150	1.2382220
C	3.2918490	4.2847960	-1.1586350
H	2.3455480	2.6632700	-2.2153870
N	-7.1146880	-5.6517400	-1.5310880
C	-3.9777470	-9.0272850	-0.1167730
C	-6.3778480	-8.8647030	-0.3134120
C	6.0136070	-2.4675260	-0.4644820
H	7.3573770	-0.9315250	-1.1961540
H	4.3417970	-3.7354900	0.0985020
C	4.0991980	4.6726600	-0.0673880
H	5.1697060	3.9289780	1.6627270
H	2.8836730	5.0315030	-1.8362010
C	-4.0864150	-10.4099040	0.0367160
H	-2.9849060	-8.5738650	-0.1346100
C	-6.4838000	-10.2480980	-0.1597880
H	-7.2819680	-8.2685300	-0.4538310
C	7.0910860	-3.4252370	-0.2783760
C	4.4603220	6.0459910	0.2637010
C	-5.3401280	-11.0299370	0.0180560
H	-3.1807320	-11.0092500	0.1569490
H	-7.4713550	-10.7158160	-0.1826900
C	7.1675450	-4.4960110	0.5716980
H	7.9931420	-3.2195180	-0.8617290

C	4.3838660	7.1971560	-0.4636730
H	4.8997060	6.1528270	1.2586000
H	-5.4221650	-12.1124710	0.1334610
C	6.1168470	-4.7121400	1.5249470
C	8.3303350	-5.4187970	0.6342770
C	4.8020270	8.5214040	0.0679920
C	3.9277150	7.1949580	-1.8245770
N	5.2767370	-4.8532500	2.3165460
C	8.5799580	-6.1843060	1.7889970
C	9.2173600	-5.5530500	-0.4511230
C	5.1839430	9.5523780	-0.8117870
C	4.8318230	8.7820430	1.4507780
N	3.5771330	7.2324130	-2.9316920
C	9.6876990	-7.0293800	1.8659100
H	7.9008620	-6.1098450	2.6415950
C	10.3261510	-6.3964760	-0.3710190
H	9.0283160	-5.0129260	-1.3802470
C	5.6080770	10.7894720	-0.3245110
H	5.1526360	9.3778770	-1.8888080
C	5.2561600	10.0193300	1.9350670
H	4.4913530	8.0229260	2.1570690
C	10.5730930	-7.1395600	0.7889830
H	9.8594200	-7.6073640	2.7773530
H	10.9959440	-6.4839860	-1.2301130
C	5.6514110	11.0292280	1.0508160
H	5.9052670	11.5701690	-1.0283300
H	5.2629530	10.2007610	3.0118760
H	11.4383630	-7.8033870	0.8470710
H	5.9786300	11.9995170	1.4329410

Table S13. Cartesian coordinates of **2a** in 2D–PATB–CN.

Symbol	X	Y	Z
F	-1.9917080	3.2064830	-1.6805320
B	-1.7612020	1.9195980	-1.2100140
F	-1.3956210	1.9720720	0.1581110
F	-2.8518640	1.0926940	-1.3909180
F	-0.6246110	1.3702530	-1.9146290
H	0.7069630	1.3315860	-0.5480830
C	1.4775870	1.4660610	0.2161660
C	1.2855940	2.2736510	1.3244140

N	2.6494600	0.7962620	0.0742520
C	2.2904820	2.3789010	2.2849490
H	0.3215220	2.7732390	1.4012750
C	3.6576320	0.8956220	0.9851030
N	2.8336340	-0.1416160	-0.9524250
C	3.4957870	1.6850220	2.1079860
H	2.1553700	2.9955170	3.1734030
H	4.5390000	0.2975120	0.7625180
C	2.3933420	0.2627760	-2.2926840
S	2.3448540	-1.7524000	-0.4136700
H	4.2912690	1.7702040	2.8513220
H	2.6802430	-0.5515600	-2.9671860
H	2.9495490	1.1628100	-2.5770100
H	1.3121450	0.4540400	-2.3652750
C	0.6157750	-1.5818390	-0.0469710
O	2.5447040	-2.5827920	-1.5923410
O	3.0760240	-1.9405530	0.8363850
C	-0.3185180	-1.7006010	-1.0791910
C	0.2400480	-1.2523660	1.2617060
C	-1.6642960	-1.4775730	-0.7924070
H	0.0025510	-1.9636490	-2.0869420
C	-1.1059340	-1.0262740	1.5292130
H	0.9941050	-1.1865580	2.0454590
C	-2.0522380	-1.1391350	0.5043360
H	-2.4117590	-1.5344630	-1.5820760
H	-1.4248570	-0.7619530	2.5389070
C	-3.5067740	-0.9033790	0.8473130
F	-3.8917530	-1.7716480	1.8125000
F	-4.3173120	-1.0866730	-0.1969940
F	-3.7078170	0.3249010	1.3366560

Table S14. Cartesian coordinates of 2D–PATB &**2a**

Symbol	X	Y	Z
C	-12.5270220	4.2189900	0.8778800
C	-11.4544360	3.3381090	0.7043450
C	-12.3894730	5.5918660	0.6198040
H	-13.4824370	3.8248290	1.2341670
C	-10.2002330	3.7860700	0.2374860
H	-11.5670320	2.2752730	0.9278710

C	-11.1393130	6.0472390	0.1727270
H	-13.2205220	6.2828640	0.7710810
C	-10.0644620	5.1680420	-0.0188570
N	-9.1600260	2.8399840	0.1231170
H	-10.9878560	7.1123000	-0.0229130
H	-9.0976740	5.5661080	-0.3359760
C	-8.2335210	2.9181410	-0.7587250
C	-7.0916650	1.9813970	-0.8671280
H	-8.2356040	3.7177210	-1.5277270
C	-6.1263240	2.1750990	-1.8761340
C	-6.8968370	0.8841330	-0.0015690
C	-5.0136210	1.3421570	-2.0078740
H	-6.2527110	2.9994710	-2.5839310
C	-5.7836870	0.0519770	-0.1212730
H	-7.6328330	0.7074060	0.7856010
C	-4.8026490	0.2726400	-1.1154670
H	-4.2981400	1.5234590	-2.8109160
H	-5.6527800	-0.7770160	0.5768540
N	-3.6488700	-0.5604600	-1.1877000
C	-2.3426070	-0.0539490	-1.4448730
C	-3.8087100	-1.9776190	-1.0062950
C	-1.2897160	-0.9108890	-1.8243370
C	-1.9943250	1.3065370	-1.3077940
C	-3.1676380	-2.6538890	0.0448380
C	-4.5690000	-2.7348030	-1.9175320
C	0.0213010	-0.4583570	-1.9959390
H	-1.4878280	-1.9718090	-1.9743770
C	-0.6928490	1.7684970	-1.5261500
H	-2.7468350	2.0340450	-1.0052760
C	-3.2378040	-4.0459470	0.1478470
H	-2.5949940	-2.0864280	0.7760520
C	-4.6453600	-4.1245630	-1.8085350
H	-5.0823800	-2.2202590	-2.7325030
C	0.3765030	0.9001320	-1.8518070
H	0.7780790	-1.1961550	-2.2480390
H	-0.5002070	2.8323530	-1.3951330
C	-3.9638600	-4.8156590	-0.7854670
H	-2.7157050	-4.5357530	0.9754560
H	-5.2169000	-4.7075340	-2.5334890

N	1.7046940	1.3853180	-1.9678000
C	-3.9734810	-6.2939560	-0.6809060
C	2.8219410	0.5792000	-2.3229170
C	1.9464140	2.7878750	-1.6922360
N	-4.5499080	-7.0615810	-1.5285020
H	-3.4094040	-6.6970630	0.1845110
C	2.7661990	-0.3791600	-3.3564600
C	4.0531880	0.7195490	-1.6386150
C	2.0544470	3.2671560	-0.3826470
C	2.0390460	3.6953570	-2.7627900
C	-4.5776760	-8.4665450	-1.4204030
C	3.8591170	-1.2023740	-3.6357890
H	1.8634660	-0.4704560	-3.9601370
C	5.1428080	-0.1020070	-1.9303430
H	4.1345540	1.4423390	-0.8264360
C	2.2433180	4.6345860	-0.1491160
H	1.9947660	2.5818620	0.4611120
C	2.2093380	5.0592570	-2.5269950
H	1.9587830	3.3179710	-3.7845750
C	-4.5963740	-9.1864920	-0.2064100
C	-4.6482880	-9.2101720	-2.6172220
C	5.0712020	-1.1049410	-2.9215070
H	3.7731560	-1.9342850	-4.4445880
H	6.0715010	0.0090050	-1.3662510
C	2.3048780	5.5579130	-1.2110950
H	2.3523320	4.9672560	0.8855580
H	2.2623670	5.7683250	-3.3553610
C	-4.6677290	-10.5856320	-0.2023800
H	-4.5910040	-8.6473560	0.7437930
C	-4.7028050	-10.6071280	-2.6057870
H	-4.6581770	-8.6600090	-3.5602700
C	6.1826070	-2.0295300	-3.2420990
C	2.4450400	7.0081560	-0.9299170
C	-4.7174090	-11.3197430	-1.3976560
H	-4.6954410	-11.1087590	0.7572180
H	-4.7488050	-11.1444640	-3.5565700
N	7.2920740	-2.0856900	-2.6025550
H	5.9833410	-2.6892290	-4.1120230
N	2.4353720	7.9066380	-1.8422450

H	2.5293980	7.2638140	0.1451350
H	-4.7772510	-12.4094620	-1.3854300
C	8.3164520	-3.0093990	-2.9017680
C	2.5865950	9.2846480	-1.5895550
C	9.6467810	-2.6263610	-2.6285500
C	8.1115580	-4.3148220	-3.4000520
C	3.3736840	9.8387870	-0.5574390
C	1.9489150	10.1817450	-2.4722710
C	10.7205400	-3.4901670	-2.8689700
H	9.8174990	-1.6272840	-2.2226340
C	9.1910070	-5.1804630	-3.6237880
H	7.0951540	-4.6709790	-3.5847920
C	3.4989810	11.2272240	-0.4168750
H	3.9198470	9.1789540	0.1207040
C	2.0651160	11.5660460	-2.3158910
H	1.3562430	9.7608850	-3.2870030
C	10.5136880	-4.7856340	-3.3677190
H	11.7346960	-3.1462680	-2.6497040
H	8.9883200	-6.1877510	-3.9977800
C	2.8462720	12.1136790	-1.2875810
H	4.1285180	11.6201340	0.3860380
H	1.5474140	12.2251040	-3.0178000
H	11.3489720	-5.4662430	-3.5408690
H	2.9524540	13.1939210	-1.1728900
F	3.8808470	1.1533880	1.5413610
B	3.3164440	1.8410010	2.6472450
F	3.9078380	1.4209850	3.8256290
F	1.9175600	1.4602590	2.6744380
F	3.3953220	3.2061070	2.4627580
H	1.9267260	-0.1400140	1.6891880
C	1.9446080	-1.0332490	1.0563080
C	3.0962430	-1.4668680	0.4183330
N	0.7902280	-1.7260880	0.8926290
C	3.0537590	-2.6095690	-0.3801990
H	3.9984730	-0.8822830	0.5807280
C	0.7094080	-2.8321980	0.1090110
N	-0.3779880	-1.3528070	1.5632290
C	1.8429710	-3.2969000	-0.5312200
H	3.9549160	-2.9499140	-0.8927620

H	-0.2764800	-3.2853080	0.0389440
C	-0.6464650	0.0891250	1.6515020
S	-0.5912780	-2.2826610	3.0384080
H	1.7655290	-4.1851570	-1.1586740
H	-1.6806640	0.1948230	1.9949980
H	-0.5700700	0.5001950	0.6402840
H	0.0422730	0.6270750	2.3164430
C	0.8175980	-1.8290170	4.0149280
O	-1.8224630	-1.7472320	3.6012840
O	-0.4557540	-3.6762690	2.6132370
C	0.7848020	-0.6374730	4.7446500
C	1.9744900	-2.6133500	3.9239790
C	1.9461730	-0.2145710	5.3883770
H	-0.1307140	-0.0485810	4.7986160
C	3.1228000	-2.1819230	4.5807250
H	1.9607880	-3.5436600	3.3554930
C	3.1073090	-0.9835180	5.3046170
H	1.9604030	0.7281810	5.9331110
H	4.0385350	-2.7728020	4.5257790
C	4.3684230	-0.5679360	6.0305740
F	4.6074590	-1.4290480	7.0469760
F	4.2789860	0.6528090	6.5580230
F	5.4359680	-0.6147620	5.2265890

Table S15. Cartesian coordinates of 2D–PATB

Symbol	X	Y	Z
C	11.4949360	-4.6629110	0.3995200
C	10.4734620	-3.7079170	0.4324510
C	11.2249570	-6.0061040	0.0941410
H	12.5171960	-4.3516740	0.6302500
C	9.1365390	-4.0473390	0.1339900
H	10.6919270	-2.6698990	0.6913990
C	9.8943550	-6.3543760	-0.1857640
H	12.0182310	-6.7555260	0.0848000
C	8.8679220	-5.3998100	-0.1704100
N	8.1601680	-3.0330400	0.2239250
H	9.6414020	-7.3939020	-0.4115310
H	7.8398130	-5.7166330	-0.3609740
C	7.1129190	-2.9990120	-0.5138250
C	6.0364210	-1.9880520	-0.4034260

H	6.9475190	-3.7484310	-1.3148930
C	4.9243270	-2.0588960	-1.2669790
C	6.0465410	-0.9360090	0.5369160
C	3.8659030	-1.1517230	-1.1893040
H	4.8886980	-2.8445160	-2.0272890
C	4.9893570	-0.0303310	0.6265040
H	6.8990570	-0.8541610	1.2144680
C	3.8620030	-0.1279640	-0.2214880
H	3.0310220	-1.2384330	-1.8858060
H	5.0194560	0.7607690	1.3781710
N	2.7716360	0.7782710	-0.0789510
C	1.4094540	0.3708900	-0.1656330
C	3.0556800	2.1688960	0.1496910
C	0.3760110	1.3134550	-0.3402350
C	0.9893810	-0.9710950	-0.0488990
C	2.6223970	2.8179690	1.3176660
C	3.7280720	2.9317610	-0.8237800
C	-0.9747030	0.9548870	-0.3389150
H	0.6248340	2.3671760	-0.4629940
C	-0.3593280	-1.3367780	-0.0956540
H	1.7243840	-1.7612810	0.1008770
C	2.8048450	4.1939920	1.4808670
H	2.1226350	2.2434620	2.0953460
C	3.9173360	4.3047410	-0.6557020
H	4.0808270	2.4372480	-1.7312570
C	-1.4004460	-0.3851110	-0.2140870
H	-1.7059150	1.7524940	-0.4377330
H	-0.6057050	-2.3925970	0.0073550
C	3.4411480	4.9728260	0.4913080
H	2.4435620	4.6636290	2.4008390
H	4.4180080	4.8943830	-1.4262350
N	-2.7623920	-0.7785520	-0.1569890
C	3.5701530	6.4384340	0.6694600
C	-3.8594310	0.1159000	-0.3006380
C	-3.0602730	-2.1775910	0.0785630
N	4.0709930	7.2192480	-0.2133890
H	3.1667690	6.8219980	1.6285140
C	-3.8858730	1.1305820	-1.2801750
C	-4.9858410	0.0107870	0.5502960

C	-3.0117660	-2.7287250	1.3632050
C	-3.3702970	-3.0100790	-1.0118680
C	4.2132400	8.6104570	-0.0382160
C	-4.9467550	2.0358950	-1.3516950
H	-3.0755390	1.2023500	-2.0053610
C	-6.0453740	0.9150040	0.4659560
H	-4.9999960	-0.7540370	1.3271630
C	-3.2610700	-4.0934840	1.5512300
H	-2.7826890	-2.1015930	2.2230580
C	-3.6006870	-4.3726180	-0.8241750
H	-3.4118770	-2.5763930	-2.0134140
C	4.1627440	9.4203290	-1.1922960
C	4.4577430	9.2519270	1.1951480
C	-6.0468940	1.9704980	-0.4717750
H	-4.9269330	2.8093120	-2.1254340
H	-6.8884370	0.8276450	1.1548740
C	-3.5403070	-4.9442190	0.4638490
H	-3.2428490	-4.4816250	2.5720720
H	-3.8226560	-5.0247930	-1.6710390
C	4.3168770	10.8078300	-1.1169650
H	3.9975490	8.9295720	-2.1535370
C	4.6275810	10.6410080	1.2607840
H	4.5514880	8.6568400	2.1065570
C	-7.1248170	2.9804830	-0.5760760
C	-3.7405080	-6.3974350	0.6874430
C	4.5559760	11.4426110	0.1108370
H	4.2630230	11.3986270	-2.0350570
H	4.8303670	11.1014740	2.2314060
N	-8.1236080	3.0662850	0.2227620
H	-7.0070840	3.6786580	-1.4306910
N	-3.9260910	-7.2370480	-0.2614060
H	-3.6866840	-6.7128750	1.7484870
H	4.6937770	12.5236720	0.1703010
C	-9.1126110	4.0686580	0.1266410
C	-4.1362180	-8.6158690	-0.0605800
C	-10.4132840	3.7532050	0.5737240
C	-8.8900800	5.3869080	-0.3284350
C	-3.6975770	-9.4950530	-1.0731190
C	-4.8032750	-9.1822210	1.0467240

C	-11.4467730	4.6953700	0.5398900
H	-10.5937740	2.7436690	0.9483270
C	-9.9266480	6.3302310	-0.3448540
H	-7.8883840	5.6897110	-0.6430310
C	-3.8874810	-10.8764090	-0.9729230
H	-3.2004540	-9.0627040	-1.9438170
C	-5.0047310	-10.5661590	1.1324340
H	-5.1978700	-8.5325310	1.8314210
C	-11.2230790	6.0036090	0.0835510
H	-12.4404010	4.4025180	0.8888840
H	-9.7094160	7.3439940	-0.6922390
C	-4.5487720	-11.4364160	0.1300800
H	-3.5243520	-11.5220940	-1.7768360
H	-5.5379720	-10.9676420	1.9983610
H	-12.0244240	6.7442900	0.0713800
H	-4.7133260	-12.5129430	0.2033150

Table S16. Cartesian coordinates of **2a** in 2D–PATB.

Symbol	X	Y	Z
F	-1.0537080	2.9801260	0.0371680
B	-1.6794780	2.3036480	-1.0422450
F	-2.6358790	1.4238260	-0.5668910
F	-0.6306780	1.5374150	-1.6868270
F	-2.1856410	3.2068930	-1.9542300
H	0.6608280	1.4177120	-0.3269160
C	1.4261810	1.4179460	0.4560020
C	1.2674130	2.1077730	1.6477370
N	2.5687710	0.7212750	0.2356650
C	2.2818100	2.0744820	2.6043540
H	0.3344170	2.6496390	1.7820160
C	3.5831840	0.6816800	1.1375890
N	2.7428370	-0.0318660	-0.9289170
C	3.4505930	1.3494810	2.3406140
H	2.1664950	2.6237100	3.5401370
H	4.4565560	0.1090620	0.8350600
C	2.2561040	0.5741140	-2.1758820
S	2.3329010	-1.7195040	-0.6641470
H	4.2721770	1.3114660	3.0563310
H	2.6635140	-0.0262130	-2.9958530
H	2.6772450	1.5821790	-2.2376940

H	1.1614500	0.6319560	-2.2413360
C	0.6088360	-1.6618780	-0.2535450
O	2.5482820	-2.3290670	-1.9685140
O	3.0997450	-2.0985870	0.5230880
C	-0.3335060	-1.5798840	-1.2824360
C	0.2458740	-1.5684190	1.0960370
C	-1.6726920	-1.3852830	-0.9496820
H	-0.0214920	-1.6574680	-2.3238310
C	-1.0967010	-1.3840320	1.4115590
H	1.0062920	-1.6485760	1.8733280
C	-2.0494260	-1.2872880	0.3900940
H	-2.4235650	-1.2804390	-1.7312930
H	-1.4074010	-1.3066990	2.4547330
C	-3.5024610	-1.1196350	0.7783090
F	-3.9425990	-2.2517690	1.3751980
F	-4.2917260	-0.8923470	-0.2713670
F	-3.6633550	-0.1298640	1.6630140

Table S17. Cartesian coordinates of 3D–PATB &**2a**

Symbol	X	Y	Z
C	7.7302970	-4.9225020	-1.5442010
C	7.1557970	-4.1008020	-2.5372010
C	5.7825970	-4.1022020	-2.7469010
C	4.9169970	-4.9189030	-1.9933010
C	5.4961970	-5.7452020	-1.0057010
C	6.8661970	-5.7447020	-0.7848010
C	2.6416970	-5.4925030	-1.6747010
C	-1.2368040	-1.0362040	-0.7498010
C	-7.3882030	-5.3443060	0.2958990
C	-6.1216030	-4.7484060	-0.1296010
C	-5.7358040	-3.4605050	0.2897990
C	-3.6662030	-3.6233050	-0.9723010
C	-5.2526030	-5.4585050	-0.9766010
C	-4.5294040	-2.9082050	-0.1156010
C	-4.0482030	-4.9075050	-1.4018010
C	-2.2850040	-1.6474050	-1.4536010
C	-3.1610040	-0.8366050	-2.1906010
C	-1.2908030	-3.8446040	-1.5779010
C	-0.9291030	-4.8586040	-0.6722010
C	0.3171970	-5.4757040	-0.7748010

C	1.2352970	-5.0961040	-1.7711010
C	0.8130970	-4.1663040	-2.7439010
C	-0.4225030	-3.5505040	-2.6497010
C	-11.9083030	-6.4000070	2.3127990
C	-11.2186030	-5.4487070	3.0894990
C	-10.0188030	-4.9058070	2.6434990
C	-9.4379030	-5.3063060	1.4253990
C	-10.1480030	-6.2350070	0.6365990
C	-11.3569030	-6.7668070	1.0682990
C	7.8298940	6.1155980	1.2408990
C	7.4282940	4.8113980	0.8925990
C	6.1193940	4.5436980	0.5097990
C	5.1564940	5.5727980	0.4685990
C	5.5479940	6.8667980	0.8565990
C	6.8626940	7.1408980	1.2137990
C	3.5143940	4.4897970	-0.7686010
C	-1.9646050	1.1644960	-1.4831010
C	-1.0763050	0.3453960	-0.7625010
C	-0.4978050	3.1051960	-1.3276010
C	0.4537950	2.9143960	-2.3385010
C	1.7524950	3.3961970	-2.1702010
C	2.1237940	4.0780970	-0.9978010
C	1.1550940	4.2797960	0.0050990
C	-0.1399060	3.8022960	-0.1615010
C	-6.2410060	6.1316940	-1.0544010
C	-5.1118060	5.2074950	-1.1591010
C	-5.1865060	3.9022950	-0.6344010
C	-2.9191050	3.4312950	-1.3694010
C	-3.9148060	5.6059950	-1.7781010
C	-4.1119050	3.0316950	-0.7295010
C	-2.8348060	4.7354950	-1.8890010
C	-3.0026050	0.5462950	-2.2087010
C	-10.6577070	8.3952930	0.0088990
C	-10.8203060	7.0040930	-0.1390010
C	-9.7168060	6.1756930	-0.3045010
C	-8.4095060	6.6948940	-0.3638010
C	-8.2509070	8.0870940	-0.2065010
C	-9.3498070	8.9184940	-0.0216010
C	2.4967950	0.5652970	-0.5722010

C	2.3982960	-0.1428030	0.6143990
C	2.4287960	-2.1987030	-0.5869010
C	2.5704960	-1.5238030	-1.7827010
C	2.5847960	-0.1249030	-1.7793010
C	0.9814960	-2.7828040	2.0945990
C	4.9647960	-2.0644030	1.6753990
C	5.1012960	-0.8542020	2.3616990
C	6.0745960	0.0473980	1.9299990
C	6.8961960	-0.2910020	0.8492990
C	6.7598960	-1.5182020	0.1890990
C	5.7776960	-2.4187020	0.5948990
C	7.8993950	0.7168980	0.3418990
N	9.0899970	-4.9410010	-1.3347010
N	3.5487970	-4.7649030	-2.2187010
N	-8.2111030	-4.7413060	1.0695990
N	-2.4487040	-3.0619050	-1.4034010
N	-13.1307030	-6.9330080	2.7298990
N	9.1514940	6.3891990	1.5777990
N	3.8245940	5.3488970	0.1228990
N	-1.8119050	2.5634960	-1.4666010
N	-7.3579060	5.7919940	-0.5285010
N	-11.7624070	9.2403930	0.1535990
N	2.3427960	-1.4950030	0.5711990
N	2.2721960	-2.1506030	1.8122990
H	9.4368970	-5.3383010	-0.4712010
H	9.6442970	-4.1821010	-1.7094010
H	7.7998960	-3.4558020	-3.1407010
H	5.3393960	-3.4513020	-3.5032010
H	4.8638970	-6.3552030	-0.3605010
H	7.2862970	-6.3764020	0.0022990
H	2.8673970	-6.3548030	-1.0228010
H	-0.5565040	-1.6585040	-0.1682010
H	-7.5817030	-6.3657060	-0.0874010
H	-6.4079040	-2.9141060	0.9537990
H	-5.5370030	-6.4567050	-1.3214010
H	-4.2411040	-1.9145050	0.2288990
H	-3.3973030	-5.4655050	-2.0765010
H	-3.9716040	-1.3010050	-2.7553010
H	-1.6078030	-5.1202040	0.1409990

H	0.6172970	-6.2091040	-0.0213010
H	1.4984970	-3.9213030	-3.5563010
H	-0.7291040	-2.8031040	-3.3833010
H	-11.6359030	-5.1328070	4.0494990
H	-9.4855030	-4.1669060	3.2448990
H	-9.7609030	-6.5258070	-0.3422010
H	-11.8965020	-7.4738070	0.4318990
H	9.2958940	7.2127990	2.1514990
H	9.6898940	5.5945990	1.9047990
H	8.1489940	3.9922980	0.9509990
H	5.8117950	3.5173980	0.3128990
H	4.7936930	7.6560970	0.8605990
H	7.1506930	8.1580980	1.4943990
H	4.2800940	3.9858970	-1.3910010
H	-0.2776050	0.8084960	-0.1849010
H	0.1636950	2.3832960	-3.2475010
H	2.4967950	3.2416970	-2.9573010
H	1.4718940	4.7596970	0.9313990
H	-0.8849060	3.9366960	0.6251990
H	-6.0682060	7.1442940	-1.4698010
H	-6.1095050	3.6020940	-0.1354010
H	-3.8333060	6.6162950	-2.1889010
H	-4.1829050	2.0339950	-0.2954010
H	-1.9153060	5.0592960	-2.3780010
H	-3.6887050	1.1619950	-2.7915010
H	-11.8253060	6.5740930	-0.1107010
H	-9.8415060	5.0952930	-0.4010010
H	-7.2491070	8.5213940	-0.1907010
H	-9.1992070	9.9928940	0.1164990
H	2.5445950	1.6487970	-0.5117010
H	2.4055950	0.3221970	1.6076990
H	2.4177960	-3.2813030	-0.5058010
H	2.6806960	-2.1174030	-2.6880010
H	2.6779950	0.4305970	-2.7143010
H	1.0755960	-3.2735040	3.0716990
H	0.6873960	-3.5223040	1.3307990
H	0.2173960	-1.9967040	2.1727990
H	4.4303960	-0.5755030	3.1748990
H	6.1506950	1.0195980	2.4197990

H	7.4093960	-1.7734020	-0.6482010
H	5.6399960	-3.3700020	0.0838990
B	3.7557950	2.4468970	2.4856990
F	4.0486950	2.1313970	1.1221990
F	3.0662950	1.3086970	3.0276990
F	2.9404950	3.5548970	2.5476990
F	4.9561950	2.6137970	3.1681990
F	8.9146950	0.1209990	-0.3138010
F	8.4307950	1.4408980	1.3376990
F	7.3296950	1.5788980	-0.5199010
S	3.6632960	-3.1800030	2.1419990
O	3.5907960	-3.4010030	3.5787990
O	3.6384970	-4.3240030	1.2196990
H	-13.3241030	-6.8834080	3.7232990
H	-13.3939020	-7.8204080	2.3177990
H	-11.5767070	10.1408930	0.5799990
H	-12.6073070	8.8066930	0.5077990

Table S18. Cartesian coordinates of 3D–PATB.

Symbol	X	Y	Z
H	2.1335680	-1.0841690	3.8446540
C	1.8639690	-0.3396150	3.0991810
C	2.5049410	-0.3935830	1.8775230
C	0.8952580	0.6458250	3.3180880
N	2.1577940	0.4848210	0.9018840
H	3.2595740	-1.1321700	1.6276750
C	0.5622430	1.5292440	2.2937030
H	0.3878320	0.7197580	4.2819670
C	1.1940070	1.4212540	1.0661560
N	2.7370710	0.4109870	-0.3765700
H	-0.2117210	2.2846470	2.4000340
H	0.9321320	2.0234430	0.1868860
C	4.1239130	0.8752180	-0.4456350
S	2.4539390	-1.1270220	-1.1871170
H	4.4530860	0.7389520	-1.4832000
H	4.7962770	0.3281750	0.2366500
H	4.1451360	1.9497030	-0.2129610
C	0.7150890	-1.3066520	-0.8663600
O	2.7481600	-0.8366770	-2.5828710
O	3.1980360	-2.1802740	-0.4825420

C	-0.1695730	-0.3438370	-1.3612580
C	0.3005360	-2.3855490	-0.0805350
C	-1.5209090	-0.4542950	-1.0314220
H	0.1779130	0.5141180	-1.9371580
C	-1.0549650	-2.4939300	0.2224990
H	1.0202310	-3.1140870	0.2880270
C	-1.9529800	-1.5265730	-0.2437780
H	-2.2120250	0.3240950	-1.3616840
H	-1.4037660	-3.3280210	0.8303020
C	-3.4034120	-1.6007190	0.1683770
F	-3.6356860	-0.8611220	1.2678730
F	-3.7733580	-2.8637600	0.4610820
F	-4.2228190	-1.1528690	-0.7945440
F	-0.1336750	2.6935130	-1.1700340
B	-1.4483310	2.9042910	-0.6295150
F	-1.5302520	2.1101070	0.5573980
F	-1.6118000	4.2359290	-0.3174540
F	-2.3904020	2.4371670	-1.5390820

Table S19. Cartesian coordinates of **2a** in 3D–PATB.

Symbol	X	Y	Z
C	9.3597070	-5.7830010	-0.1574650
C	8.3843310	-6.5311910	0.5404700
C	8.9239480	-4.9461180	-1.2067270
N	10.6940990	-5.8850970	0.1628150
C	7.0397680	-6.4466510	0.2076860
H	8.6963220	-7.1717630	1.3688770
C	7.5754450	-4.8621530	-1.5287810
H	9.6562120	-4.3570000	-1.7655710
H	10.9428220	-6.2858740	1.0583750
H	11.3258180	-5.1716350	-0.1759730
C	6.5988170	-5.6053270	-0.8360690
H	6.3183650	-7.0007620	0.8088340
H	7.2384060	-4.1994250	-2.3285740
N	5.2668920	-5.3673800	-1.1760270
C	4.2724130	-6.0234380	-0.6973110
C	2.9073390	-5.5378320	-0.9146460
H	4.3860440	-6.8845800	-0.0159180
C	1.8884690	-5.8366390	0.0082620
C	2.6283080	-4.6026740	-1.9331410

C	0.6820290	-5.1379030	-0.0009080
H	2.0770090	-6.5718500	0.7954860
C	1.4334690	-3.9058370	-1.9503600
H	3.3925770	-4.4196570	-2.6896880
C	0.4628290	-4.1214580	-0.9489930
H	-0.0764230	-5.3373170	0.7574550
H	1.2371990	-3.1557690	-2.7174470
N	-0.6508840	-3.2613440	-0.8817240
C	-1.9333980	-3.7329640	-0.5436370
C	-0.3895380	-1.8613650	-0.9407200
C	-2.8134000	-2.9457970	0.2275270
C	-2.3639490	-4.9990130	-0.9838580
C	0.6363290	-1.3053980	-0.1624280
C	-1.1471610	-1.0115170	-1.7595050
C	-4.0833460	-3.4096060	0.5413860
H	-2.4888050	-1.9657130	0.5797750
C	-3.6333110	-5.4605640	-0.6509320
H	-1.6989920	-5.6120140	-1.5944740
C	0.8892710	0.0616620	-0.1822860
H	1.2250810	-1.9582060	0.4830020
C	-0.8965830	0.3576090	-1.7856200
H	-1.9388910	-1.4333470	-2.3810640
C	-4.5188840	-4.6781810	0.1108470
H	-4.7690940	-2.8074390	1.1394990
H	-3.9543420	-6.4458760	-1.0017780
C	0.1180230	0.9216660	-0.9863330
H	1.6669540	0.4836090	0.4511740
H	-1.4907070	1.0047570	-2.4313690
C	-5.8520520	-5.1816360	0.4404620
N	0.3619400	2.3077830	-0.9792220
H	-6.0819500	-6.1965550	0.0584990
N	-6.6930840	-4.5105050	1.1349130
C	1.6935020	2.7659260	-0.7410440
C	-0.6891660	3.2477890	-0.9857940
C	-7.9796550	-4.9870380	1.3965060
C	2.7094190	2.4925430	-1.6673700
C	2.0014780	3.4621270	0.4396270
C	-1.9538780	2.9405310	-0.4409410
C	-0.4767200	4.5335290	-1.5170390

C	-8.6294740	-4.5249920	2.5563910
C	-8.6837330	-5.8839860	0.5671420
H	2.4592990	1.9628460	-2.5888920
C	4.0186490	2.8924870	-1.3996930
C	3.3076210	3.8582340	0.7051250
H	1.2058350	3.6615450	1.1606000
C	-2.9728650	3.8804100	-0.4469080
H	-2.1260920	1.9584720	0.0019720
C	-1.5018940	5.4740430	-1.5084690
H	0.4992950	4.7858230	-1.9336840
C	-9.8950990	-4.9794510	2.9105480
H	-8.0989990	-3.8099350	3.1887700
C	-9.9563620	-6.3271940	0.9054440
H	-8.2389480	-6.2197250	-0.3721850
C	4.3390650	3.5729360	-0.2116640
H	4.8130290	2.6741130	-2.1203760
H	3.5798230	4.3356460	1.6465870
C	-2.7689650	5.1666310	-0.9838740
H	-3.9511430	3.6514640	-0.0208960
H	-1.3198850	6.4678550	-1.9267460
C	-10.5807270	-5.8994650	2.0952040
H	-10.3665180	-4.6172120	3.8280950
H	-10.4889170	-7.0105740	0.2381190
C	5.7302740	3.8983000	0.1244330
C	-3.8387250	6.1650800	-0.9861510
N	-11.8662120	-6.3430500	2.4184200
H	6.5088970	3.3327890	-0.4247470
N	6.0244710	4.7535150	1.0257220
H	-3.5656790	7.1548650	-1.4019640
N	-5.0147810	5.9094040	-0.5485600
H	-12.1352290	-6.2600780	3.3913240
H	-12.1534030	-7.2193950	1.9998230
C	7.3350260	4.8975740	1.4764380
C	-6.0142060	6.8821160	-0.4853800
C	8.2209600	3.8091840	1.6121560
C	7.7795220	6.1710830	1.8754900
C	-7.3533090	6.4505590	-0.5260280
C	-5.7762850	8.2637960	-0.3361660
C	9.5095010	3.9983400	2.0972770

H	7.8632910	2.8004740	1.4063340
C	9.0766000	6.3662940	2.3350880
H	7.0818300	7.0073690	1.8054570
C	-8.4086030	7.3518040	-0.4638970
H	-7.5407560	5.3781600	-0.6153410
C	-6.8287930	9.1691440	-0.2558310
H	-4.7521490	8.6319200	-0.2455350
C	9.9679120	5.2813460	2.4571220
H	10.1673590	3.1355520	2.2268410
H	9.4079880	7.3679800	2.6222110
C	-8.1669560	8.7320840	-0.3243860
H	-9.4389390	6.9887550	-0.5119860
H	-6.6184420	10.2336920	-0.1213420
N	11.2725100	5.4747550	2.8972990
N	-9.2204130	9.6499210	-0.2835240
H	11.7292520	4.6543100	3.2795860
H	11.4251230	6.2992520	3.4676010
H	-9.0109960	10.5455960	0.1424000
H	-10.1182260	9.2802130	0.0064380

S9 Recycle experiments and catalyst characterization

S9.1 Recycle experiments for C–H pyridylation of 2a with cyclohexane

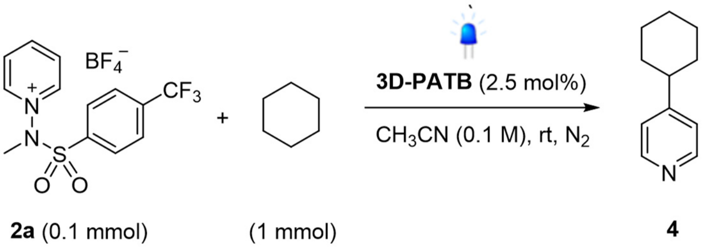

2a (40.4 mg, 0.1 mmol), cyclohexane (220 uL, 1 mmol), and 3D–PATB (1.6 mg, 2.5 μmol, 2.5 mol% catalyst loading based on the PATB linker) were mixed in anhydrous acetonitrile (1.0 mL) in a sealed test tube. The resultant mixture was stirred under blue LED irradiation (440-450 nm) at room temperature in a N_2 atmosphere for 48 hours. After the reaction, the 3D–PATB catalyst was recovered via centrifugation, washed with MeCN (1.0 mL × 3), and used direct in subsequent cycles of reactions. The reaction supernatant was then subjected to ^{1}H NMR analysis. This

procedure was repeated three times.

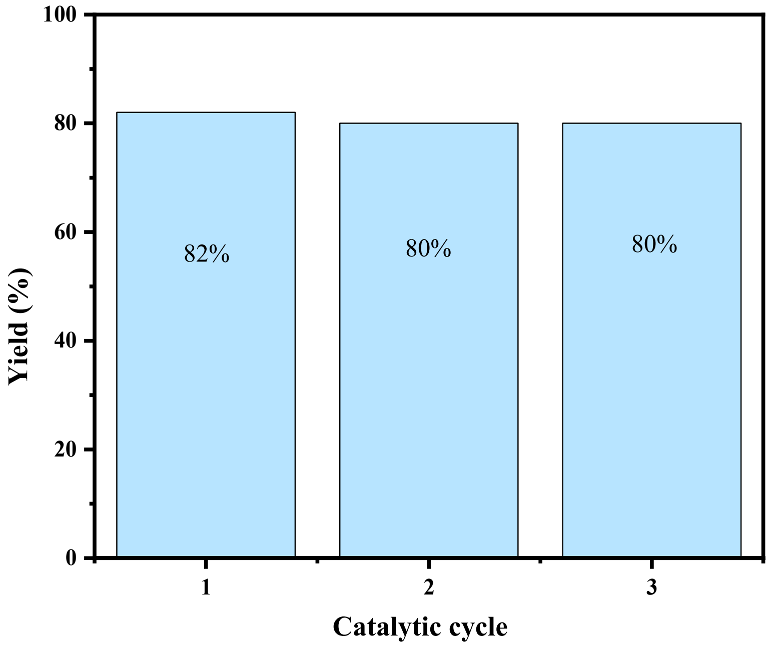

Figure S45. Yields of **4** with recovered 3D–PATB in three consecutive runs.

S9.2 Recycle experiments for C–H pyridylation of 3a with THF

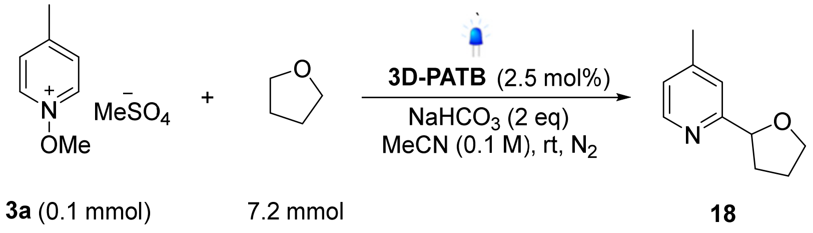

3a (23.6 mg, 0.1 mmol), THF (0.62 mL, 7.6 mmol), $NaHCO_3$ (16.8 mg, 0.2 mmol), and 3D–PATB (1.6 mg, 2.5 μmol, 2.5 mol% catalyst loading based on the PATB linker) were mixed in acetonitrile (1.0 mL) in a sealed test tube. The resultant mixture was stirred under blue LED irradiation (440-450 nm) at room temperature in a N_2 atmosphere for 24 hours. After the reaction, the 3D–PATB catalyst was recovered via centrifugation, washed with MeCN (1.0 mL × 3), and used direct in subsequent cycles of reactions. The reaction supernatant was then subjected to ^1H NMR analysis. This procedure was repeated three times.

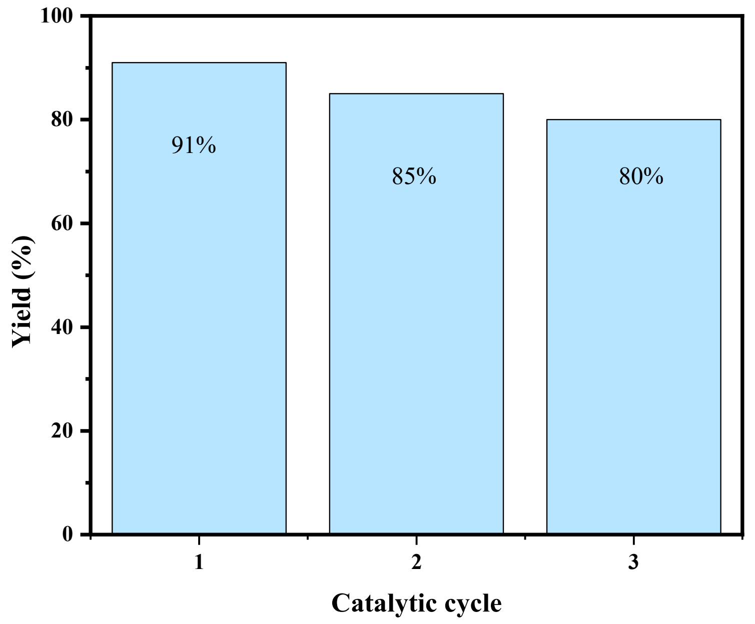

Figure S46. Yields of **18** with the recovered 3D–PATB in three consecutive runs.

S9.3 Recycle experiments for formal C–H alkylation of 23a with 24a

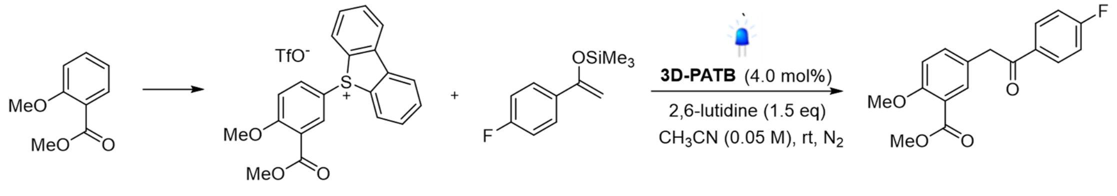

23a (0.05 mmol, 24.9 mg), **24a** (0.25 mmol, 52.6 mg), 2,6-lutidine (0.075 mmol, 8.1 mg), 3D–PATB (1.3 mg, 2.0 μmol, 4.0 mol % catalyst loading based on the PATB linker) were mixed in anhydrous acetonitrile (MeCN, 1.0 mL) in a sealed test tube. The resulting mixture was stirred under blue LED irradiation (390-400 nm) at room temperature in a N_2 atmosphere for 48 hours. After the reaction, the 3D–PATB catalyst was recovered via centrifugation, washed with MeCN (1.0 mL × 3), and used direct in subsequent cycles of reactions. The reaction supernatant was then subjected to ^{1}H NMR analysis. This procedure was repeated three times. The decreased yield is probably attributed to the complexity of the reaction system, which hindered the complete recovery of the catalyst.

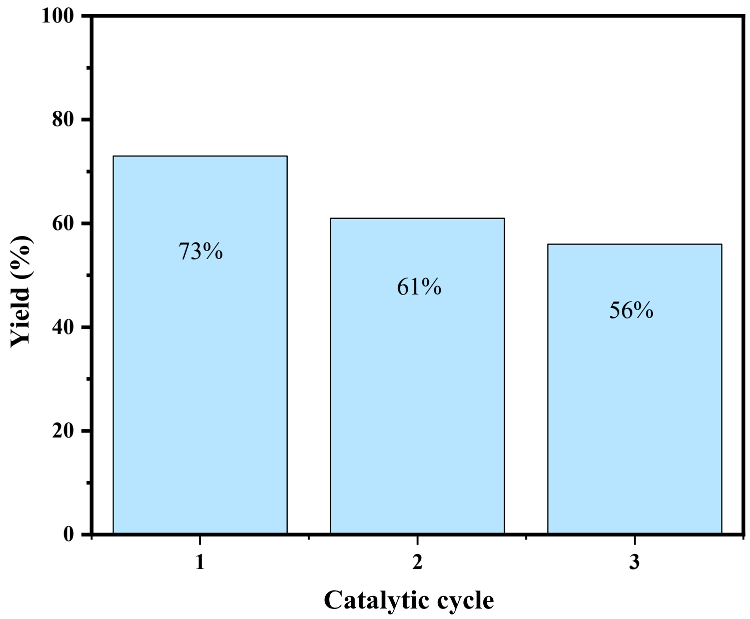

Figure S47. Yields of **25** with recovered 3D–PATB in three consecutive runs.

S9.4 Recycle experiments for [3+2] cyclization of 35a with 36d

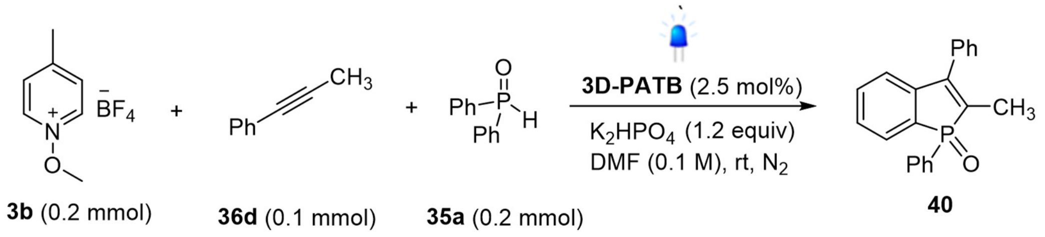

3b (0.2 mmol, 42.2 mg), **36d** (0.1 mmol, 11.6 mg), **35a** (0.2 mmol, 40.4 mg), potassium phosphate dibasic (0.2 mmol, 34.8 mg), 3D–PATB (2.5 mol% catalyst loading based on the PATB linker) were mixed in anhydrous *N,N*-Dimethylformamide (DMF, 1.0 mL) in a sealed test tube. The resulting mixture was stirred under blue LED irradiation (440-450 nm) at room temperature in a N_2 atmosphere for 48 hours. After the reaction, the 3D-PATB catalyst was recovered via centrifugation, washed with DMF (1.0 mL × 3), and used direct in subsequent cycles of reactions. The reaction supernatant was then subjected to ^{1}H NMR analysis. This procedure was repeated three times. The decreased yield is probably attributed to the complexity of the reaction system, which hindered the complete recovery of the catalyst.

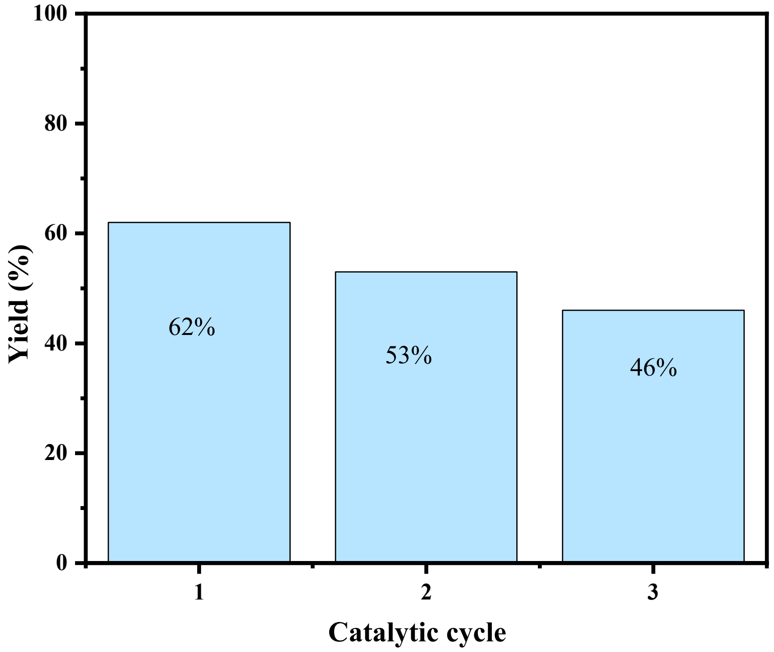

Figure S48. Yields of **40** with recovered 3D-PATB in three consecutive runs.

S9.5 Characterization of the recovered catalyst from C–H pyridylation of 2a with cyclohexane

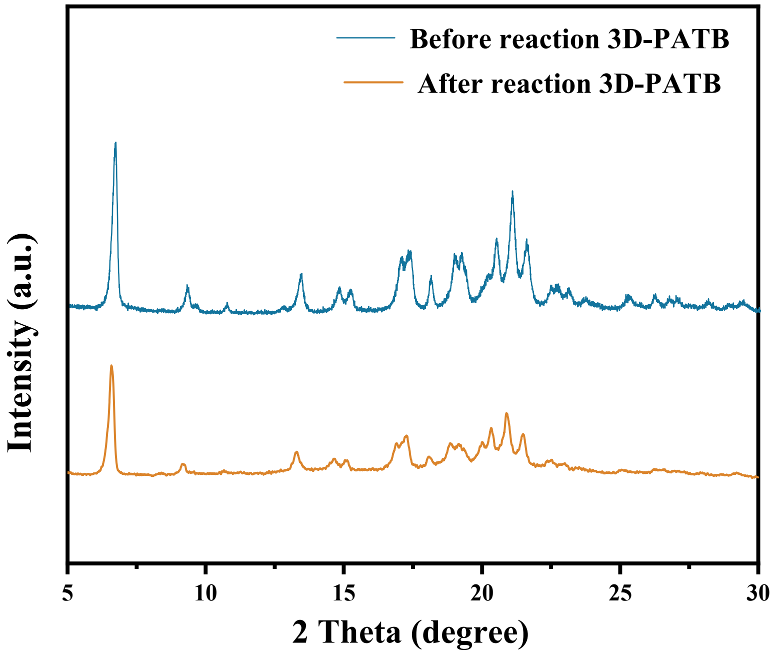

Figure S49. PXRD pattern of 3D–PATB before and after reaction.

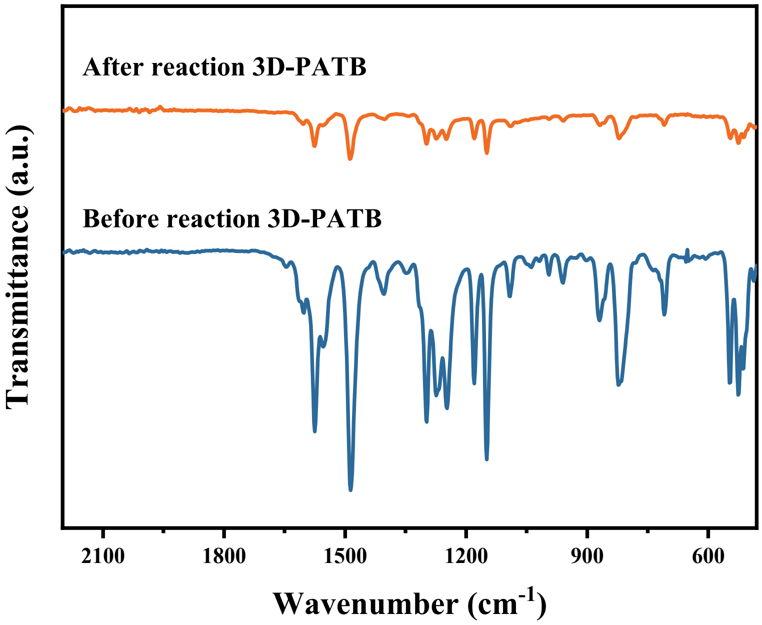

Figure S50. FT-IR spectra of 3D–PATB before and after reaction.

S10 General procedure for continuous flow set-up reactions

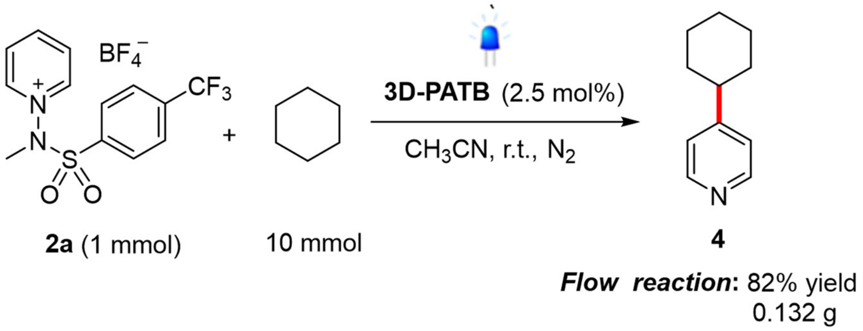

2a (404 mg, 1 mmol), cyclohexane (1.1 mL, 10 mmol), and 3D–PATB (16 mg, 25 µmol, 2.5 mol% catalyst loading based on the PATB linker) were mixed in anhydrous acetonitrile (30 mL) in a sealed continuous flow reactor. The resultant mixture was stirred under blue LED irradiation (440-450 nm) at room temperature in a N_2 atmosphere for 48 hours. The peristaltic pump flow rate is 40 mL/min. After that, the reactor was washed with MeCN (10 mL × 3), the solvent was removed under vacuum, and the residue was subjected to column chromatography on silica gel to give product **4** (0.132g, 82%).

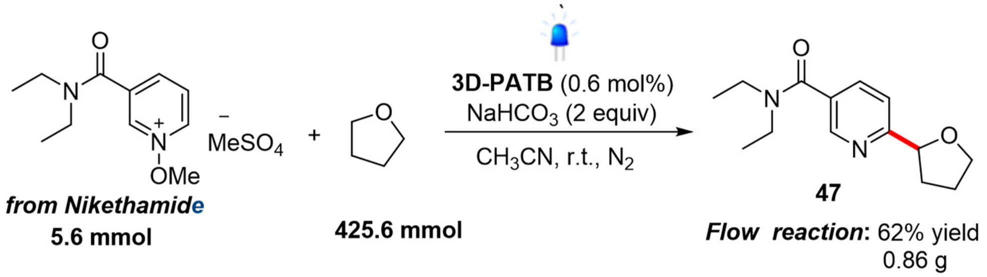

3-(diethylcarbamoyl)-1-methoxypyridin-1-ium (1.8 g, 5.6 mmol), tetrahydrofuran (34.72 mL, 425.6 mmol), $NaHCO_3$ (952 mg, 11.2 mmol) and 3D–PATB (20 mg, 31 μmol, 0.6 mol% catalyst loading based on the PATB linker) were mixed in anhydrous acetonitrile (30 mL) in a sealed continuous flow reactor. The resultant mixture was stirred under blue LED irradiation (440-450 nm) at room temperature in a N_2 atmosphere for 30 hours. The peristaltic pump flow rate was 40 mL/min. After that, the reactor was washed with MeCN (10 mL × 3), the solvent was removed under vacuum, and the residue was subjected to column chromatography on silica gel to give product **47** (0.86 g, 62%).

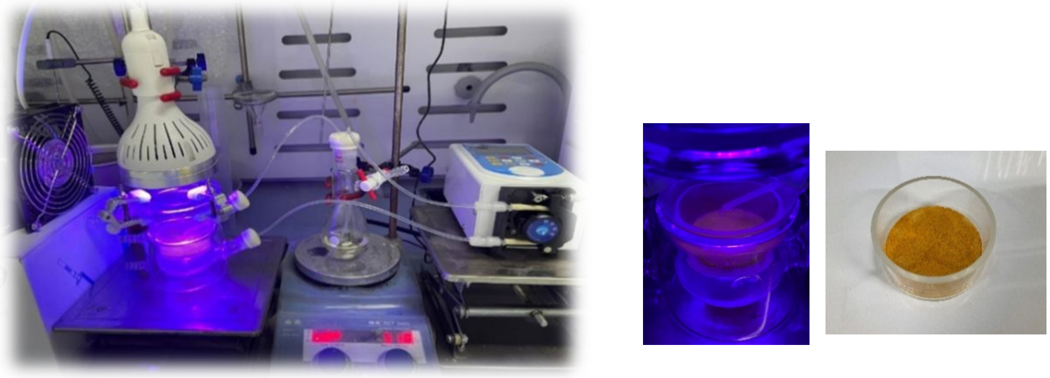

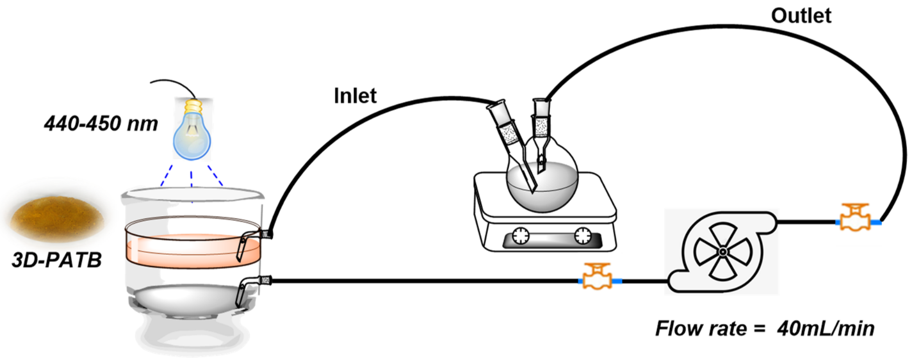

Figure S51. Continuous flow set-up reaction.

S11 NMR spectra

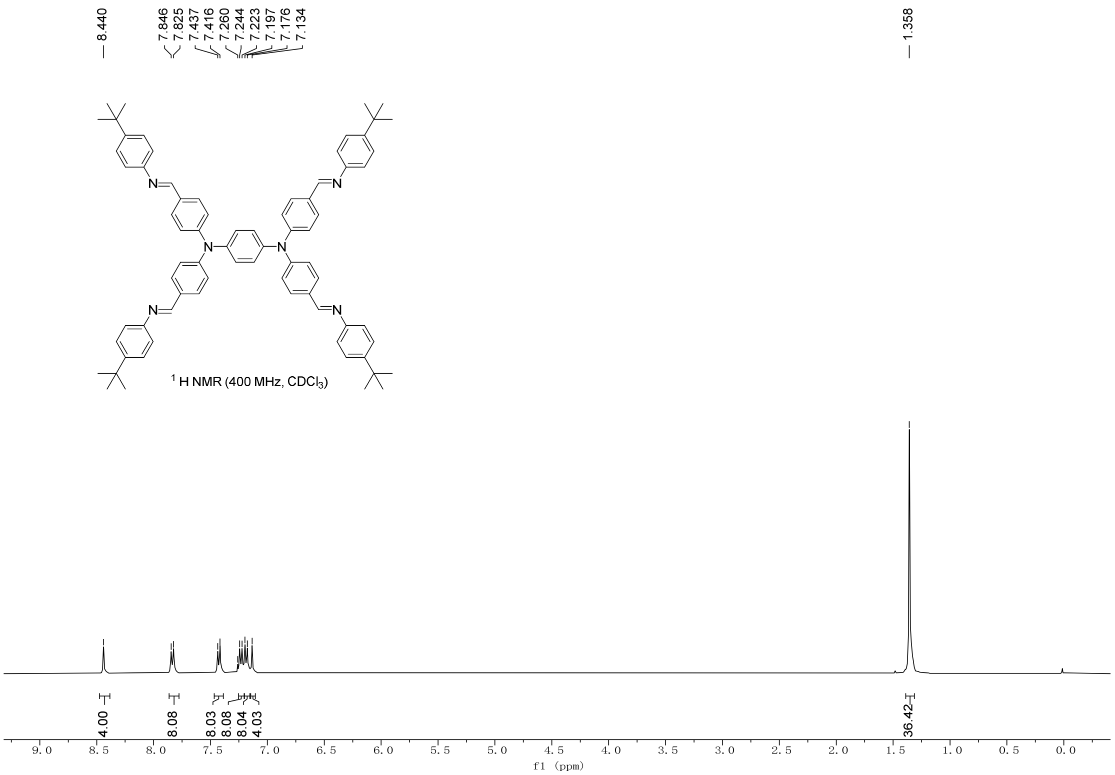

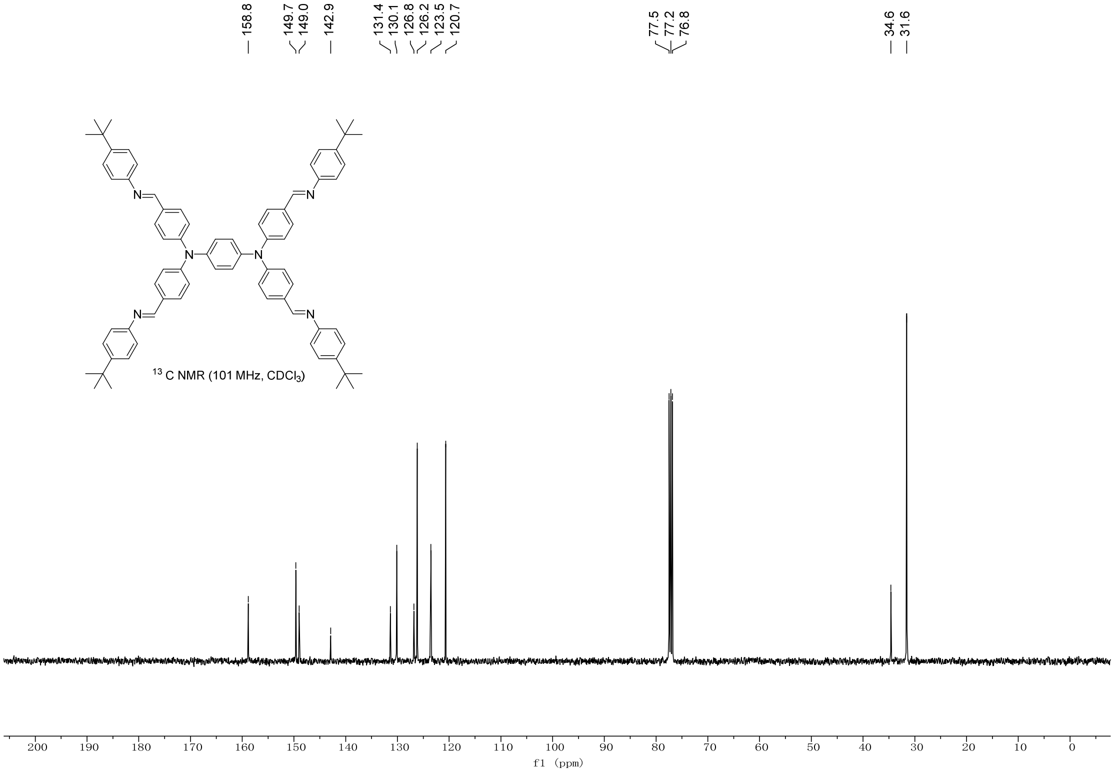

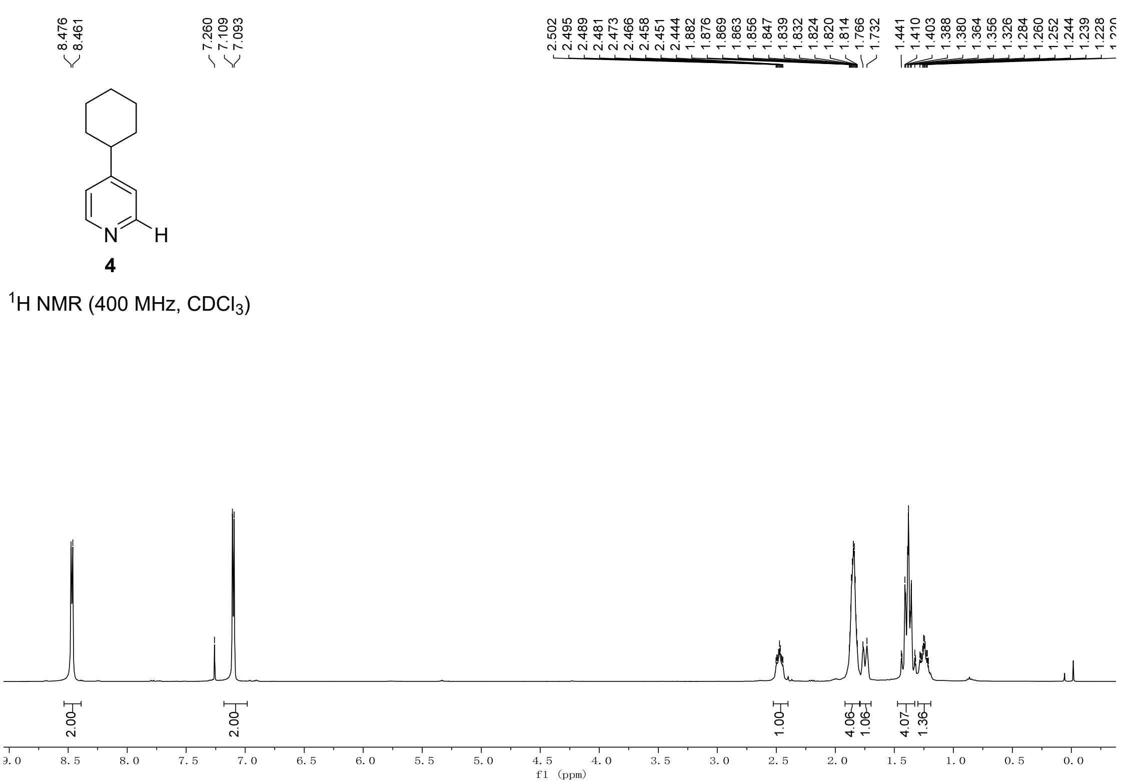

4

^{1}H NMR (400 MHz, $CDCl_3$)

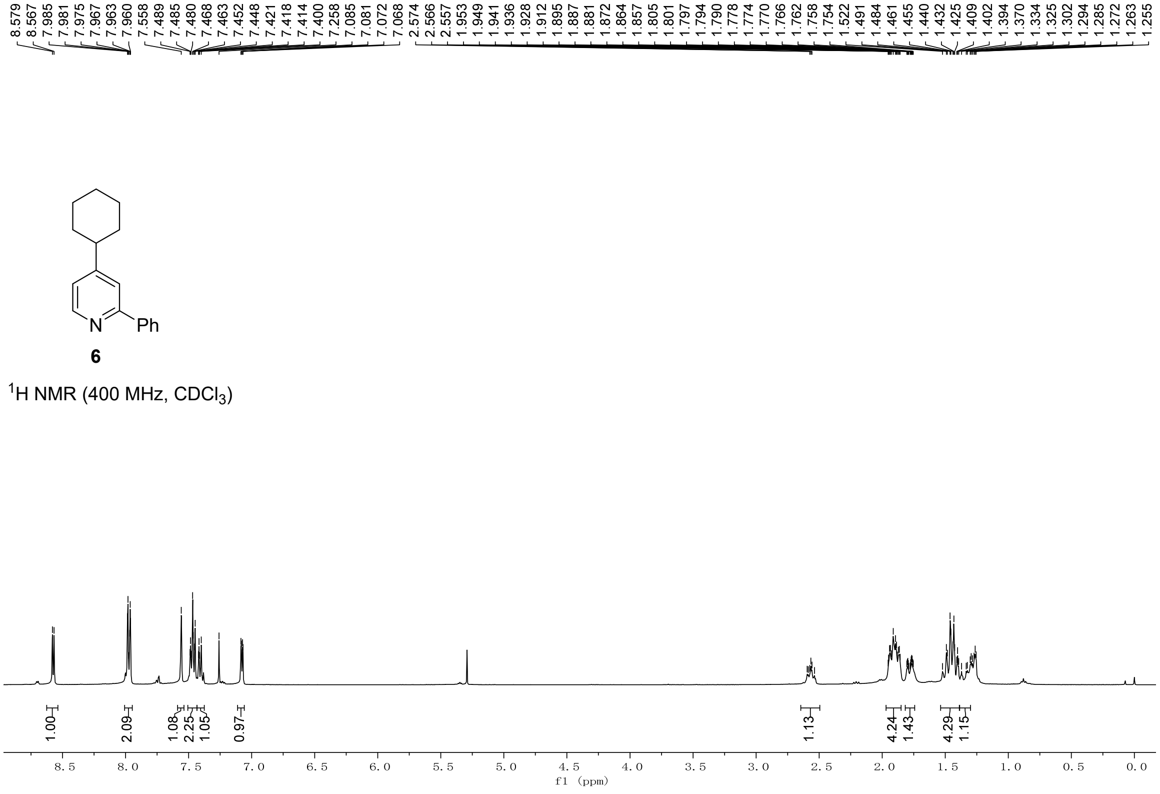

6

^{1}H NMR (400 MHz, $CDCl_3$)

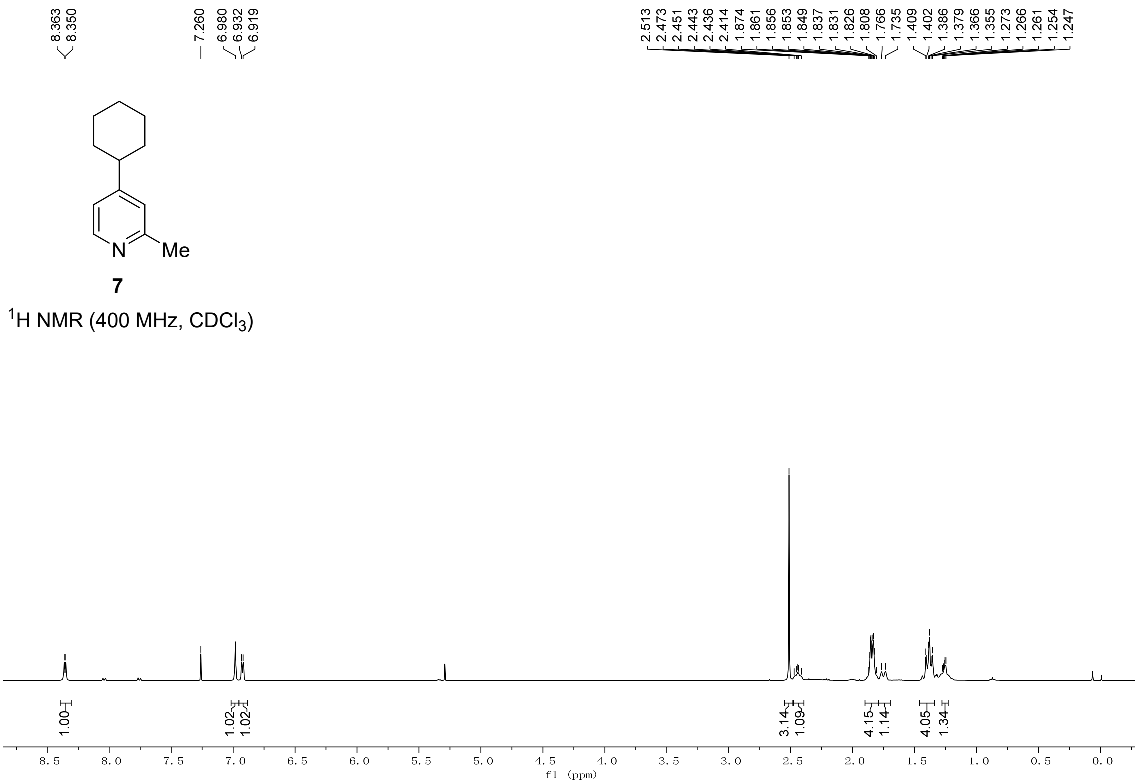

7

^{1}H NMR (400 MHz, $CDCl_3$)

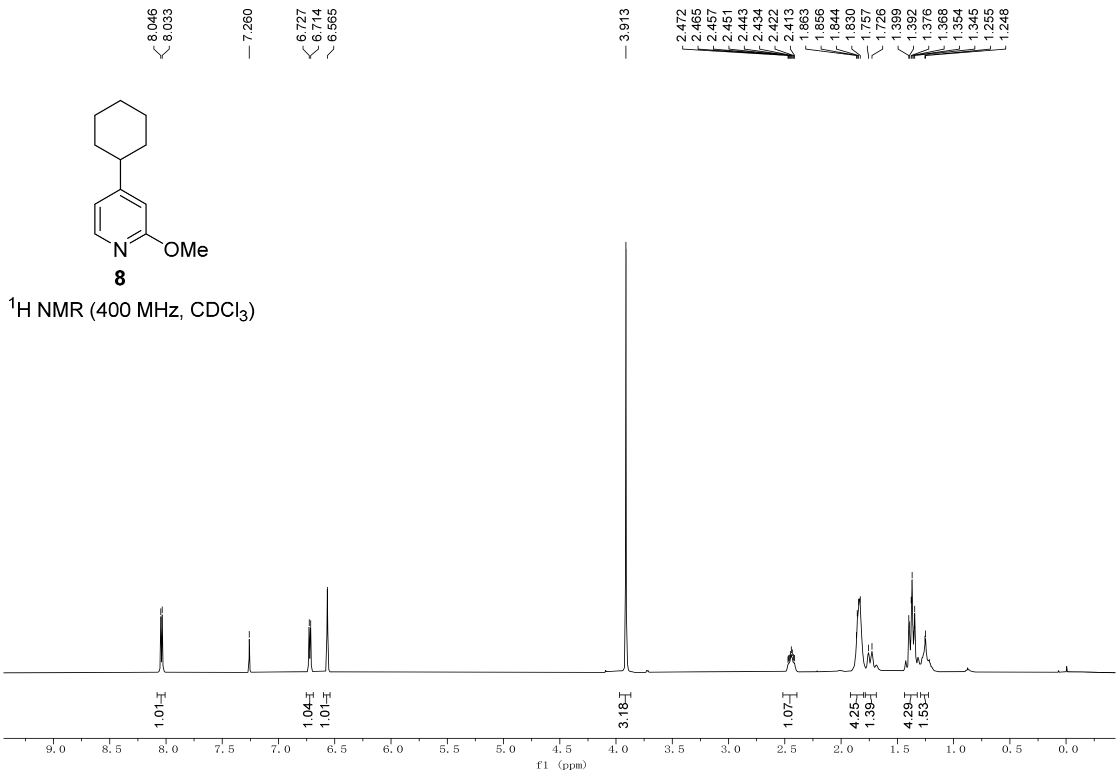

8

^{1}H NMR (400 MHz, $CDCl_3$)

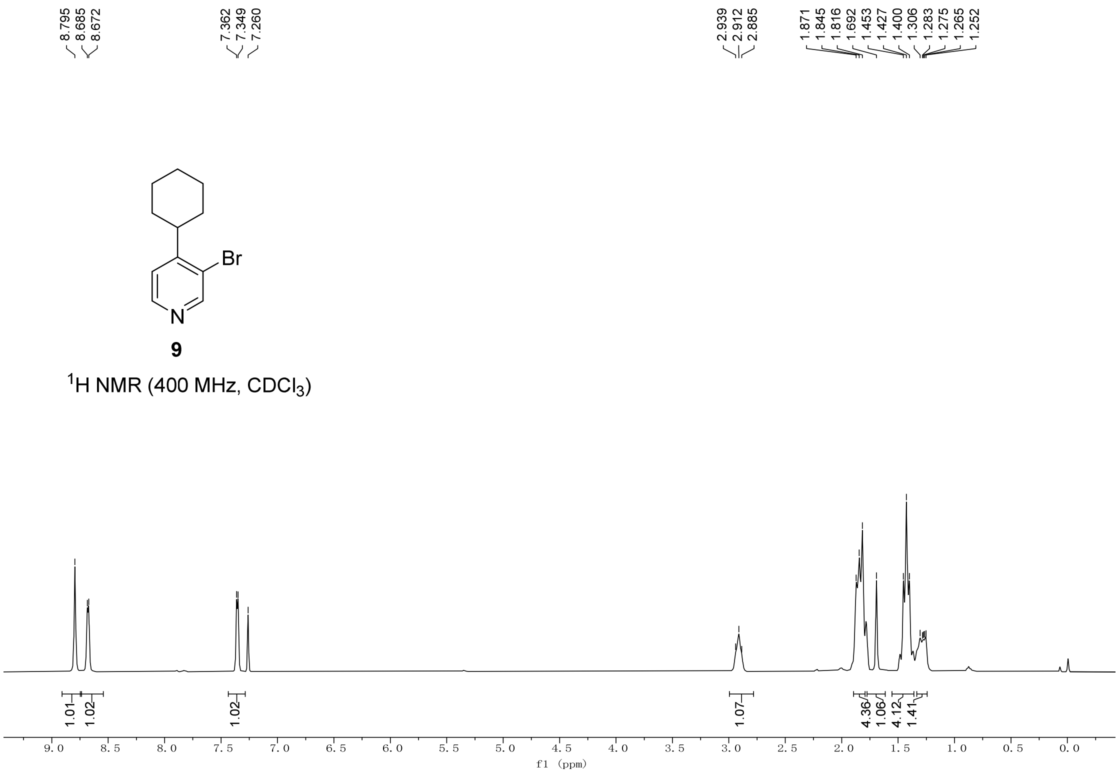

9

^{1}H NMR (400 MHz, $CDCl_3$)

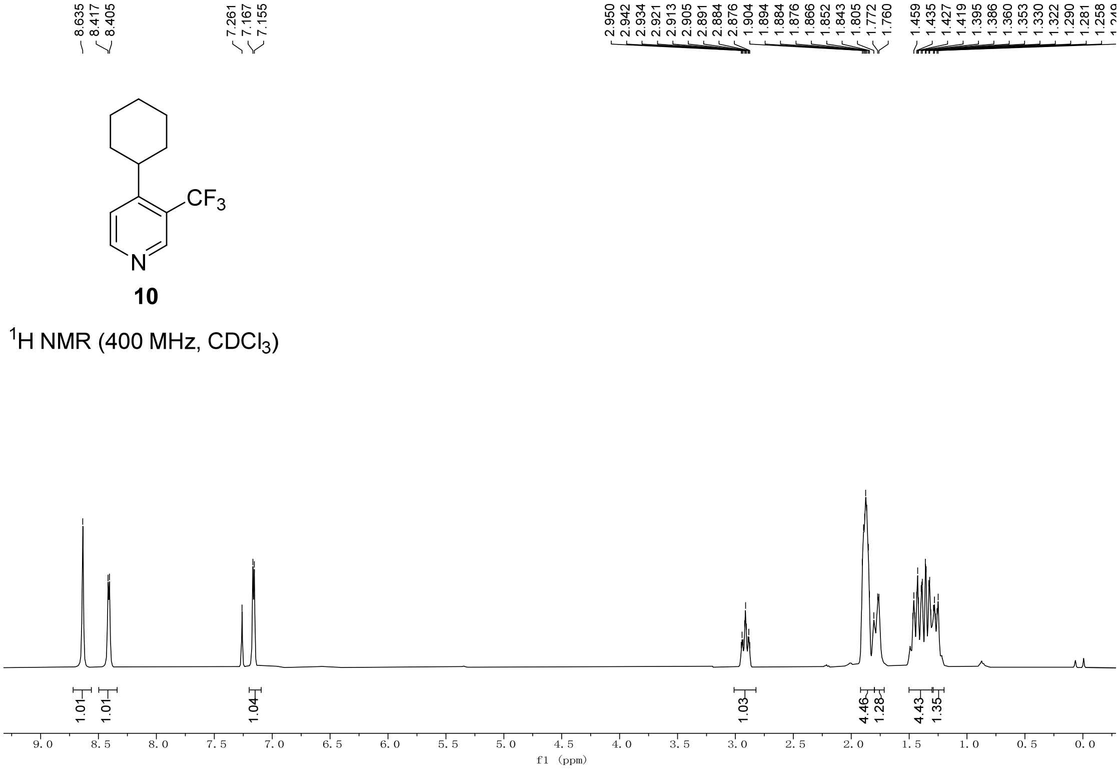

10

^{1}H NMR (400 MHz, $CDCl_3$)

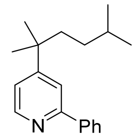

11

^1H NMR (400 MHz, $CDCl_3$)

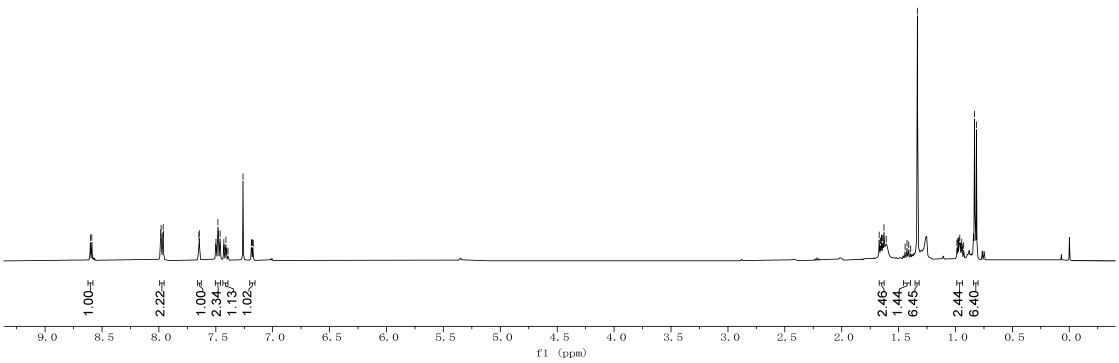

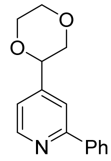

12

^1H NMR (400 MHz, $CDCl_3$)

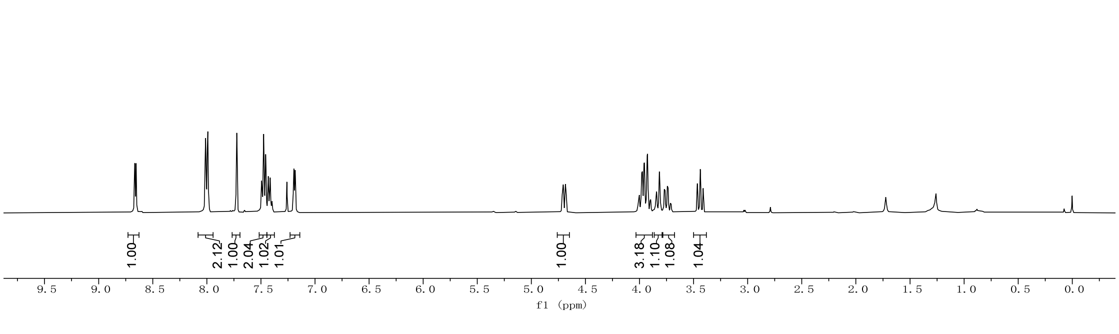

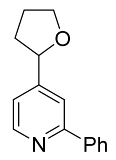

13

^{1}H NMR (400 MHz, $CDCl_3$)

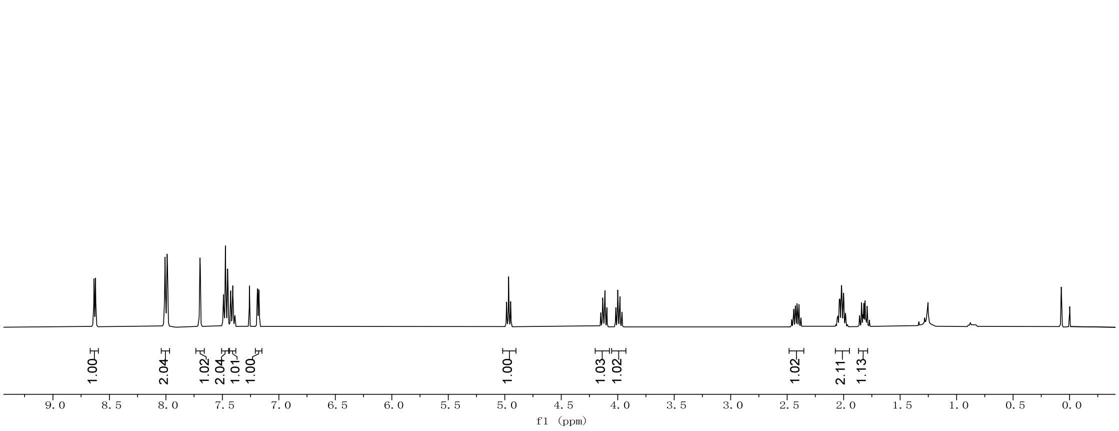

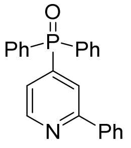

14

^{1}H NMR (400 MHz, $CDCl_3$)

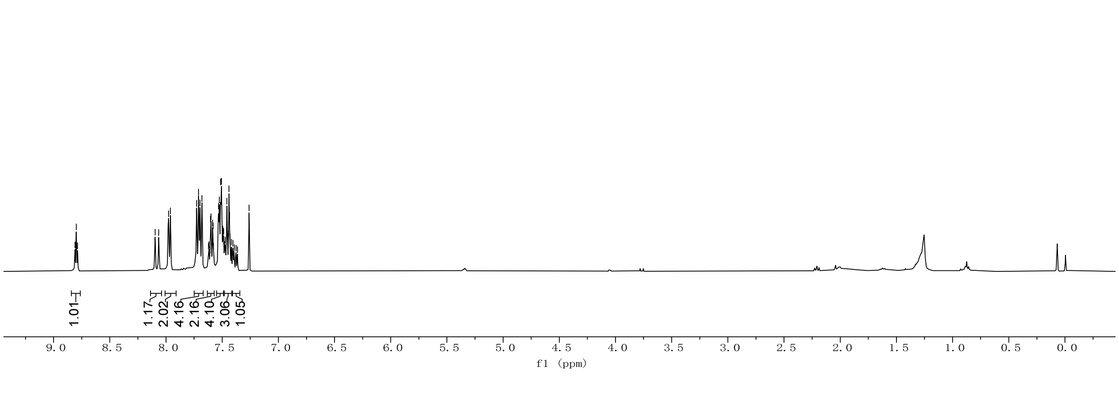

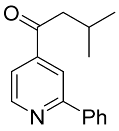

15

^{1}H NMR (400 MHz, $CDCl_3$)

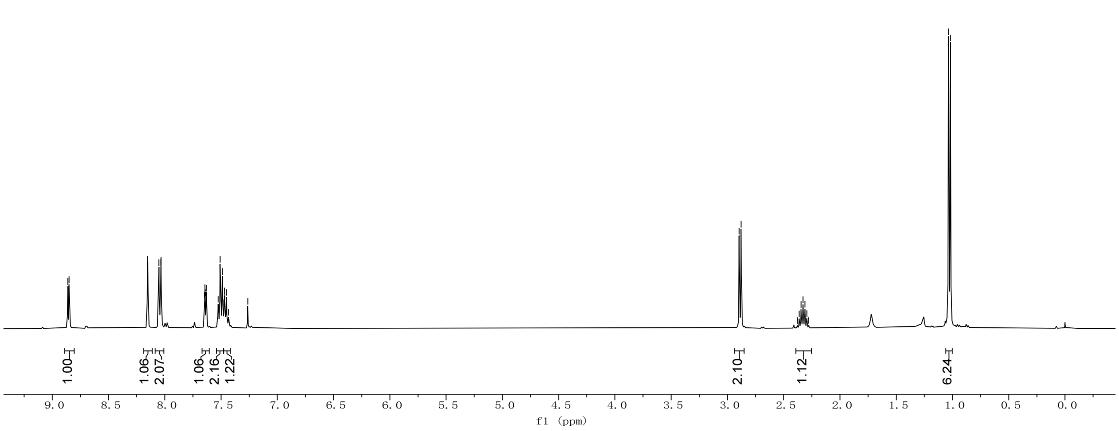

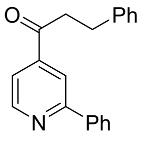

16

^{1}H NMR (400 MHz, $CDCl_3$)

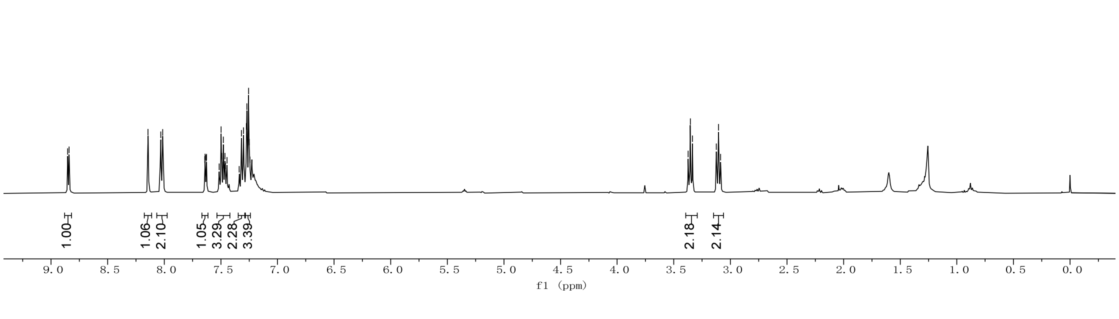

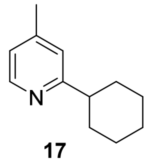

17

^{1}H NMR (400 MHz, $CDCl_3$)

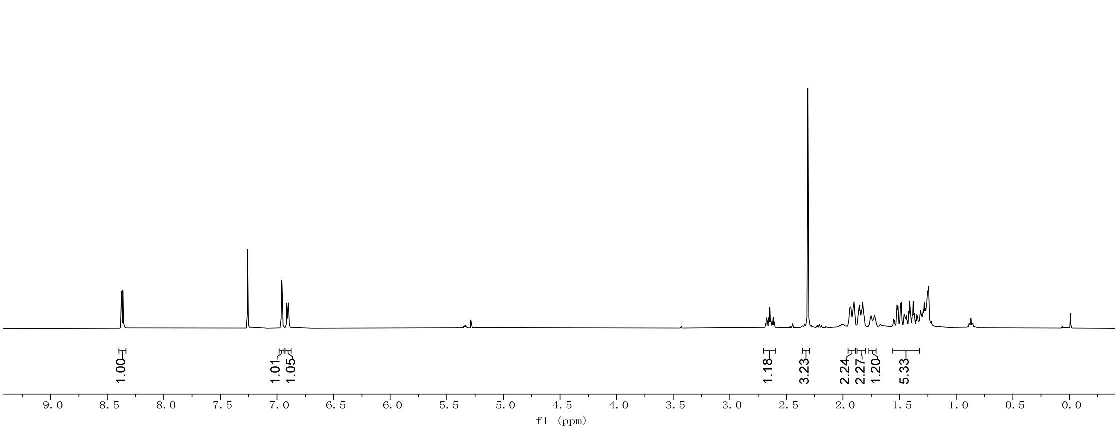

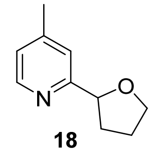

18

^{1}H NMR (400 MHz, $CDCl_3$)

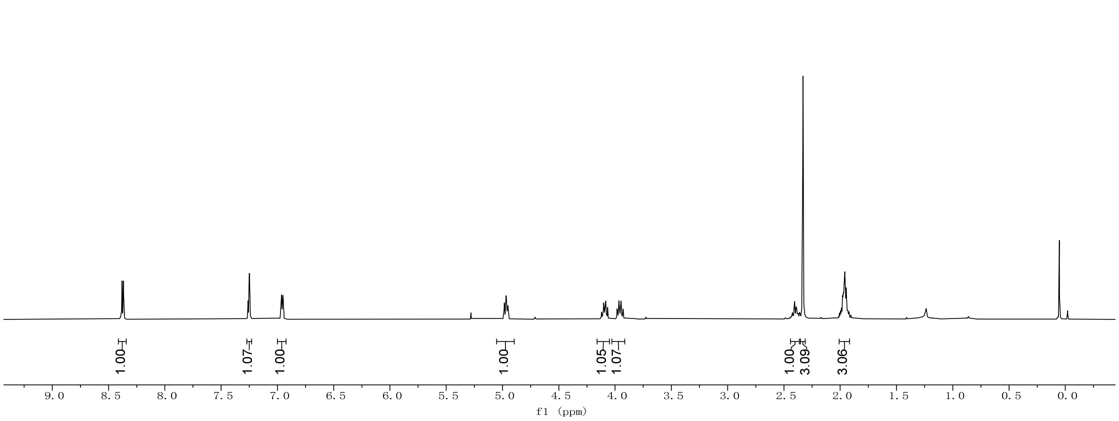

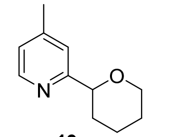

19

^{1}H NMR (400 MHz, $CDCl_3$)

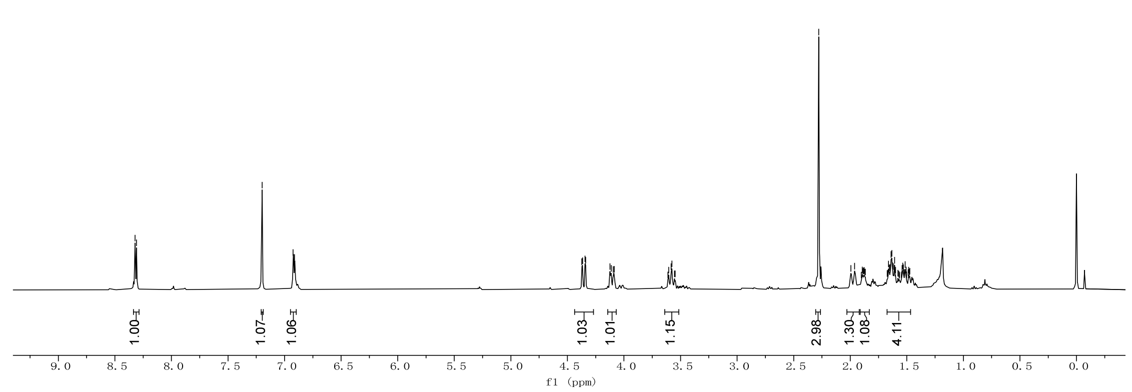

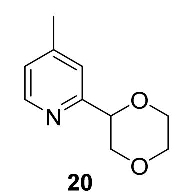

20

^{1}H NMR (400 MHz, $CDCl_3$)

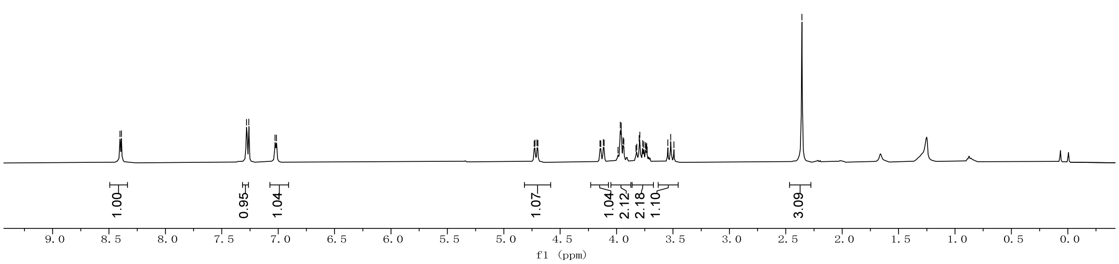

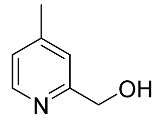

21

^{1}H NMR (400 MHz, $CDCl_3$)

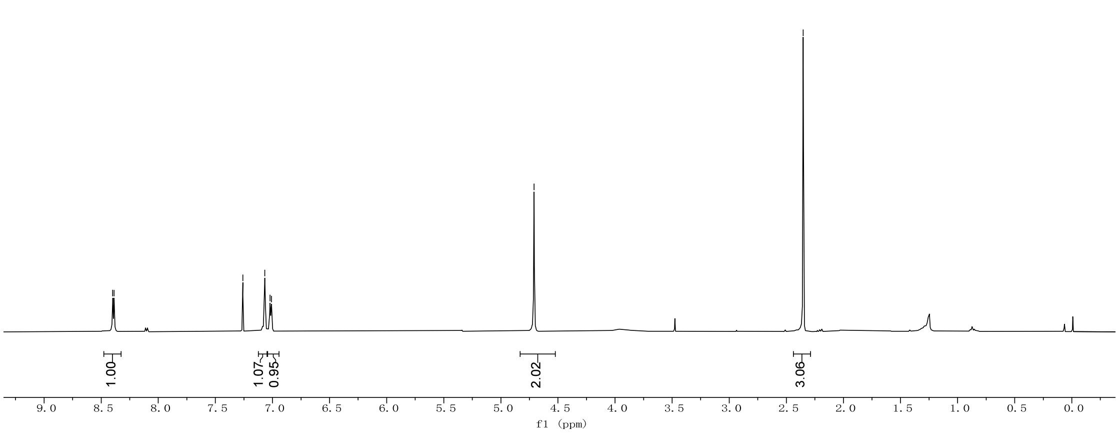

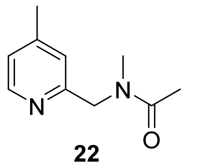

22

^{1}H NMR (400 MHz, $CDCl_3$)

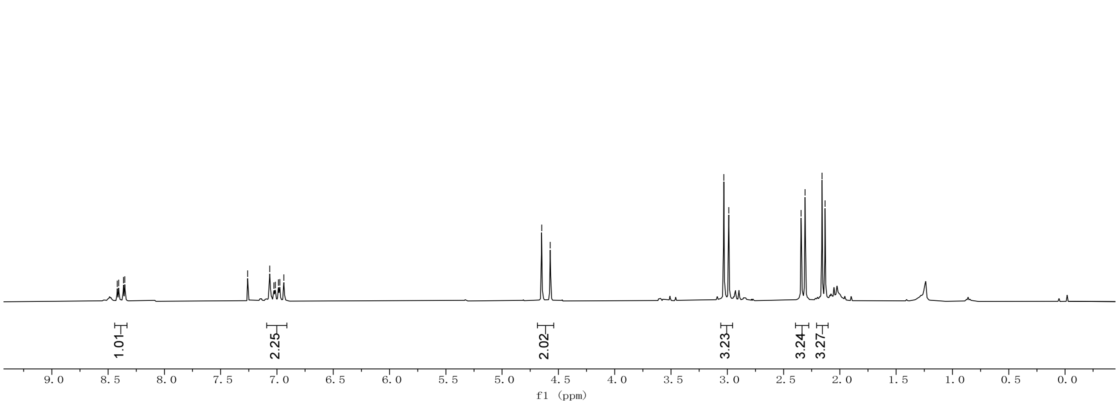

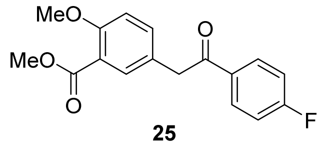

25

^{1}H NMR (400 MHz, $CDCl_3$)

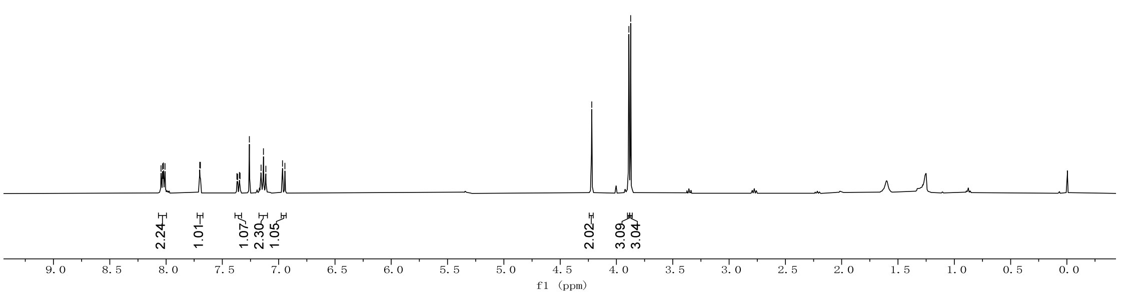

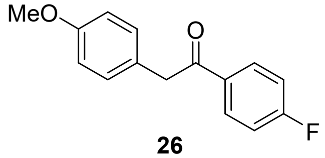

26

^{1}H NMR (400 MHz, $CDCl_3$)

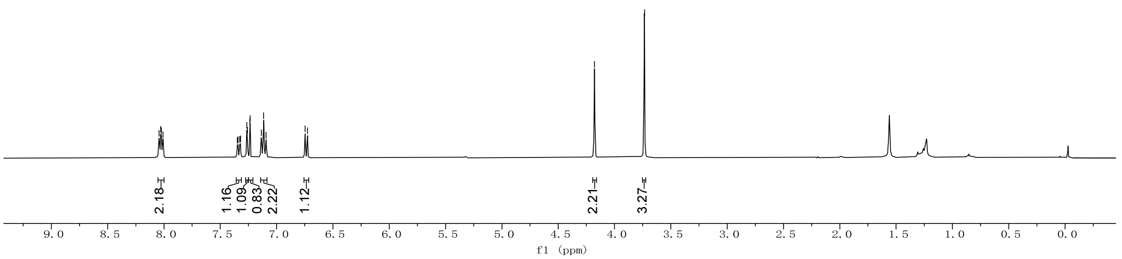

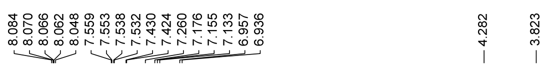

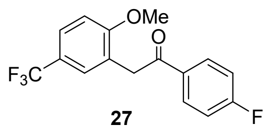

27

^{1}H NMR (400 MHz, CDCl$_3$)

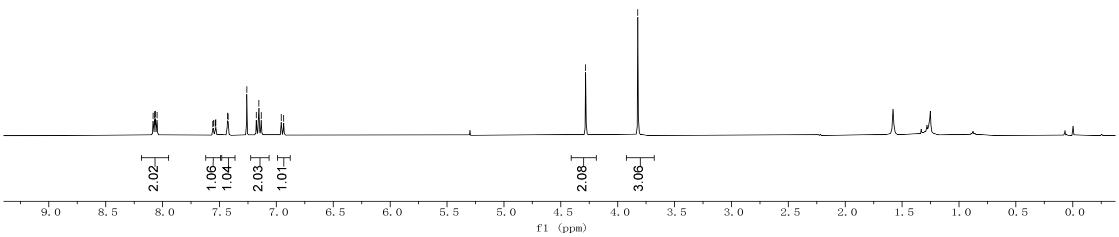

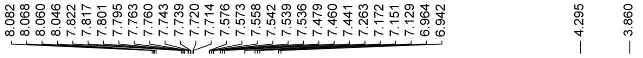

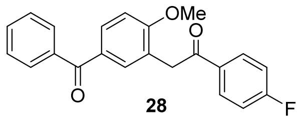

28

^{1}H NMR (400 MHz, CDCl$_3$)

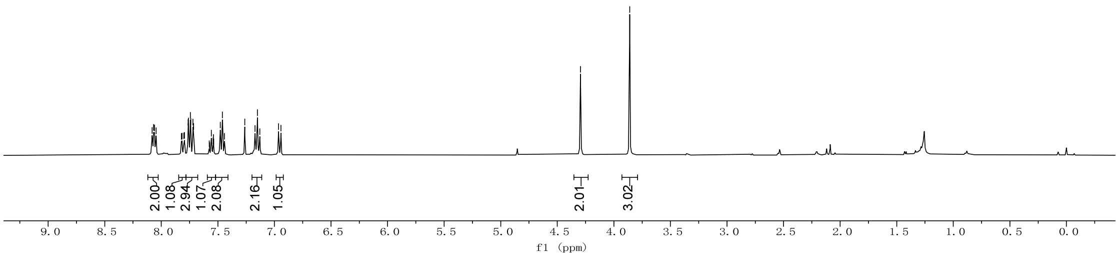

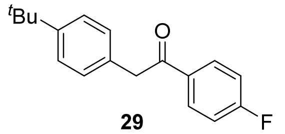

29

^{1}H NMR (400 MHz, $CDCl_3$)

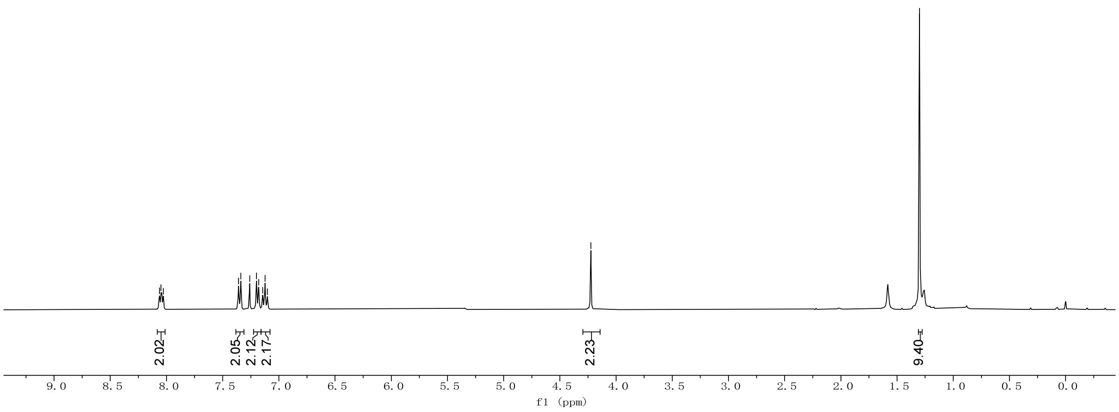

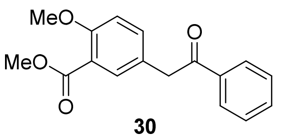

30

^{1}H NMR (400 MHz, $CDCl_3$)

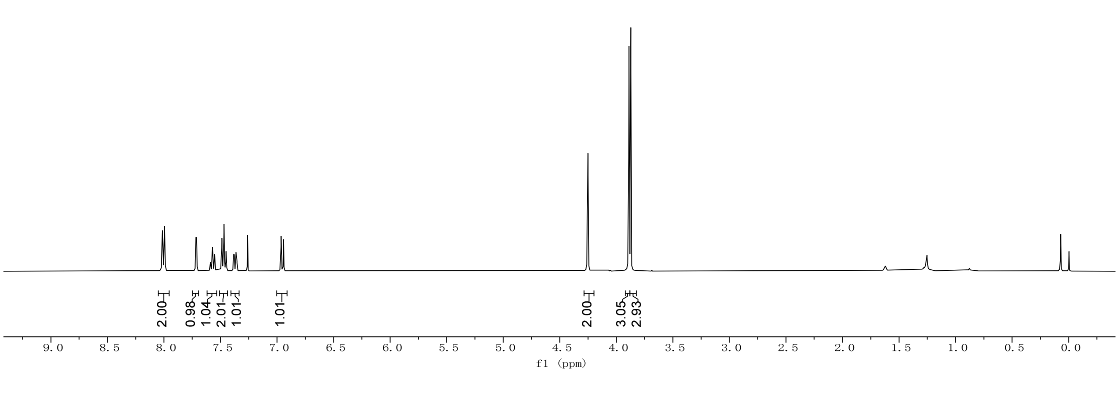

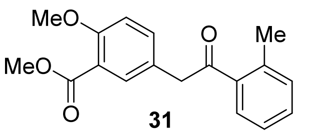

31

^{1}H NMR (400 MHz, $CDCl_3$)

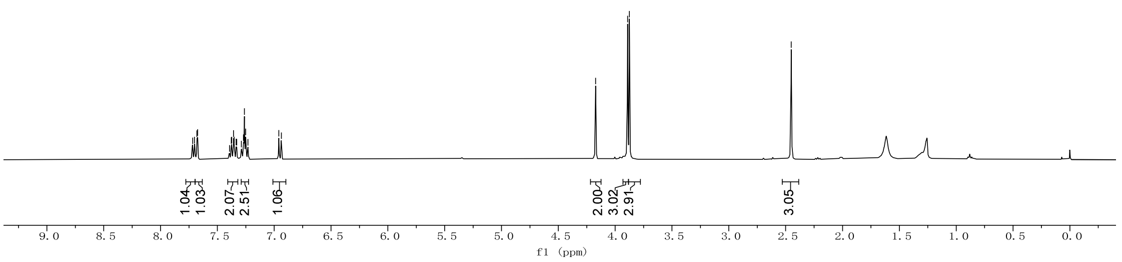

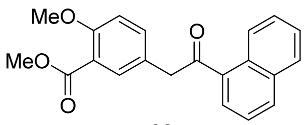

32

^{1}H NMR (400 MHz, $CDCl_3$)

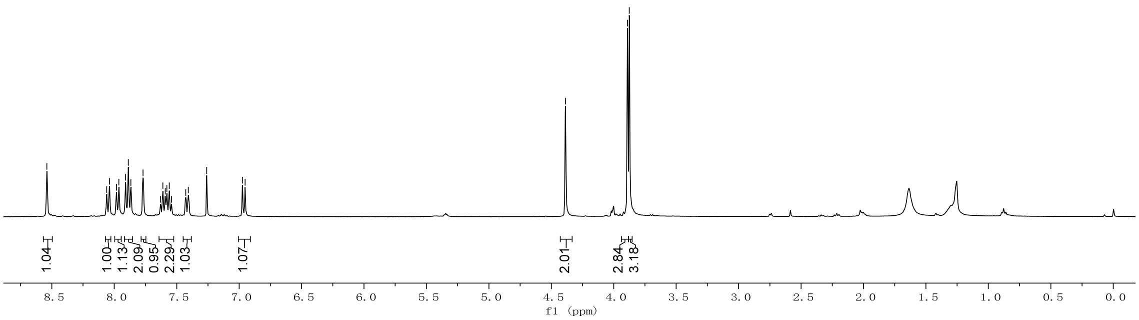

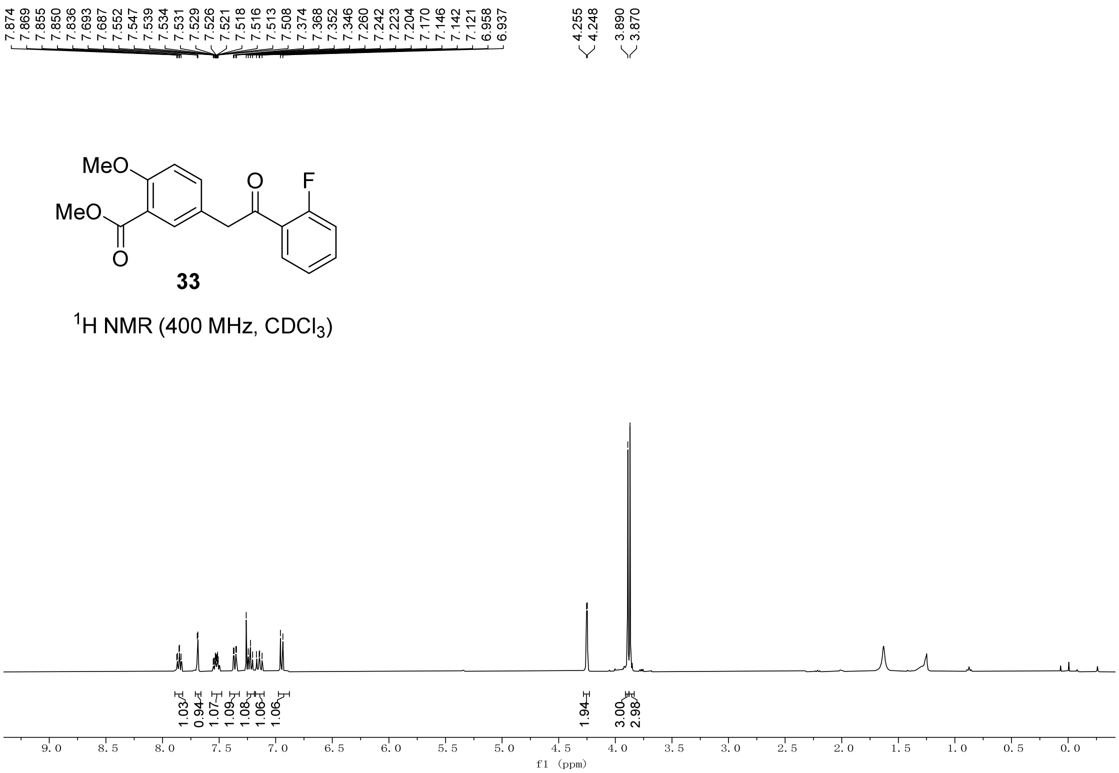

33

^{1}H NMR (400 MHz, CDCl$_3$)

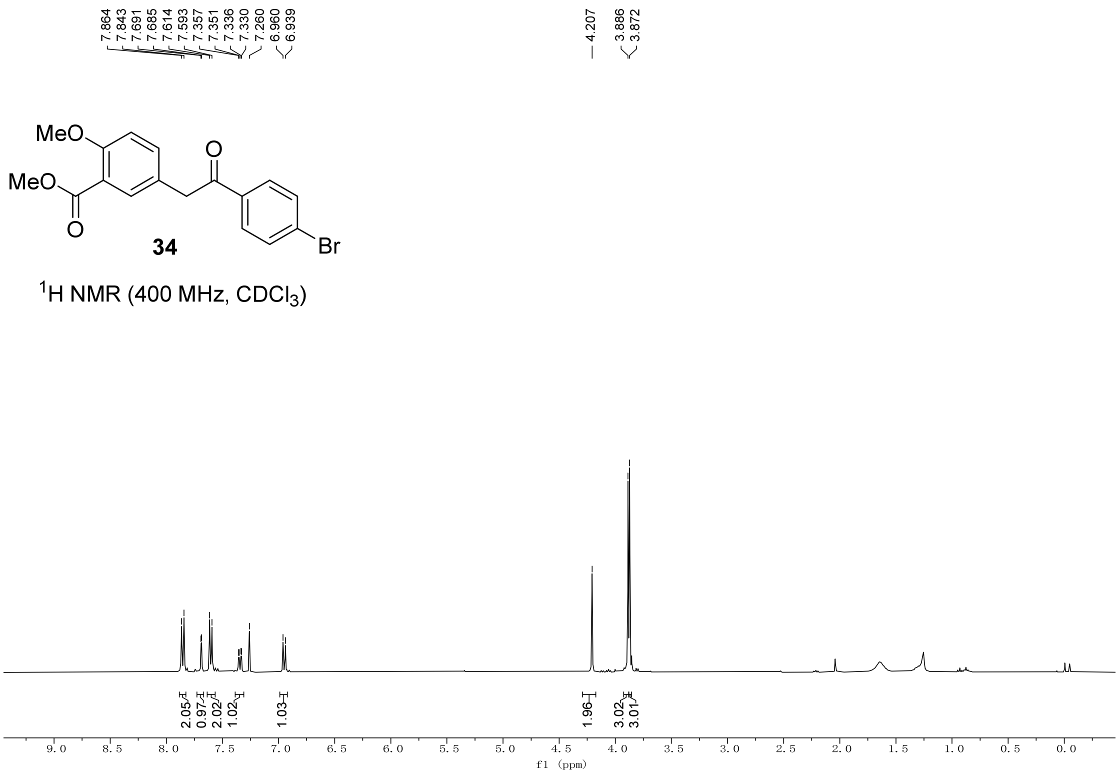

34

^{1}H NMR (400 MHz, CDCl$_3$)

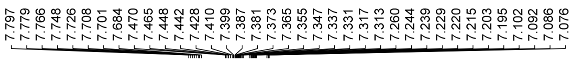

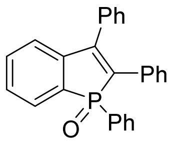

37

^{1}H NMR (400 MHz, $CDCl_3$)

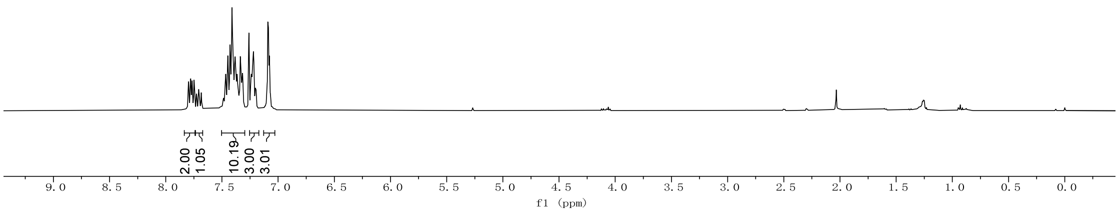

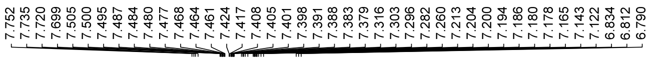

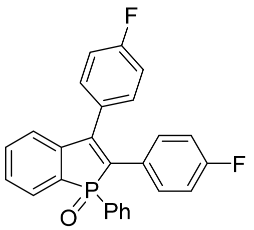

38

^{1}H NMR (400 MHz, $CDCl_3$)

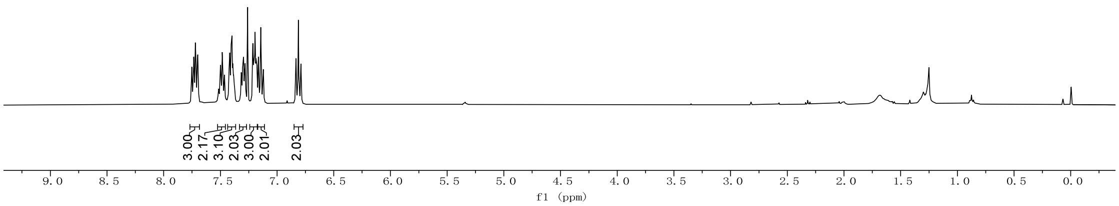

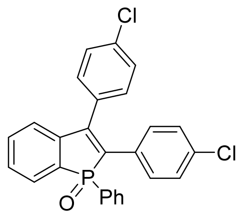

39

^{1}H NMR (400 MHz, $CDCl_3$)

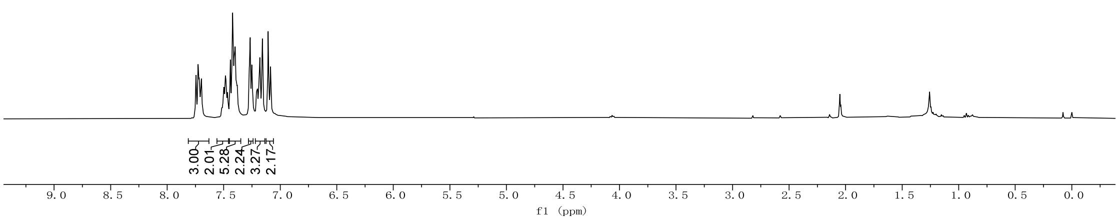

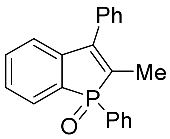

40

^{1}H NMR (400 MHz, $CDCl_3$)

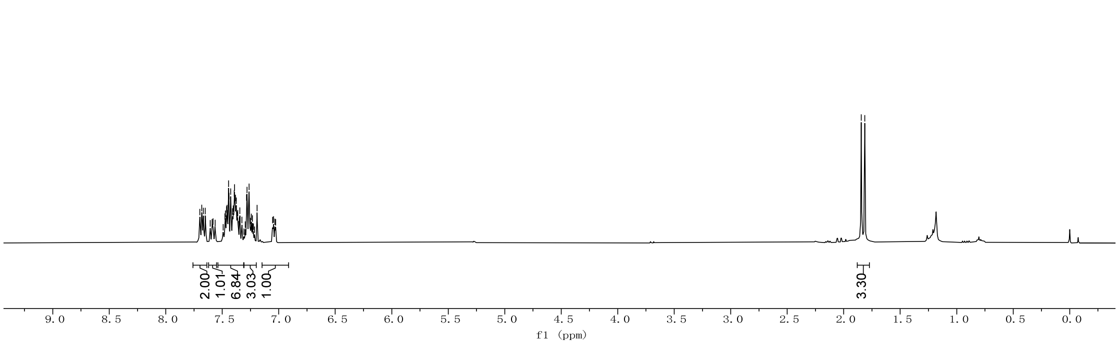

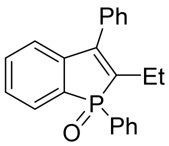

41

^1H NMR (400 MHz, $CDCl_3$)

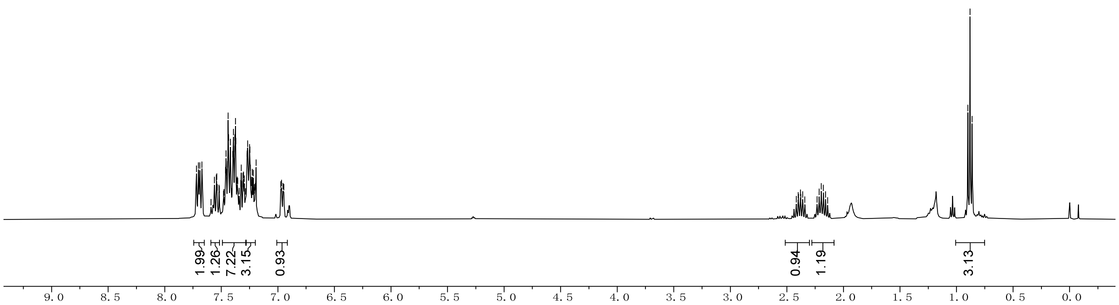

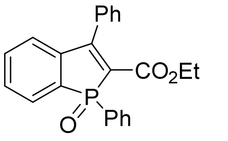

42

^1H NMR (400 MHz, $CDCl_3$)

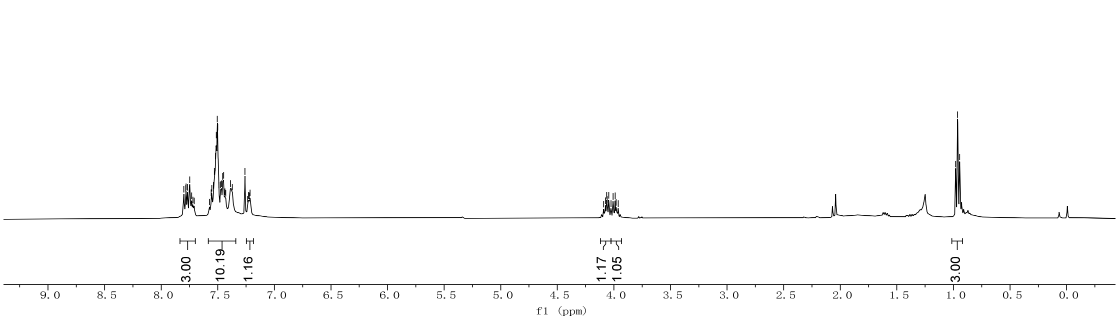

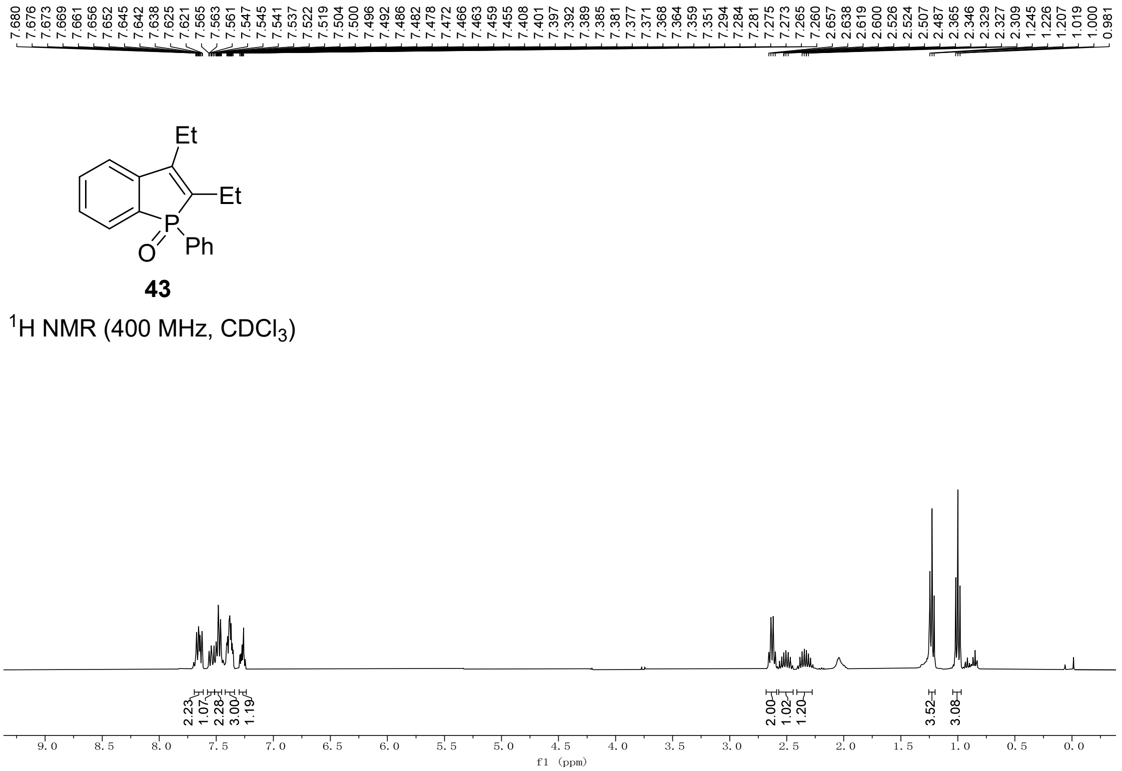

43

^{1}H NMR (400 MHz, $CDCl_3$)

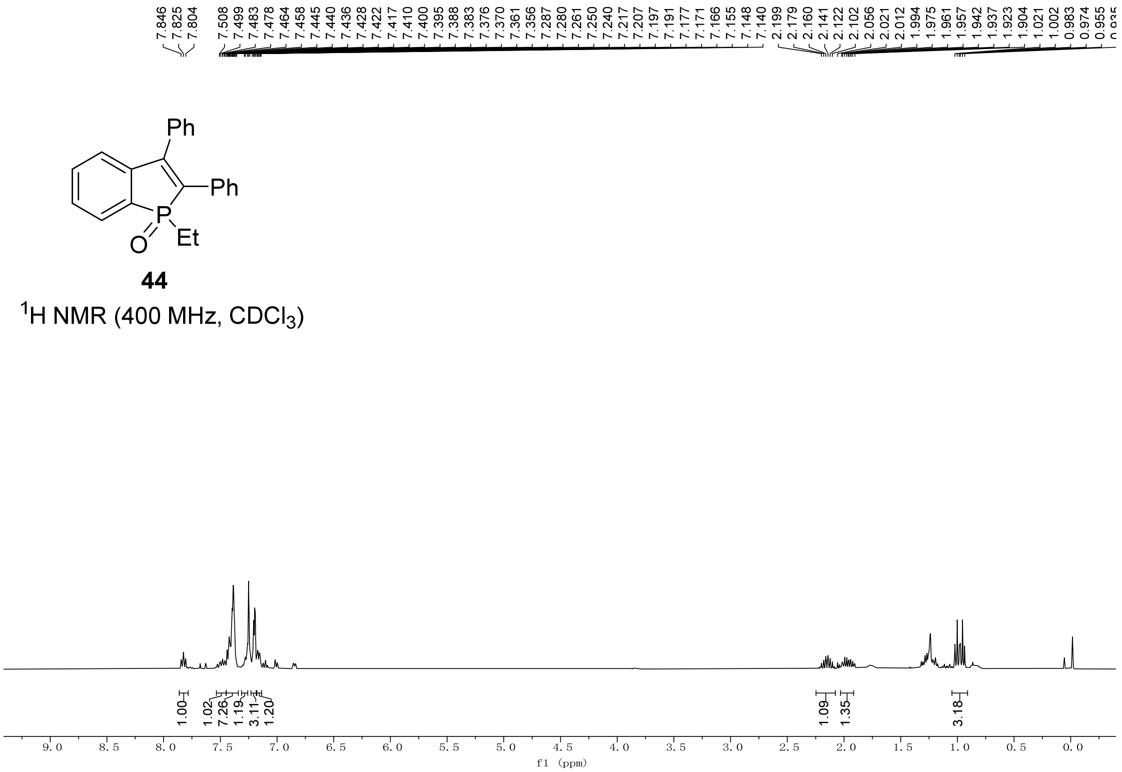

44

^{1}H NMR (400 MHz, $CDCl_3$)

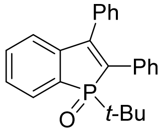

45

^{1}H NMR (400 MHz, $CDCl_3$)

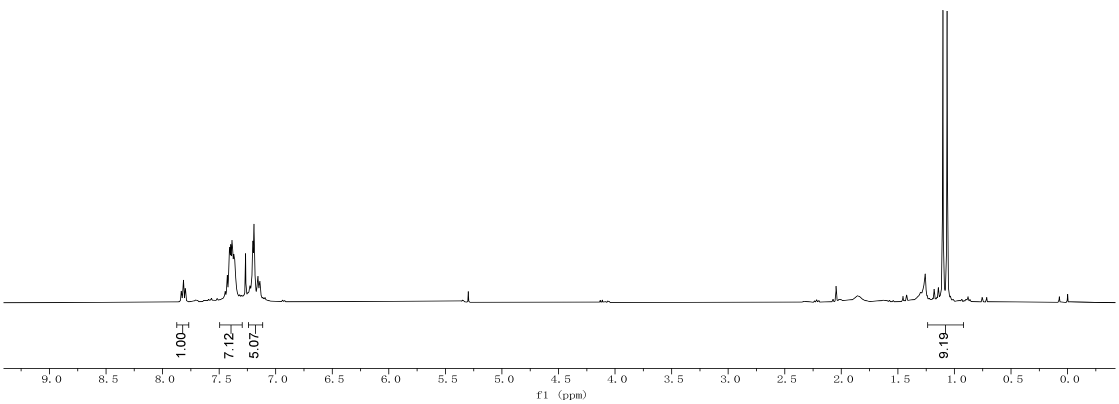

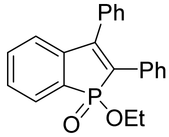

46

^{1}H NMR (400 MHz, $CDCl_3$)

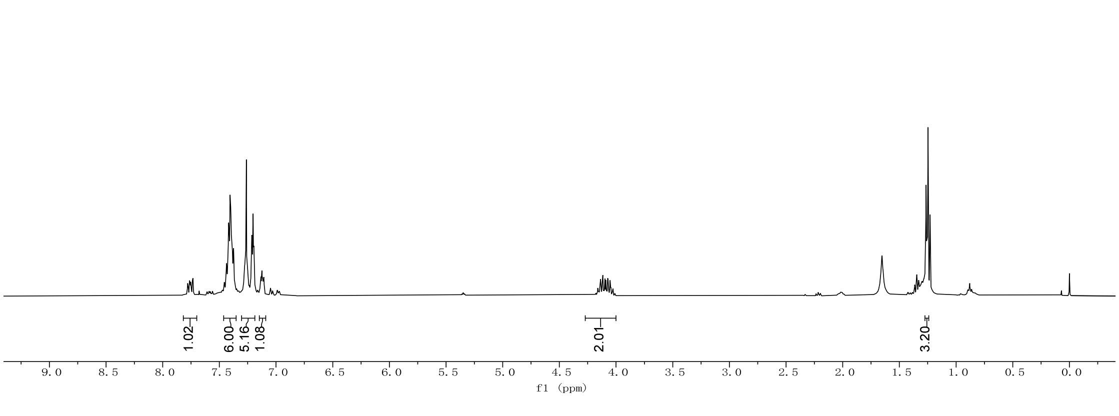

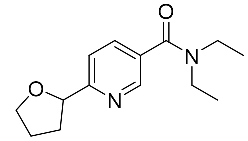

47

^1H NMR (400 MHz, $CDCl_3$)

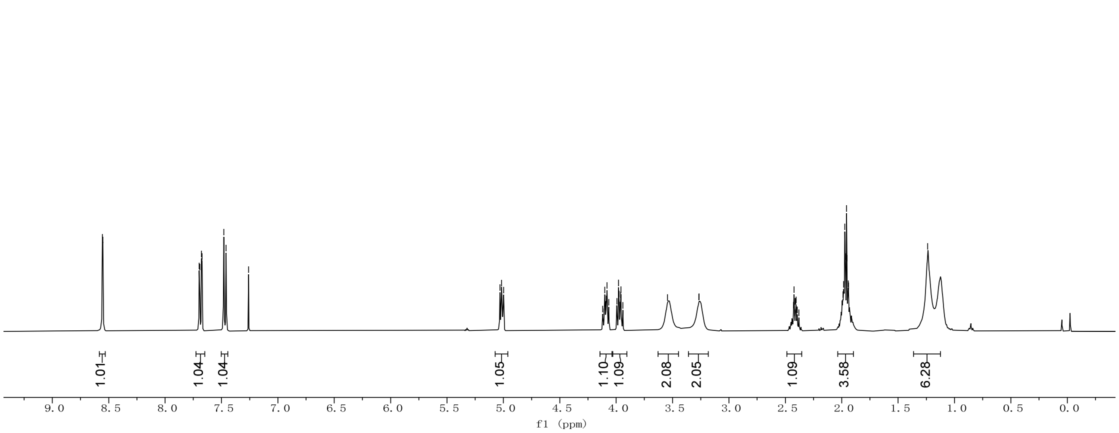

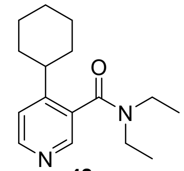

48

^1H NMR (400 MHz, $CDCl_3$)

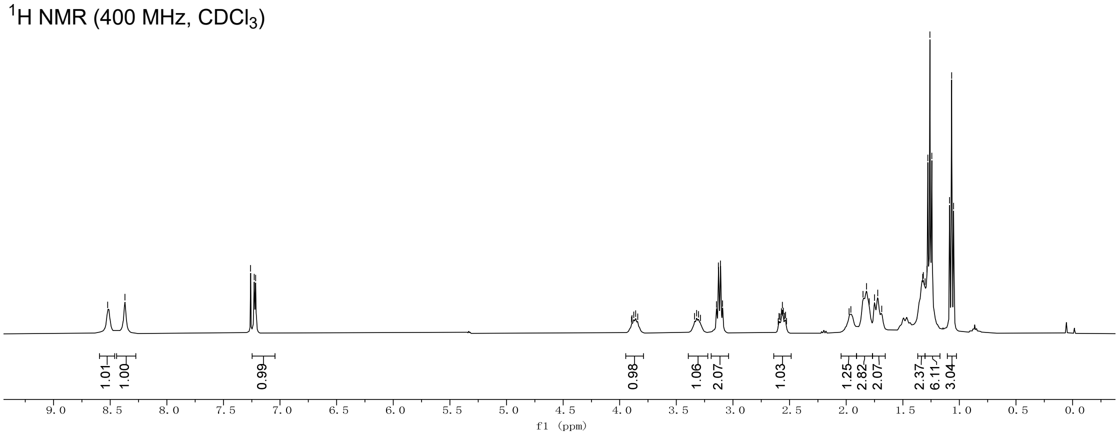

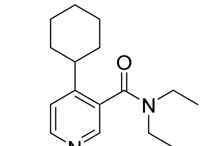

48

^{13}C NMR (101 MHz, $CDCl_3$)

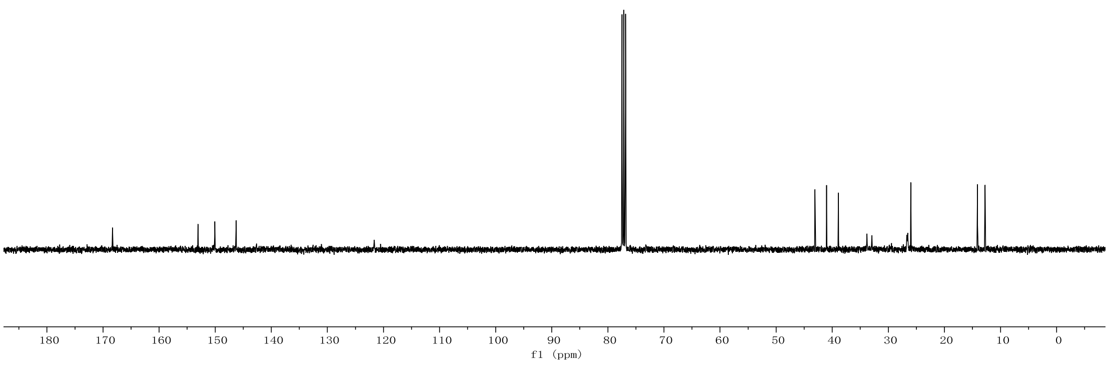

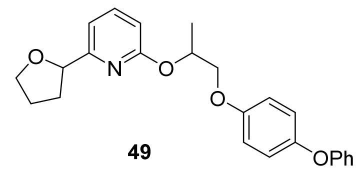

49

^{1}H NMR (400 MHz, $CDCl_3$)

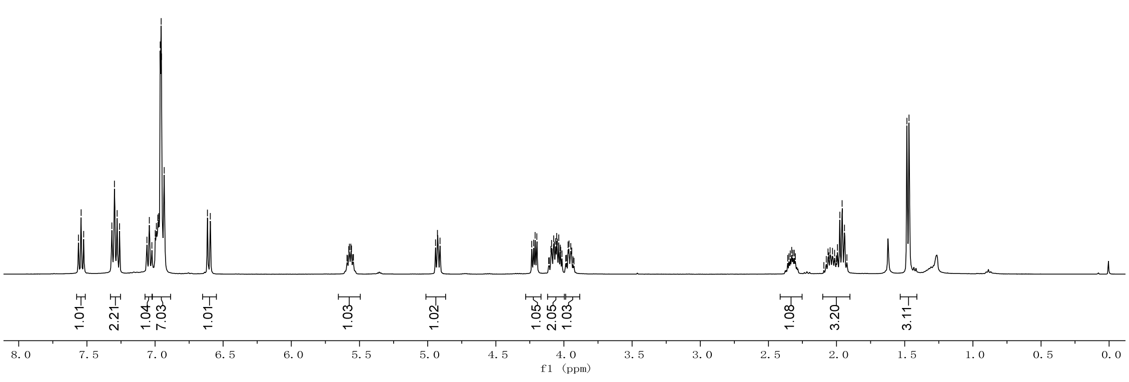

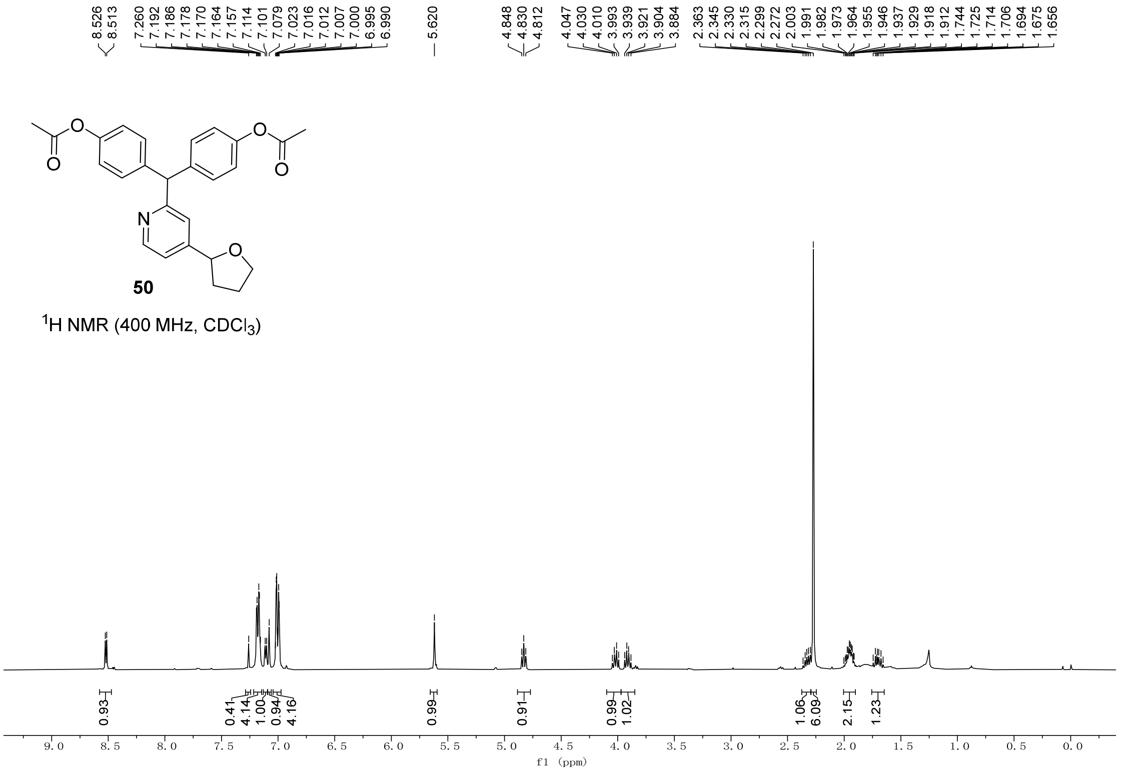

50

^{1}H NMR (400 MHz, $CDCl_3$)

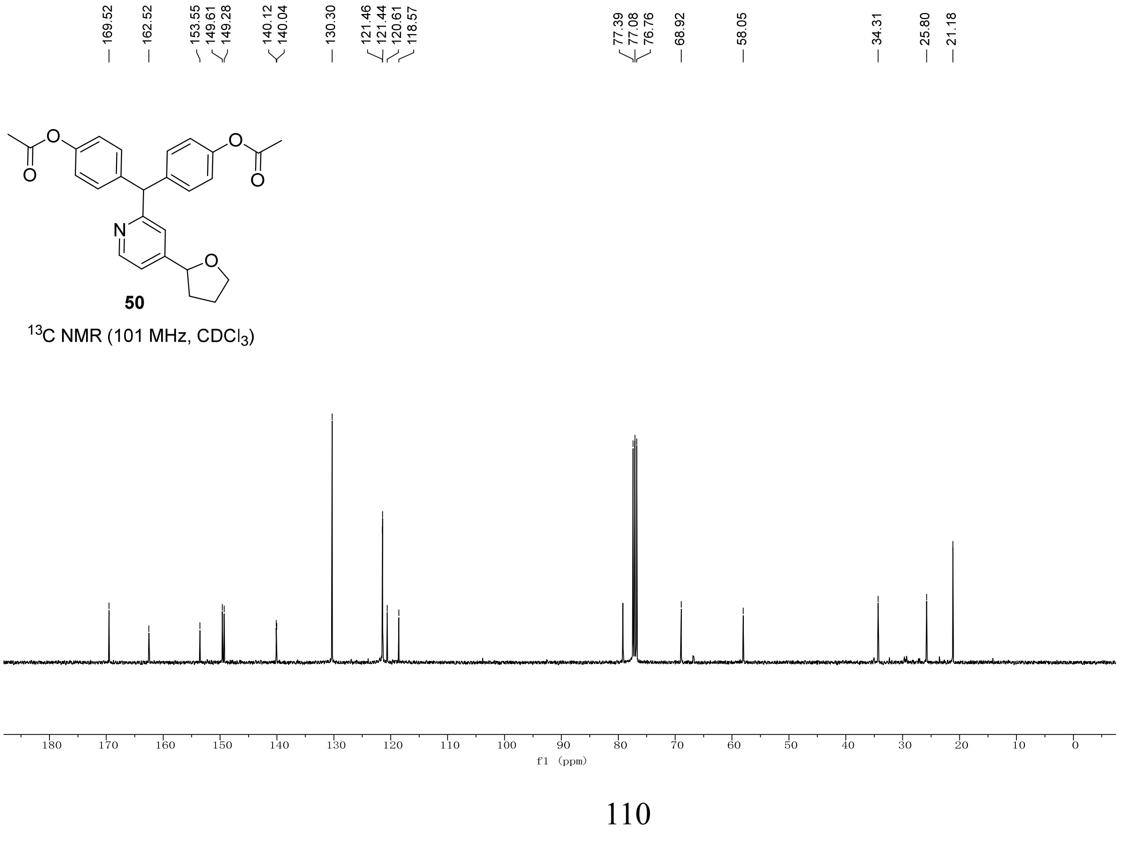

50

^{13}C NMR (101 MHz, $CDCl_3$)

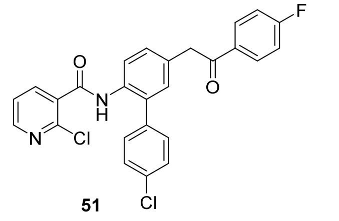

51

^{1}H NMR (400 MHz, $CDCl_3$)

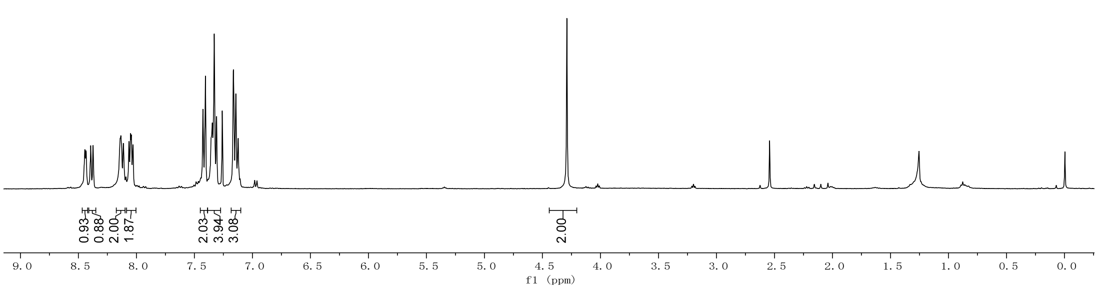

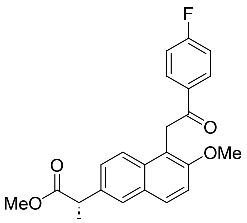

52

^{1}H NMR (400 MHz, $CDCl_3$)

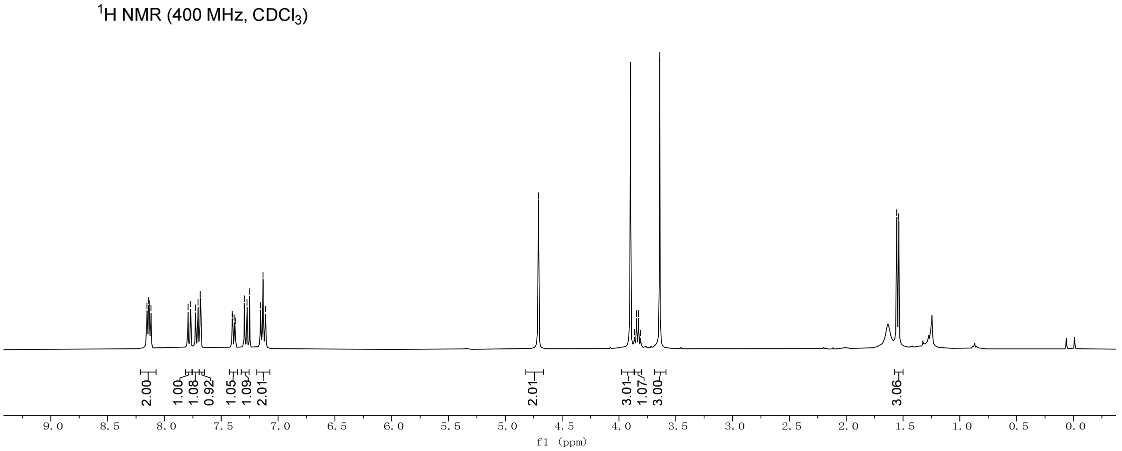

S12 References

[1]. Lee W, Jung S, Kim M, Hong S, Site-selective direct C–H pyridylation of unactivated alkanes by triplet excited anthraquinone. *J Am Chem Soc.* 2021; **143**: 3003-3012.

[2]. Lin J, Ouyang J, Liu T *et al*. Metal-organic framework boosts heterogeneous electron donor–acceptor catalysis. *Nat Commun.* 2023; **14**: 7757-7767.

[3]. Dewanji A, vanDalsen L, Rossi-Ashton JA *et al*. A general arene C–H functionalization strategy via electron donor–acceptor complex photoactivation. *Nat Chem.* 2023; **15**: 43-52.

[4]. Gui B, Xin J, Cheng Y *et al*. Crystallization of dimensional isomers in covalent organic frameworks. *J Am Chem Soc.* 2023; **145**: 11276-11281.

[5]. Meuwsen RGA. 1-Aminopyridinium iodide. *Org. Synth.* 1963; **43**: 1-5.

[6]. Le KKA, Nguyen H, Daugulis O. 1-Aminopyridinium ylides as monodentate directing groups for sp(3) C-H bond functionalization. *J Am Chem Soc.* 2019; **141**: 14728-14735.

[7]. Quint V, Morlet-Savary F, Lohier J-F *et al.* Metal-free, visible light-photocatalyzed synthesis of benzo[b]phosphole oxides: synthetic and mechanistic investigations. *J Am Chem Soc.* 2016; **138**: 7436-7441.

[8]. Cismesia M, Yoon T. Characterizing chain processes in visible light photoredox catalysis. Chem. Sci. 2015; **6**: 5426-5434.

[9]. Kresse G, Furthmüller J. Efficient iterative schemes for ab initio total-energy calculations using a plane-wave basis set. *Phys Rev B.* 1996; **54**: 11169-11186.

[10]. Kresse G, Hafner J. Ab initio molecular-dynamics simulation of the liquid-metal--amorphous-semiconductor transition in germanium. *Phys Rev B.* 1994; **49**: 14251-14269.

[11]. Perdew JP, Burke K, Ernzerhof M. Generalized gradient approximation made simple. *Phys Rev Lett.* 1996; **77**: 3865-3868.

[12]. Guo R, Hu M, Zhang W *et al*. Boosting electrochemical nitrogen reduction performance over binuclear Mo atoms on N-doped nanoporous graphene: A theoretical investigation. *Molecules*. 2019; Vol. **24**.

[13]. Blöchl PE, Projector augmented-wave method. *Phys Rev B*. 1994; **50**: 17953-17979.

[14]. Grimme S. Semiempirical GGA-type density functional constructed with a long-range dispersion correction.

J Comput Chem. 2006; **27**: 1787-1799.

[15]. Grimme S, Antony J, Ehrlich S *et al*. A consistent and accurate ab initio parametrization of density functional dispersion correction (DFT-D) for the 94 elements H-Pu. *J Chem Phys.* 2010; **132**: 154104.

[16]. Grimme S, Ehrlich S, Goerigk L. Effect of the damping function in dispersion corrected density functional theory. *J Comput Chem.* 2011; **32**: 1456-1465.

[17]. Frisch MJ, Trucks GW, Schlegel HB *et al*. Gaussian 16. Gaussian, Inc. Wallingford, CT: 2016.

[18]. Miertuš S, Scrocco E, Tomasi J. Electrostatic interaction of a solute with a continuum. A direct utilizaion of AB initio molecular potentials for the prevision of solvent effects. *Chem Phys.* 1981; **55**: 117-129.

[19]. Zhao Y, Truhlar DG. The M06 suite of density functionals for main group thermochemistry, thermochemical kinetics, noncovalent interactions, excited states, and transition elements: two new functionals and systematic testing of four M06-class functionals and 12 other functionals. *Theor Chem Acc.* 2008; **120**: 215-241.

[20]. Lu T, Liu Z, Chen Q. Comment on "18 and 12 – member carbon rings (cyclo[n]carbons) – A density functional study". *Mater Sci Eng B.* 2021; **273**: 115425.

[21]. Lu T, Chen Q. Independent gradient model based on Hirshfeld partition: A new method for visual study of interactions in chemical systems. *J Comput Chem.* 2022; **43**: 539-555.

[22]. Lu T, Chen F. Multiwfn: A multifunctional wavefunction analyzer. *J Comput Chem.* 2012; **33**: 580-592.

[23]. Legault CY. *CYLview*. 2009. http://www.cylview.org (accessed).
